# Differentiation of action mechanisms between natural and synthetic repellents through neuronal electroantennogram and proteomic in *Aedes aegypti* (Diptera: Culicidae)

**DOI:** 10.1038/s41598-022-24923-x

**Published:** 2022-11-27

**Authors:** Johan Sebastián Portilla Pulido, Diana Lizeth Urbina Duitama, María Carolina Velasquez-Martinez, Stelia Carolina Mendez-Sanchez, Jonny Edward Duque

**Affiliations:** 1 Grupo de Investigación en Bioquímica y Microbiología (GIBIM), Facultad de ciencias, Escuela de Química, Universidad Industrial de Santander, Bucaramanga, Colombia; 2 Departamento de Ciencias Básicas, Grupo de investigación en Neurociencias y Comportamiento UIS-UPB, Facultad de Salud, Escuela de Medicina, Universidad Industrial de Santander, Bucaramanga, Santander Colombia; 3 Departamento de Ciencias Básicas, Centro de Investigaciones en Enfermedades Tropicales-CINTROP, Facultad de Salud, Escuela de Medicina, Universidad Industrial de Santander, Bucaramanga, Santander Colombia

**Keywords:** Proteins, Animal physiology

## Abstract

Natural-based compounds with repellent activity arise nowadays with the possibility to replace commercial synthetic repellents wholly or partially, such as *N,N*-Diethyl-m-toluamide (DEET). It is due to DEET's demonstrated toxicity and cutaneous irritation for human beings. Besides, research recommends avoiding using it with kids and pregnant women. The search for a repellent product implies early stages of detailed research that resolve the modes of action against the target insect. Therefore the objective of the current study was to analyze neuronal electrophysiological signals and olfactory system protein expression when the *Aedes aegypti* mosquito with exposition to natural-based repellents. Adult females of *Ae. aegypti* of Rockefeller strain were exposed to specific concentrations of repellent compounds like geranyl acetate, α-bisabolol, nerolidol, and DEET. The neuronal effect was measured by electroantennography technique, and the effect of exposure to either DEET or a mixture of natural molecules on protein expression was determined with 2D-PAGE followed by MALDI-TOF-mass spectrometry (MS). This approach revealed that DEET affected proteins related to synapses and ATP production, whereas natural-based repellents increased transport, signaling, and detoxification proteins. The proteomic and electrophysiology experiments demonstrated that repellent exposure disrupts ionic channel activity and modifies neuronal synapse and energy production processes.

## Introduction

Personal protection to avoid mosquito bites and the diseases they transmit includes using repellents. Synthetic repellent substances consisting of DEET (*N,N*-Diethyl-m-toluamide), IR3535 (Ethyl butyl-acetyl-amino propionate), Picaridin ((1-(1-methyl propoxy-carbonyl)-2-(2-hydroxy ethyl) piperidine), and natural-based compounds derived from essential oils^1–3^. Nevertheless, it is highlighted that the action and reception mechanism is unclear due to the lack of information regarding the proteins and molecules involved in this process. Notwithstanding some research that has found some characteristics of insect olfactory systems, the knowledge about the repellent effect is slight, and its molecular effect needs to be more investigated. The repellent effect might be related to interactions with transport and signaling proteins like odorant-binding proteins and G-proteins in the mosquito olfactory system^4–6^.

The olfactory reception mechanism for repellent molecules in *Ae. aegypti*, the primary vector of diseases such as dengue, chikungunya, and Zika, is poorly elucidated. Also, it is vital to find a screening system to discover new molecules and develop natural repellent. According to that, some scientific research is focused on the odorant proteins reception model from *Drosophila melanogaster* to understand how repellents work in vector insects^7,8^. Repellent-insect interaction starts when the volatile chemical compounds interact with the chemoreceptors of the mosquito antennae triggering behavior that avoids the repellent molecules. After that, the compounds are transported through the nervous system by the action of Odorant Binding Proteins (OBPs). These OBPs can catch, solubilize, and transport volatile molecules through hydrophobic and hydrophilic interactions in their binding side^9–11^.

Once the primary activation of the message by the sensilla begins, the transport of these molecules leads to ligand-ionic channel or receptor interactions, triggering a transformation from a chemical signal into an electrical signal. The mentioned electrical signals can be analyzed and monitored by electrophysiological techniques such as electroantennography (EAG). This methodology offers a strategy to observe behavior cues related to the interaction between the mosquito and molecules with repellent effect. In this sense, some research has tried to elucidate electroantennographic response by observing the changes in the electrical signal due to different molecules like attractants, hormones, or pheromones^12,13^. Adding to this, it is unclear how the repellent molecules change the electrical signaling response in the mosquito. Due to this lack of information about the perception of repellent molecules by mosquitoes and what the electroantennography technique provides, it is possible that incorporating proteomic methodologies could increase the possibility of knowing more about action mechanisms^14^.

The proteomic and genomic research considering *Ae. albopictus*, *Ae. aegypti*, and *Anopheles gambiae* mosquitoes had identified several proteins involved in olfactory processes^14,15^. Despite that, it remains unclear how proteins and enzymes altogether perform their function in the olfactory system during molecule reception. This research's main objective was to comprehend the electrochemical reception induced by repellents in the *Ae. aegypti* mosquito using electroantennographic assays and identifying proteins involved in repellent olfactory reception using proteomics. This study contemplates setting a path to design repellents in future research that can replace commercial repellents such as DEET and IR3535 which have toxic effects on humans.

## Methodology

### Biologic material: *Aedes aegypti* colony

Electroantennography and proteomic experiments were carried out using an *Aedes aegypti* colony, Rockefeller strain. The mosquitoes were disposed of in insectary security cages, with a temperature of 25 ± 5 °C and humidity of 70 ± 5%, all cages were set in a 12:12 h photoperiod. Male mosquitoes were fed with a honey-water solution approximately 10% (v/v). Female mosquitoes were periodically supplied with blood meal using Wistar albino rats (WI IOPS AF/Han strain) to obtain mosquito eggs. The rodent animals were offered by the vivarium of the Industrial University of Santander, accomplishing laws decreed by the Colombian Health Ministry. Complying with the laboratory animal handling procedures and under the approval of the Ethics Committee “*Comité de ética en investigación científica de la Universidad Industrial de Santander CEINCI*”: Scientific Research Ethics Committee of the Industrial University of Santander (acronym in spanish CEINCI); Minutes No. 08, May 11, 2018). The mosquito eggs were hatched, and larvae were disposed of in plastic containers with water and 0.5 g of fish food (Tetramin Tropical Flakes^®^). The food was added three times a week. To use the mosquitoes for Electroantennography and proteomic assays, female adult mosquitoes were selected without feeding between 3 and 10 days after emerging from the larvae stage. Furthermore, the study was carried out in compliance with the ARRIVE guidelines, and we indicate that all experiments were performed in accordance with relevant guidelines and regulations.

### Molecules

**Figure 1:**
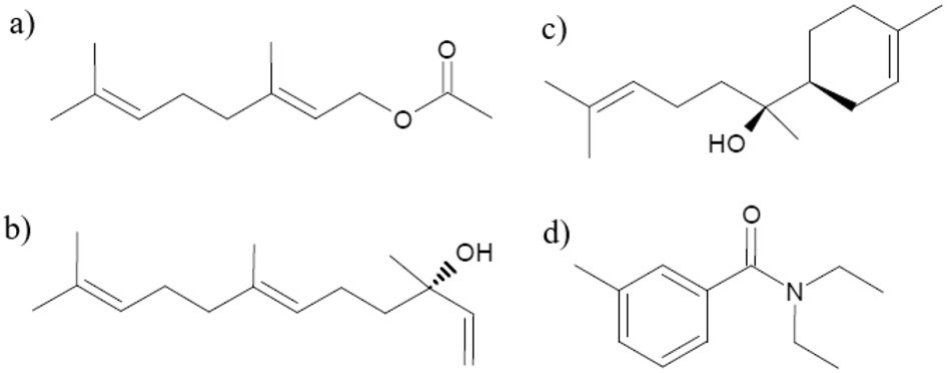
Molecules with repellent activity implemented in the electroantennography and proteomic assays (**a**) Geranyl acetate, (**b**) Nerolidol, (**c**) α-bisabolol, (**d**) DEET.

The molecules evaluated in this research were selected previously from Essential Oils (EOs) extracted from plants with potential repellent activity and tested by Centro de Investigaciones en Enfermedades Tropicales (CINTROP). The terpenoid compounds selected and with efficient repellent activity were the secondary metabolites: Geranyl acetate, α-bisabolol, and Nerolidol, purchased from Merck ©. The mentioned compounds have repellent activity confirmed and previously reported by CINTROP^16^. The synthetic repellent DEET was used as a positive control (Fig. 1). All chemical reagents used in this work were purchased from Merck ©, USA.

### Electroantennographic experiments

#### Insect preparation and electroantennographic recording

**Figure 2:**
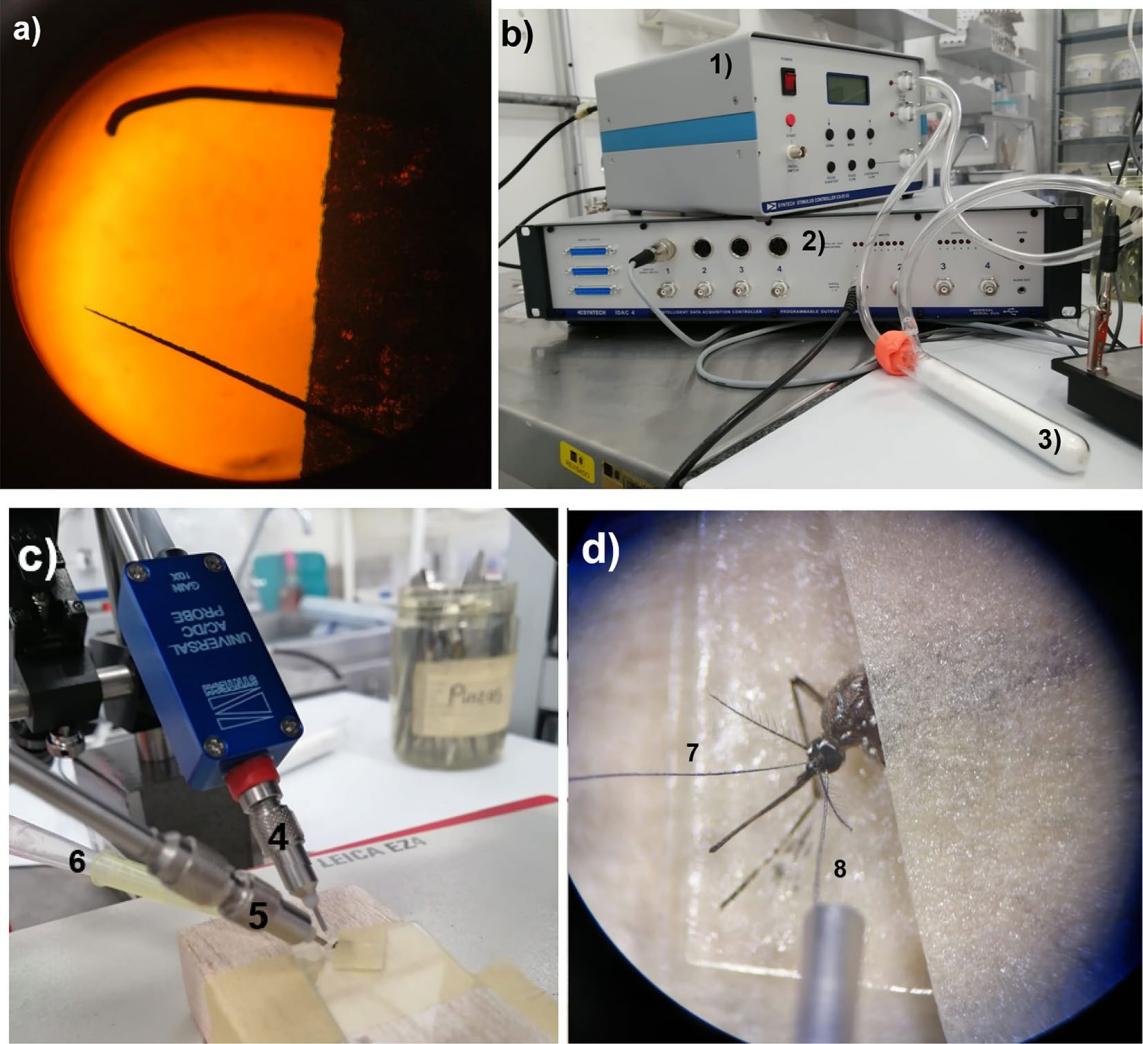
Electroantennographic assays set for mosquitos. (**a**) Tungsten electrodes electrolytic sharpening with NaOH (above the non-sharpen electrode, below sharpen electrode). (**b**) The Electroantennography equipment consists of an air flux system that allows transporting the volatile compounds directly to the insect preparation (1), also, a system of data acquisition (2) (Synthec). Besides, it is shown the compartment where the filter paper impregnated with the interested substances is put (3). Through the test tube with hose connection, the air flux is transported to the insect preparation. (**c**) The electrodes are positioned in holders allowing easy manipulation in the x, y, z coordinate system. (4) The recording electrode is embedded in the mosquito’s antennae and the reference electrode in the insect’s eye (5). Moreover, from the test tube with hose connection (3), the air flux with the stimulus (molecules) is transported to (6) where the air is finally released in the insect preparation. (**d**) The embedded electrodes are shown in (7) for the recording electrode and (8) for the reference electrode. Images: Author.

We took female mosquitoes between 3 to 10 days from emerging and without feeding. Each insect was anesthetized, setting the mosquitoes in a freezer (− 5 °C) for 1 min. Subsequently, the insect was set in a microscope slide with scotch tape, as shown in Fig. 2c,d. Then, the mosquito was set in a stereomicroscope Leica EZ4 (Leica Microsystems (Schweiz) AG Heerbrugg, Switzerland) considering that the mosquito’s eyes and antennas needed to be exposed. The reference electrode was embedded in the mosquito’s eye exposed, while the recording electrode was inserted in the base of the antennae. Recording and reference electrodes made of Tungsten in all processes were implemented (0.25 mm, Merck ©). The electrodes were electrolytically sharpened through repeated immersion of the Tungsten electrode in a sodium hydroxide solution 1 M NaOH, 3–5 V until obtaining a diameter approximate to 0.05–1.0 mm^12^ (Fig. 2a). The electroantennographic recording started 2 min after the mosquito was set in the stereomicroscope with the electrodes. The corresponding electroantennographic signals were amplified (100×) with a preamplifier (Universal Single Probe, type PRS-1, Syntech, Germany) and digitized (IDAC-4, Syntech, Germany). After that, the electrical signals were visualized, recorded, and analyzed in a computer using the Autospike Software (Syntech, Germany)^12^.

#### Olfactory stimulus and electroantennogram recording

The air pulses were transported using an essay tube and hose connections. Then, air flux was administered with filtered air to eliminate the background, then injected the repellent volatiles (Fig. 2b). Each repellent compound was impregnated in a small piece of filter paper (Whatman No. 1) in different concentrations of 0.5, 1.0, 10, 50, and 100 mg/mL, using acetone as solvent. After that, the paper was allowed to dry for 1 min to volatilize the acetone solvent^12^. Besides, ammonia at 25% (v/v) was used as a control before and after each compound tested. We used ammonia since it is an attractant compound for mosquitoes, and its signal can be visualized in the software in the mV scale^17^. In this first part of the process, a single mosquito was used for all the concentrations between 0.5 and 100 mg/mL with an initial and final ammonia stimulus. Also, we injected 8 s of pulses of filtered air in between each concentration. A filter paper was set in the assay tube with a hose connection engaged to the air flux. We used a different essay tube and a new insect preparation (a mosquito per replicate, with a minimum of N = 7 per experiment). Additionally, mixtures of the natural-based compounds were evaluated in the electroantennographic assays in the concentrations of 50 and 100 mg/mL. Each mixture concentration was applied in separated assays compared to the previous experiments with 0.5–100 mg/mL. Mixture 1: geranyl acetate, α-bisabolol, nerolidol; Mixture 2: geranyl acetate and nerolidol; Mixture 3: nerolidol and α-bisabolol; mixture 4: geranyl acetate and α-bisabolol. For these experiments, two mosquito sets were prepared for the 50 mg/mL and another sample for the 100 mg/mL with each mixture and replicate executed.

The compounds with repellent action, geranyl acetate, α-bisabolol, nerolidol, and DEET were administered by a controlled air flux (Stimulus controller unit, type CS-55, Syntech, Germany) to evaluate the electrical signals in the mosquitoes. The air flux was programmed with three sequences of air pulses: 25 mL/s air flux rate, ten seconds each pulse with intervals of two seconds for the air flux with the repellent compounds, and eight seconds with only background air. To guarantee the insect stimulation by this attractant molecule and the effectiveness in the electrode connection as mentioned, ammonia (25% v/v) pulse was before and after applying the repellent compounds. DEET was applied as a positive control (0.5–100 mg/mL) since it is a synthetic repellent with high time protection against mosquitoes' bites. In addition to the previous experiments, we simultaneously release air flux with ammonia (attractant) and repellent molecules. Therefore, a change in the initial signal of ammonia was observed and analyzed. Corresponding to patterns in the electrical signals that record the initial and final changes in the ammonia stimulus due to the exposition with repellent molecules for the simultaneous release of ammonia and repellent, the compounds geranyl acetate, α-bisabolol, nerolidol, DEET, mixtures 1, 2, 3, and 4 were used in the experiments at concentrations of 50 and 100 mg/mL. Firstly, the ammonia stimulus was administered, followed by the repellent-ammonia pulse. The previous montage was implemented with a single mosquito per concentration and molecule with at least N = 7 replicates per experiment.

### Proteomic analysis

#### Mosquito treatment

120–140 adult female *Ae. aegypti* mosquitoes between 3 and 10 days from emerging, without blood-feeding, were set in security cages and completely sealed with plastic stretch film. The purpose of sealing the cage was to avoid the volatilization of repellent molecules inside the cage and keep the same concentration of repellent along the mosquito treatment time. In acetone as a solvent, a filter paper was impregnated with the repellent molecules at 100 mg/mL. The mixture 1 (geranyl acetate, α-bisabolol, nerolidol, 100 mg/mL each) and DEET repellent as positive control (100 mg/mL). After applying the repellent, the filter paper was to dry for 5 min until the acetone solvent was wholly volatilized. Then, the mosquitoes and the filter paper were set in the sealed cage for 45 min of exposition. This time was set since, on average, in vivo repellent assays carried out in previous research lasted 1 to 3 h^16^, and the electroantennography (EAG) experiments lasted at least 10–15 min. Thereby, 45 min was established as an intermediate time to study the protein expression in early stages related to the repellent mechanism process. Finally, the sealed cage was placed in a freezer at − 20 °C for 10 min to sacrifice the mosquitoes and maintain the integrity of cell tissues avoiding protein degradation. Non-treated mosquitoes were used as a negative control (no repellent exposure) for protein extraction.

#### Protein extraction

The proteins were extracted from 120 to 140 female adult *Ae. aegypti* mosquito’s head between 3 and 10 days from emerging, without a blood-feeding. The mosquito heads were cut from the mosquito’s body with entomological forceps (Biologika) and using a stereoscope (Leica zoom 2000). Subsequently, the mosquito heads were put in an Eppendorf vial with 250 μL of lysis buffer (7 M Urea, 2 M Thiourea, 0.5% Triton X-100, 0.5 pharmalyte, DTT 20 mM, CHAPS 4% p/v) at 4 °C. The mosquito’s heads were homogenized with a Van Potter homogenizer to extract the maximum amount of protein available from tissues. The Eppendorf vial was centrifuged at 12,000×*g* and 4 °C for 15 min (Microfuge^®^ 20R Centrifuge), the supernatant liquid with the protein was taken. Finally, the Bradford method determined the protein concentration^18^ using a calibration curve of Bovine Serum Albumin (BSA). The protein sample was stored in a freezer at − 75 °C previously to be used for Two-dimensional SDS Polyacrylamide Gel Electrophoresis (2D SDS Page)^19^.

#### Two-dimensional SDS polyacrylamide gel electrophoresis (2D SDS-PAGE)

##### Electrophoresis isoelectric focusing IEF gel

For the IEF gels, immobilized pH gradient strips (IPG) in the pH range from 3 to 10, ReadyStrip™ IPG Strips, BioRad, Laboratories, Inc, USA. Were hydrated with the rehydration buffer (7 M Urea, 2 M Thiourea, 1% CHAPS, 20 mM DTT, 0.25% ampholytes pH 3–10, and bromophenol blue 0.1%). The IPG strips were rehydrated with the buffer for 16 h. After that, the strips transferred to an Isoelectric focusing cell (Protean i12 IEF Cell, Bio-Rad Laboratories, Inc., USA). A ramp or linear gradient of voltage was established as follows between 300 and 10,000 V: 100 V for 20 min, 4000 V for 120 min, and 10,000 V for 60 min.

##### Equilibrium and SDS page electrophoresis gel

Before using the SDS page gel sample, the IPG strips were treated with an equilibrium buffer for 15 min with stirring. The first equilibrium buffer contained 50 mM Tris–HCl pH 8.8, 6 M Urea, 20% glycerol, 2% SDS, and 1% DTT. The second equilibrium buffer contained iodoacetamide at 2.5% instead of DTT. After the equilibration process, the polyacrylamide gel at 12.5% was prepared in an electrophoresis cell (Bio-Rad), then the IPG strip was set in the cell. To this montage, a loading buffer was added (100 mM Tris–HCl pH 6.8, SDS 4% w/v, bromophenol blue 0.2%, DTT 200 mM). Finally, a power supply (Bio-Rad) was connected for 1 h at 150 V^20^.

Once obtained, the gel with the separated proteins was stained with Coomasie blue G250, then the gels were scanned in a Gel Documentation system (Gel-doc Bio-Rad)^21^ with a white Sample Tray for ChemiDoc™ MP/ChemiDoc Imaging Systems. Triplicates performed the bidimensional gels for each treatment. After that, spots were analyzed using the PDQuest Software, to determine optical density intensity differences. Those spots with significant statistical differences according to the foldchange parameter were selected. This value showed a relative value related to the change in the protein expression considering the control (non-treated mosquitoes). Thereby, overexpressed proteins were identified by comparing the foldchange ≥ 2.0 in relative intensity, and under-expressed proteins with a foldchange ≤ 0.5 in relative intensity. The comparisons control vs DEET and control vs mixture 1 (geranyl acetate, α-bisabolol, nerolidol) were performed.

#### Comparison and protein identification

The protein spots found with differential expression, under or overexpressed, were selected to be analyzed in a Mass Spectrometer MALDI TOF-TOF Bruker Ultraflextreme (Bruker Daltonics, MA, USA), equipped with a Smart Beam Nd:YAG solid-state laser (l = 355 nm), with a frequency of 1 kHz and a maximum energy output of approximately 85 µJ per shot, a pulse duration of 6 ns with a spot width of 10 µm to 100 µm, according to the manufacturer's specifications. The selected spots were extracted, then, the Coomassie Blue G250 dye was removed using acetonitrile (ACN) at 50% v/v and trifluoroacetic acid (TFA) at 1% v/v. Thereafter, the enzyme trypsin was used to digest the proteins and obtain smaller peptides. After tryptic treatment, the peptides obtained were seed in a mass spectrometer target (Bruker) using a supersaturated α-cyano-hydroxycinnamic acid (HCCA) solution, using as solvent ACN 50% and TFA 1%, the volume ratio sample/matrix was 1:1. The peptide fingerprints of proteins were analyzed in the mass spectrometer at the Industrial University of Santander. The patterns used as a reference to calibrate the mass spectrometer equipment were bombesin, bradykinin, renin substrate, and leu-enkephalin. The mass spectrometry spectra were visualized in the Flexanalysis software.

The mass spectra were subsequently analyzed in the online software Mascot (http://www.matrixscience.com) using the databases of SwissProt to identify the proteins according to the score obtained per spectra. The parameters to obtain name and protein score were as follows: Database SwissProt, Enzyme digestion with trypsin, taxonomy: all entries available, fixed modifications: carbamidomethyl (C), variable modifications: oxidation, peptide tolerance: different values 0.5, 1.0 Dalton (Da), in total, six possible proteins per spectra analyzed were obtained. The function was searched for each possible protein in the Uniprot database, and the classification was according to the functions like transport, energy production, signaling, regulation proteins, etc. The protein selection was made according to the score obtained per spectra, expect value and % sequence coverage. The protein score indicates the probability that the observed match is a random event. The Expect value indicates the probability that the observed match between spectra and peptide sequence would be found by chance. Confident matches typically have Expect values < 0.1^22^.

#### Protein–protein interaction

The identified proteins through MALDI-TOF mass spectrometry were selected to realize an analysis of protein interactions. The online software STRING (https://string-db.org/) was used to research those interactions. The list of the proteins was introduced in the software, selecting the housefly, *Drosophila melanogaster*, as a reference organism. Regarding the advanced adjustments, it was selected the whole string network (network type), medium confidence (relative value 0.4), medium-scale (5%) for statistical error. Other modifications in style and size were realized with Cytoscape software^23^.

#### Statistical analysis

The electrical signals obtained by the electroantennography equipment were calculated considering the average in the maximum amplitude in the mV scale. The electroantennographic signals were compared according to the change in the scale in mV or µV considering the ammonia signals before and after applying the repellent stimulus. The data were put through normality tests such as Kolmogorov-Smirnnorv, Shapiro–Wilk, and Lillieford. An ANOVA test was applied to the normally distributed data, subsequently, a Tukey test or t-student test when necessary. According to the above, the t-student test was used to compare two mean average data sets. If the data distribution is present as non-normal, a non-parametric test (Kruskal–Wallis) was applied. The comparisons were considered statistically different if the p-value was < 0.05. The Statistic V11 (Statsoft) and GradPad Prism 5 were used to analyze the statistical analysis.

## Results

### Electroantennography

#### Electroantennography repellent compounds recordings

The molecules evaluated in electroantennography (EAG) registered electrical signals from 0 to 200 μV. The ammonia applied initially showed high values in the μV scale, from 1197 to 1983 μV. Additionally, the final ammonia pulse applied at the end of the experiment showed a percentage reduction from 36.5 to 79% when the ammonia electrical signals at the start and finalizing the experiment were compared (Table 1).

**Table 1:** Electroantennography signals (microvolts ± standard deviation, µV ± SEM) for the repellent compounds: geranyl acetate, α-bisabolol, nerolidol, and DEET with a pre- and post-treatment ammonia stimulus.

Molecule	EAG response (µV ± SEM)							
	Initial NH_3_	0.5 mg/mL	1 mg/mL	10 mg/mL	50 mg/mL	100 mg/mL	Final NH_3_	Reduction (%)
Geranyl acetate	1197.0 ± 216.0	168.6 ± 34.6	177.1 ± 43.5	154.3 ± 47.0	125.7 ± 34.6	117.1 ± 28.1	911.4 ± 183.5	36.5 ± 10.2
α-bisabolol	1940.0 ± 120.6	0.0 ± 0.0	0.0 ± 0.0	0.0 ± 0.0	0.0 ± 0.0	0.0 ± 0.0	397.1 ± 115.4	79.5 ± 6.0***
Nerolidol	1983.0 ± 170.1	194.3 ± 47.0	200.0 ± 24.3	160.0 ± 28.3	145.7 ± 16.2	134.3 ± 18.4	574.3 ± 103.8	71.0 ± 5.2***
DEET	1757.0 ± 200.3	171.4 ± 44.0	177.1 ± 62.4	128.6 ± 48.8	128.6 ± 49.5	117.1 ± 28.8	997.1 ± 226.2	43.2 ± 12.9**

For each molecule and concentration, the percentage changes in the values regarding the pre- and post-treatment ammonia signal are shown. (**) and (***) indicate statistically significant differences comparing the EAG ammonia signals before and after applying the repellent molecules in the EAG essays. One way ANOVA, (**) p < 0.01; (***) p < 0.001, post hoc Tukey test.

**Figure 3:**
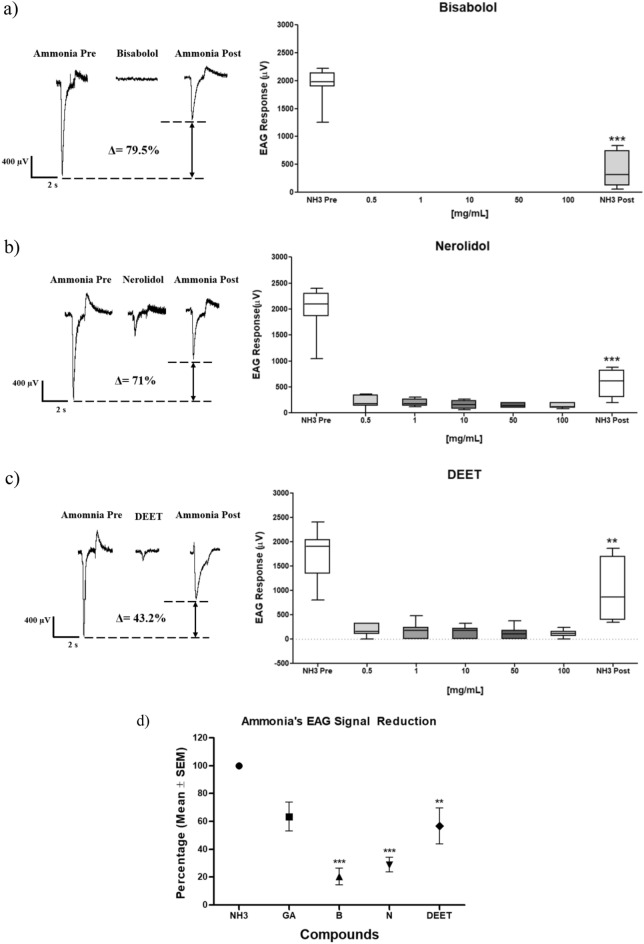
Electroantennography signals patterns and percentage changes in the post-treatment ammonia signals after applying the molecules, (**a**) α-bisabolol, (**b**) nerolidol y, (**c**) DEET in the concentrations 0.5, 1.0, 10, 50, and 100 mg/mL. For each experiment, a pre- and post- pulse of ammonia was applied. (**d**) Percentual reduction summary (average ± standard deviation SEM) of ammonia stimulus in the olfactory mosquito system after applying the repellent substances mentioned (NH3: ammonia, GA: Geranyl Acetate, B: α-bisabolol, N: nerolidol). (**) and (***) indicate statistically significant differences comparing the ammonia EAG signals pre- and post-treatment before and after applying the repellent molecules, respectively. One way ANOVA, (**) p < 0.01; (***) p < 0.001, post hoc Tukey test.

After applying the repellent molecules, all experiments presented that the EAG signal regarding the ammonia post-treatment decreased significantly. Concerning Nerolidol (ANOVA F(6,42) = 73.36, p < 0.0001), and α-bisabolol (F(6,42) = 131.5, p < 0.0001), the post-treatment ammonia signals decreased significant after applying the repellent molecules (Fig. 3). This EAG signal decrease indicates the olfactory mosquito system's inhibition was caused for probability with the repellent molecules. Similarly, DEET repellent showed the same pattern, where the post-treatment ammonia signal decreased with statistically significant differences (ANOVA F(6,42) = 27.95, p < 0.0001) (Fig. 3). However, geranyl acetate showed no statistically significant differences comparing pre- and post-treatment ammonia signals changes in the μV scale (ANOVA F(6,42) = 16.19, p > 0.05) (Fig. S1).

In Fig. 3, EAG signal patterns are shown for each molecule evaluated and in different concentrations. It is shown that ammonia pretreatment exhibits high values in the microvolts (μV) scale. Additionally, a small peak or elevation after returning to the baseline was detected at the end of each ammonia pulse. This observation is attributed to the recovery or repolarization period in the neuronal membrane. In contrast, for each repellent molecule, the EAG signals presented values no more than 200 µV (Table 1). A percentage value is displayed in each figure and indicates the change in the numerical values regarding the pre-and post-treatment ammonia EAG signals. In the first set of experiments, α-bisabolol, DEET, and nerolidol changed in a greater ratio the post-treatment ammonia signal registered in comparison with the pre-treatment EAG signal.

### Repellent mixtures EAG recordings

Regarding mixture 1 to 4, prepared as follows: mixture 1 (geranyl acetate, α-bisabolol, and nerolidol), mixture 2 (geranyl acetate and nerolidol), mixture 3 (nerolidol and α-bisabolol), mixture 4 (geranyl acetate and α-bisabolol). In all cases, the same pattern in the EAG signals was evidenced. For each mixture, lower values in the µV were shown (135.0 and 277.8 µV) (Table 2).

**Table 2:** Electroantennography signals (microvolts ± standard deviation, µV ± SEM) regarding the mixtures 1, 2, 3, 4 in the 50 and 100 mg/mL concentrations using a single mosquito per replicate and concentration.

Mixture	EAG response (µV ± SEM)							
	Initial NH_3_	50 mg/mL	Final NH_3_	Reduction (%)	Initial NH_3_	100 mg/mL	Final NH_3_	Reduction (%)
1	1806.0 ± 55.0	220.0 ± 29.0	1351.0 ± 95.2	25.2 ± 5.3***	2355.0 ± 146.4	197.5 ± 23.7	1768.0 ± 193.3	25.0 ± 8.2*
2	1751.0 ± 56.1	277.8 ± 41.02	1476.0 ± 96.5	15.7 ± 5.5*	2154.0 ± 215.7	222.9 ± 24.8	1594 ± 146.2	26.0 ± 6.8**
3	2680.0 ± 255.6	245.7 ± 23.4	1857.0 ± 226.9	30.7 ± 8.5*	2760.0 ± 303.7	254.3 ± 29.2	1886.0 ± 258.8	32.0 ± 9.4*
4	1898.0 ± 213.6	155.0 ± 27.7	1045.0 ± 211.0	45 ± 11.1*	1995.0 ± 249.2	135.0 ± 33.33	1020.0 ± 152.9	51.0 ± 7.7*

In each experiment, an initial and final stimulus of ammonia was applied. Besides, in each concentration, it is shown the percentage reduction concerning the ammonia signal obtained after applying the mixtures mentioned. Mixture 1 (geranyl acetate, α-bisabolol, and nerolidol), mixture 2 (geranyl acetate and nerolidol), mixture 3 (nerolidol and α-bisabolol), mixture 4 (geranyl acetate and α-bisabolol). (*), (**), and (***) indicate differences statistically significant between EAG pre- and post-treatment ammonia signals. One way ANOVA, (*) p < 0.05; (**) p < 0.01; (***) p < 0.001, post hoc Tukey test.

**Figure 4:**
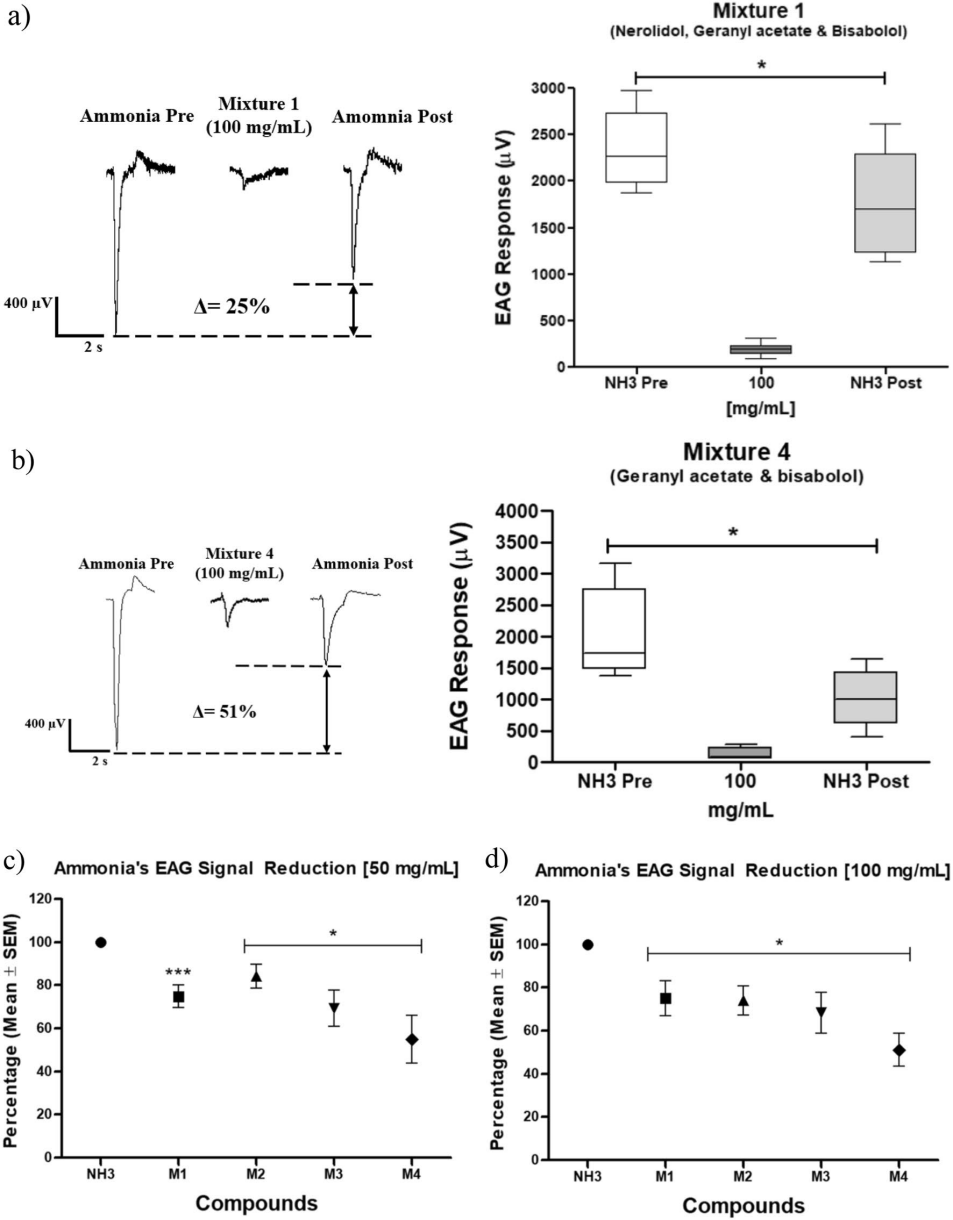
EAG signals patterns and percentage changes in the pre- and post-treatment ammonia signal after applying (**a**) mixture 1 [100 mg/mL] (geranyl acetate, α-bisabolol, and nerolidol), (**b**) mixture 4 [100 mg/mL] (geranyl acetate and α-bisabolol) with a pre- and post-treatment pulse of ammonia. (**c**) and (**d**) Percentual reduction of ammonia EAG signal summary for each mixture (mean average ± standard deviation SEM) in the concentrations 50 and 100 mg/mL, respectively. (NH_3_: ammonia, M1: mixture 1, M2: mixture 2, M3: mixture 3, M4: mixture 4). (*) Indicates statistically significant differences between pre- and post-treatment ammonia signals for the mixtures mentioned. One way ANOVA, (*) p < 0.05, post hoc Tukey test. Mixture 2 (geranyl acetate and nerolidol), mixture 3 (nerolidol and α-bisabolol).

In comparison with the application of the molecules individually, all mixtures in the 50 and 100 mg/mL concentrations showed amplitude changes in the post-treatment ammonia signal. Thus, the pre- and post-treatment ammonia signals presented statistically significant differences comparing both ammonia signals mentioned. Applying the mixture 1 evidenced statistically significant differences in both concentrations (Fig. 4), mixture 1 [50 mg/mL] (ANOVA F(2,18) = 154.8, p < 0.0001), mixture 1 [100 mg/mL] (F(2,21) = 62.86, p < 0.0001). In similar fashion, the mixtures 2 and 3 (Fig. S2), mixture 4 (Fig. 4) at 50 mg/mL (ANOVA F(2,21) = 25.06, p < 0.0001), and mixture 4 at 100 mg/mL (ANOVA F(2,21) = 29.99, p < 0.0001) showed differences in the pre- and post- ammonia treatment. Comparing the above results in Fig. 3 related to individual molecules, with repellent mixtures (Figs. 4, S2), it is noticed that post-treatment ammonia signal changes in a greater proportion implementing all the concentrations in a single experiment (0.5, 1.0, 10, 50, and 100 mg/mL) (Fig. 3). This treatment indicates that olfactory mosquito capability decreases since the mosquito is exposed to repellent compounds for a more extended period. Therefore, it hinders the electrical signals and ion flux through the neuronal membrane.

The same signal reduction pattern was shown in all treatments concerning the ammonia signal changes due to the repellent exposition. Firstly, the pre-treatment signal presented higher values in the µV scale. However, after applying the repellent mixture, the ammonia signal decreased significantly, as evidenced before. Figure 4 exhibits the EAG patterns obtained in mixtures 1 and 4. The results in this section regarding the ammonia electrical signals after applying the repellent compounds were similar to the individual compound application. All repellent mixtures changed the post-treatment ammonia signal statistically significantly. This response implies that applying the repellent molecules individually or in mixtures creates an olfactory system inhibition in the mosquito.

### EAG recordings: ammonia and repellent simultaneous stimulation

The electrical signals exposing the mosquito to the repellent and the ammonia were recorded simultaneously to simulate conditions of repellency. As expected, the EAG patterns obtained were similar to the previous experiments (Figs. 3, 4, Table 3). The same pattern was found; the amplitude values regarding the post-treatment stimulus (ammonia-repellent simultaneously) were decreased in each substance and concentration.

**Table 3:** Electroantennography signals (microvolts ± standard deviation, µV ± SEM) concerning the molecules geranyl acetate, α-bisabolol, nerolidol, DEET, and the mixtures 1, 2, 3, 4 in the 50 y 100 mg/mL concentrations applying a single mosquito per replicate and concentration.

Molecule	EAG response (µV ± SEM)		
	Initial NH_3_	Final NH_3_ (ammonia + repellent)	Reduction (%)
Geranyl acetate (50 mg/mL)	1498.0 ± 194.2	1345.0 ± 262.9	11.0 ± 9.2
Geranyl acetate (100 mg/mL)	1733.0 ± 195.9	1151.0 ± 104.8	33.6 ± 6.0*
α-bisabolol (50 mg/mL)	2045.0 ± 283.6	1433.0 ± 163.3	30.0 ± 8.0*
α-bisabolol (100 mg/mL)	2463.0 ± 255.6	1183.0 ± 208.9	52.0 ± 8.5*
Nerolidol (50 mg/mL)	1446.0 ± 92.1	982.9 ± 130.0	32.0 ± 9.0*
Nerolidol (100 mg/mL)	1998.0 ± 157.9	1028.0 ± 152.6	49.0 ± 7.6*
DEET (50 mg/mL)	1823.0 ± 221.0	1240.0 ± 119.4	32.0 ± 6.5
DEET (100 mg/mL)	1993.0 ± 210.8	1278.0 ± 166.0	36.0 ± 8.3*
Mixture 1 (50 mg/mL)	2720.0 ± 367.7	1718.0 ± 365.7	37.0 ± 13.4
Mixture 1 (100 mg/mL)	1834.0 ± 195.2	802.9 ± 99.1	56.0 ± 5.4*
Mixture 2 (50 mg/mL)	3147.0 ± 257.5	2260.0 ± 537.7	28.0 ± 17.0
Mixture 2 (100 mg/mL)	1749.0 ± 34.3	1437.0 ± 87.4	18.0 ± 5.0*
Mixture 3 (50 mg/mL)	1360.0 ± 61.72	1003.0 ± 107.4	26.0 ± 7.9*
Mixture 3 (100 mg/mL)	2069.0 ± 314.5	1397.0 ± 184.8	36.0 ± 8.3*
Mixture 4 (50 mg/mL)	1520.0 ± 115.7	1009.0 ± 84.6	34.0 ± 5.6*
Mixture 4 (100 mg/mL)	1886.0 ± 173.0	1371.0 ± 217.2	28.0 ± 11.5*

In each experiment, an initial ammonia pulse and then an ammonia-repellent pulse was applied. For each molecule and mixture in different concentrations, the percentage reduction in the post-treatment EAG ammonia signal is displayed. Mixture 1 (geranyl acetate, α-bisabolol, and nerolidol), mixture 2 (geranyl acetate and nerolidol), mixture 3 (nerolidol and α-bisabolol), and mixture 4 (geranyl acetate and α-bisabolol). (*) indicates statistically significant differences between the initial ammonia signal and the ammonia-repellent signal. One-way ANOVA, t-student test.

**Figure 5:**
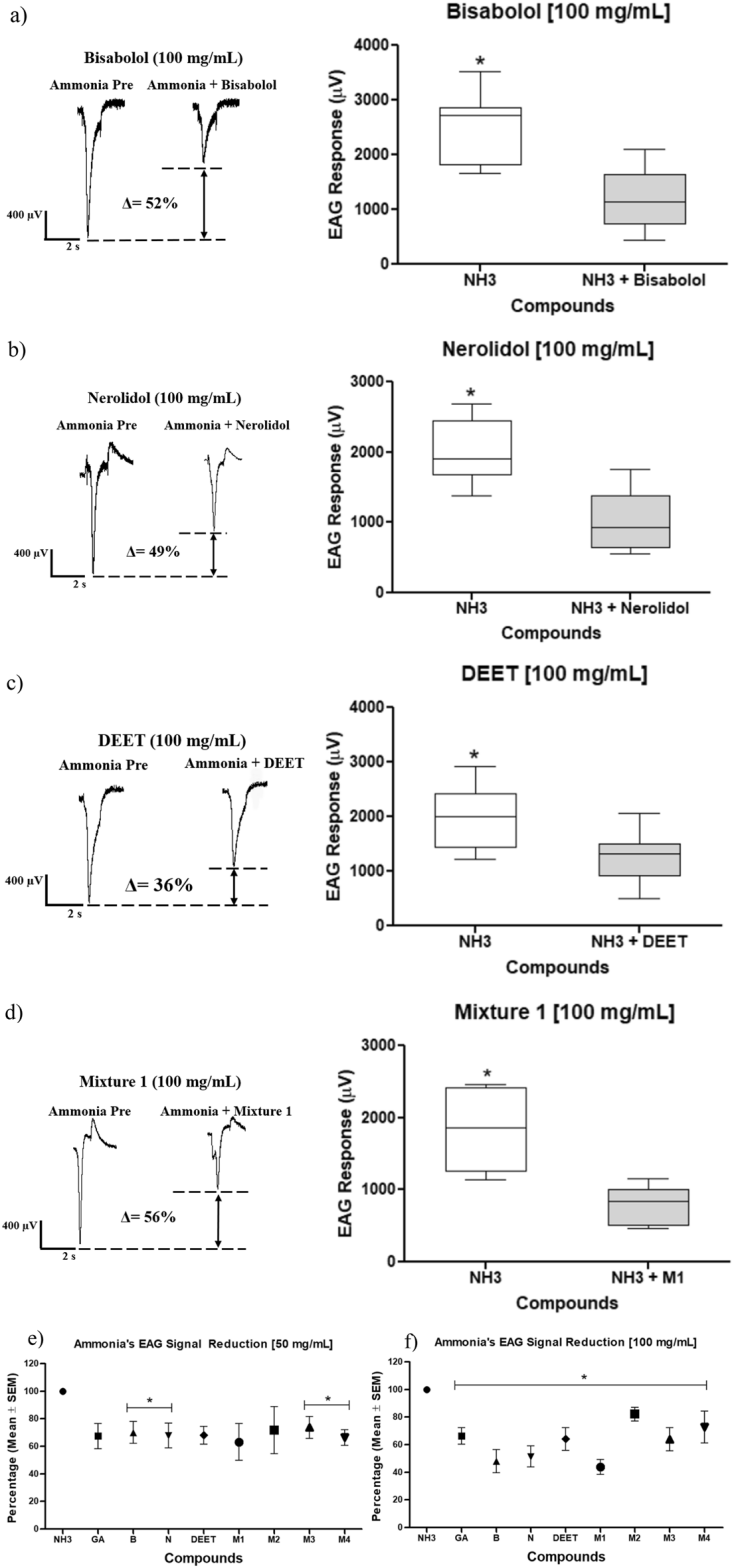
Electroantennography signal patterns and percentage changes in the electrical signals after applying the ammonia and the repellent stimulus simultaneously. (**a**) α-bisabolol 100 mg/mL, (**b**) Nerolidol 100 mg/mL, (**c**) DEET 100 mg/mL and (**d**) Mixture 1 [100 mg/mL]. The ammonia-repellent stimulus was applied after an initial pulse containing only ammonia. (*) Indicates statistically significant differences between the pre-treatment ammonia stimulus in comparison with the ammonia-repellent stimulus. One way ANOVA, t-student test, α-bisabolol) 100 mg/mL (t(6) = 4231; p = 0.0055, t-test); Nerolidol 100 mg/mL (t(7) = 5129; p = 0.0014, t-test); DEET 100 mg/mL (t(7) = 2883; p = 0.0236, t-test); mixture 1 [100 mg/mL] (t(6) = 5824; p = 0.0011, t-test). (**e**) and (**f**) Percentual reduction EAG signal summary regarding the exposition to the molecules and mixtures in the 50 mg/mL and 100 mg/mL concentration, respectively. (NH_3_: ammonia, GA: geranyl acetate, B: α-bisabolol, N: nerolidol, DEET, M1: mixture 1, M2: mixture 2, M3: mixture 3, M4: mixture 4).

The substances in the 100 mg/mL concentration, excluding the mixtures 2 and 4 (Fig. S4), decreased in a greater proportion the percentual ammonia signal applied initially with statistically significant differences. The molecules geranyl acetate (50 mg/mL), DEET (50 mg/mL), and mixtures 1 and 2, both at 50 mg/mL, showed no statistically significant differences (Fig. S4). The above infers that the simultaneous application of ammonia and repellent in this concentration shows no changes in the perception of mosquito olfactory. However, some of the other molecules and mixtures at 50 and 100 mg/mL showed statistically significant differences if the ammonia was applied simultaneously with the repellent. The substances geranyl acetate, DEET, the mixtures 1 and 2 at 100 mg/mL, and α-bisabolol, nerolidol, mixture 3 at both concentrations (50 and 100 mg/mL), showed statistically significant differences comparing the initial ammonia stimulus and the ammonia-repellent simultaneous stimulus. The statistical t-student values for each set of experiments are displayed in Figs. 5 and S4.

We might infer notorious differences in the mosquito’s olfactory perception against repellent compounds, just with greater percentages changes. For instance, with more than 30% was more notorious the change in the post-experiment ammonia EAG signal: geranyl acetate (33.6% ± 6.0), α-bisabolol (52% ± 8.5), nerolidol (49% ± 7.6), mixture 1 (56% ± 5.4), mixture 3 (36% ± 8.3) at a concentration of 100 mg/mL and the mixture 4 (34% ± 5.6) at 50 mg/mL. Concerning the mentioned results, it is highlighted that mixture 1, was previously evaluated with in vivo experiments^16^. The in vivo assay results concluded that mixtures in straightforward formulations showed an enhanced repellent activity and were statistically significantly comparable with DEET in a period between 1 to 3 h. According to the in vivo assays results reported^16^, and the EAG registers in the current research, mixture 1 (geranyl acetate, α-bisabolol, and nerolidol) was selected to evaluate the differential protein expression. Besides, the DEET synthetic repellent was chosen as a positive control for proteomics experiments.

### Protein identification

A total of 169 protein spots (control vs. mixture 1) were obtained, in which 106 proteins were differentially expressed (up or downregulated). Similarly, the gels obtained for the DEET treatment showed 149 protein spots with 76 differentially expressed proteins (up or downregulated) (Figs. S5–S7).

Through PDQuest analysis of the electrophoresis gels (Fig. S8), for the mixture 1 treatment, 62 proteins were found as upregulated and 44 downregulated in comparison with the control (non-treated mosquitoes). On the other hand, 34 proteins were found as upregulated and 42 downregulated for DEET treatment compared to the control. Some of the upregulated proteins in both electrophoresis experiments were not displayed in the control gels. In other words, this suggests proteins could show differential expression, presumably due to the repellent treatment. In the case of mixture 1, the upregulated proteins listed from 73 to 106 (Table S2) showed no expression in the control electrophoresis gels (non-treated mosquitoes) (Fig. S7). Comparably, the DEET treatment evidenced upregulated proteins not expressed in the electrophoresis control gels, listed from 43 to 76 (Table S3). In Fig. S9, the Venn diagram exhibits the differences in the spots obtained in the gels after comparing mixture 1 vs control and DEET vs control.

Through MALDI-TOF MS, a number of 83 and 70 proteins were identified in the case of mixture 1 and DEET treatment, respectively. Tables S2 and S3 display the identified proteins for each treatment and the fold change and score values obtained. Regarding the protein identification in this research, the proteins listed as No 54, 60, 68, and 76 and proteins No. 20, 34, 39, 45, and 74 (mixture 1), were classified as the same protein. That suggests that in the MS specters analyzed, the same peptide fingerprints were found or likely similar molecular weight of the peptides mentioned. In summary, we identified for the mixture 1, 42 upregulated and 37 down-regulated proteins. In parallel, DEET treatment evidenced 27 upregulated and 37 downregulated identified proteins (Tables S2-S5). In the case of unidentified proteins, probably, the low protein concentration in the gel spot was not enough to be detected through mass spectrometry.

**Figure 6:**
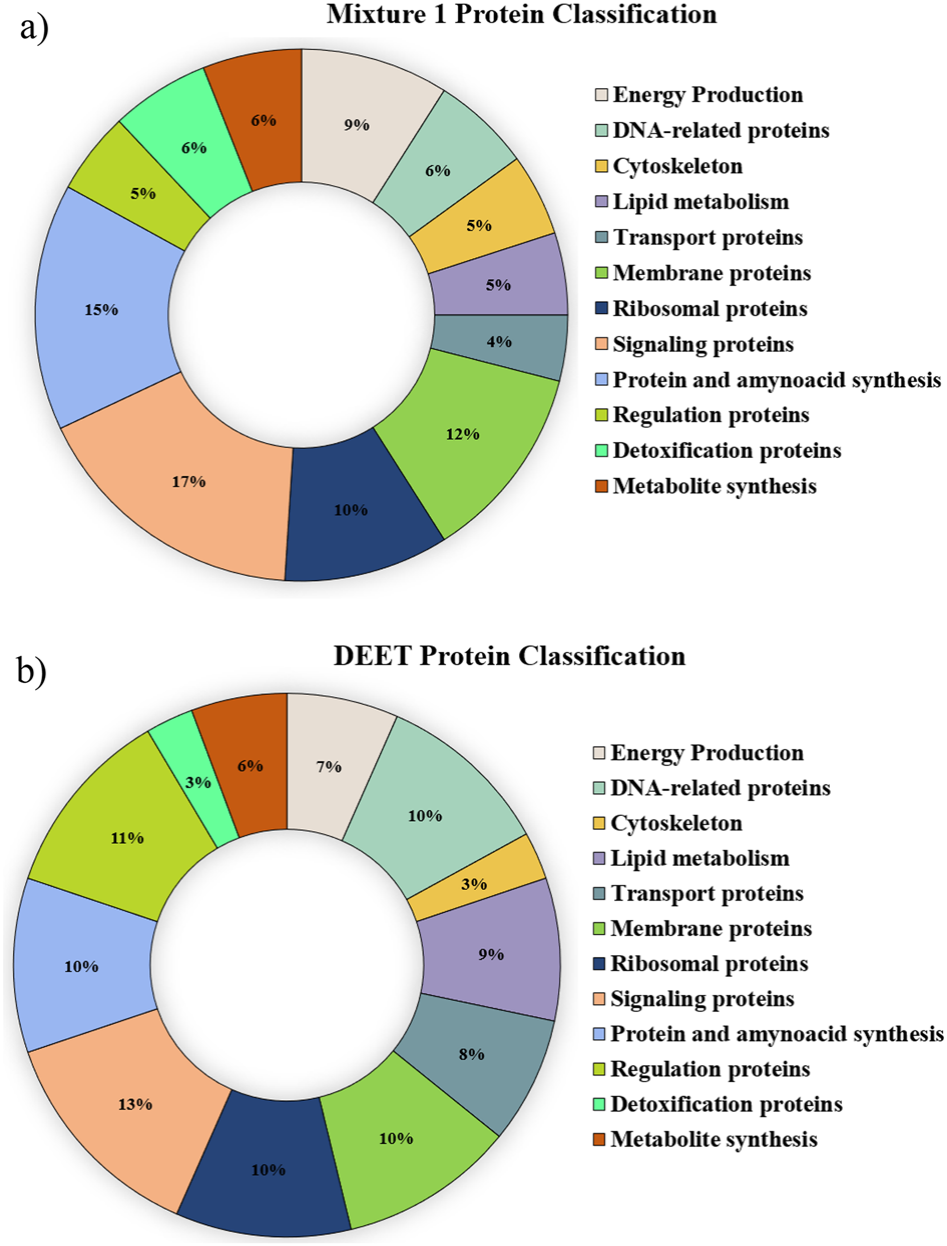
Protein classification according to the cell function. (**a**) Protein classification regarding the mixture 1 (geranyl acetate, α-bisabolol, and nerolidol) at 100 mg/mL. (**b**) Protein classification for the DEET treatment at 100 mg/mL.

The protein peptides identified were related to orthologue proteins existing in different species of bacteria, *Drosophila melanogaster*, mammals, and the *Anopheles gambiae* mosquito. In both treatments, proteins mainly found are related to signaling processes, energy production, metabolite synthesis, transport, detoxification, regulation, lipid metabolism, protein and amino acids synthesis, DNA reparation, nucleotide metabolism, and proteins located in the cell membrane, cell cytoskeletal, ribosomes. In Fig. 6 the proteins displayed in Tables S2 and S3 are classified.

### Protein–protein interaction

**Figure 7:**
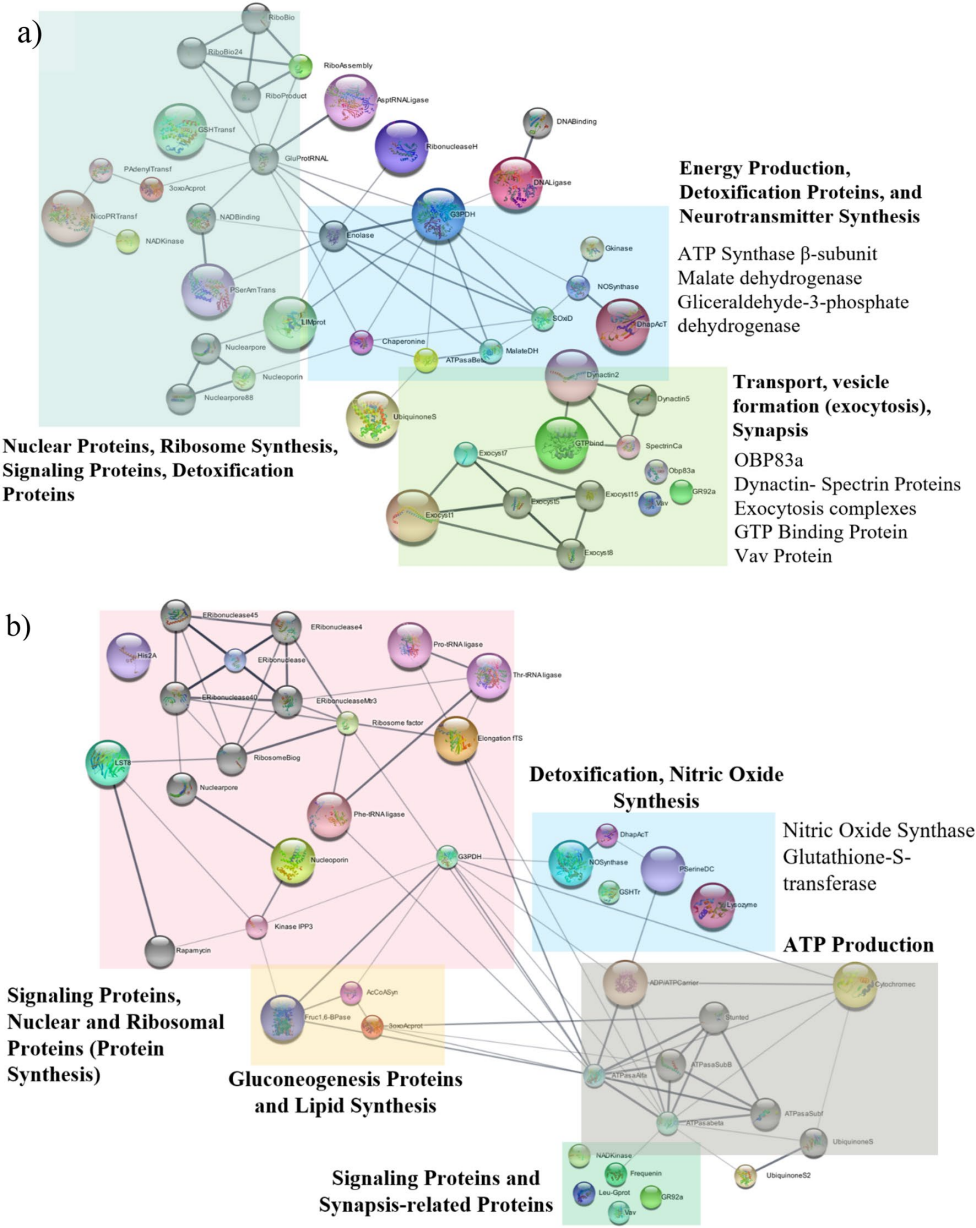
Interactomes obtained by STRING software considering the identified proteins. Reference organism: Drosophila melanogaster. Protein–protein interactions obtained for (**a**) mixture 1 (geranyl acetate, α-bisabolol, and nerolidol) at 100 mg/mL, and (**b**) DEET at 100 mg/mL. The biggest spheres represent over-expressed proteins, the smaller spheres symbolize down-regulated proteins, and gray color spheres represent proteins added by STRING software to find more interactions between protein groups.

Using the online tool STRING, protein–protein interaction diagrams were obtained, considering *Drosophila melanogaster*, as a reference organism. Regarding mixture 1, 28 proteins showed interactions, and 30 proteins for DEET repellent (Fig. 7). Ten interactions were added as a “secondary layer” or secondary interactions. These interactions were added by STRING software to find more interactions with the protein groups obtained above. This improved the interaction analysis since more groups of proteins described different processes performed at the same time. In Fig. 7, the obtained interactomes are displayed for mixture 1 and DEET treatment, considering the identified proteins. Tables S6 and S7 describe the proteins and their corresponding cluster. In the interactome obtained for mixture 1 treatment, it was evidenced that proteins related to transport, vesicle formation, energy production, detoxification, signaling processes exhibit protein interactions. On the other hand, the DEET interactome displayed interactions between proteins related to signaling, synapse, ATP production, and nitric oxide synthesis processes.

## Discussion

### Electroantennography

Natural substances, geranyl acetate, α-bisabolol, and nerolidol, had shown in vivo repellent efficacy and molecular interactions in previous studies on *Ae. aegypti* odorant-binding proteins^16^. The electroantennographic experiments evidenced that exposure to repellents produces electrical signal patterns in different concentrations to DEET and the natural molecules. We evidence that the olfactory neurons containing ion channels and olfactory proteins display an immediate electrical response using the ammonia (NH3) attractant, a key molecule that helps mosquitoes find mammals' location for blood-feeding.

The ammonia signal patterns displayed electrical values between 1 and 4 millivolts (mV). The amplitude of this signal verified the effective electrode connection and molecule perception along with the EAG experiments. In addition to the above, the numeric value related to the EAG signals in the mV scale indicates that ion channels are implicated in the reception process since the ammonia-repellent stimulus the technique detected as a patron electrical signal. According to a previous research^13^, we infer that a greater value in the voltage scale implies more ions passing through neuron membranes, triggering a membrane depolarization. On the other hand, the electrical signals regarding the repellents showed low values on the voltage scale (0–277.8 μV). Nonetheless, this suggests that a low amount of ion channels is activated by these repellent molecules. For instance, the α-bisabolol molecule displayed a 0-mV electrical signal in all cases in the first set of experiments. Thus, it is likely that α-bisabolol perception is related to specific receptors or the molecule exhibits a larger transport route, avoiding receptor or ion channel activation^24^.

### Electroantennographic recordings: repellents and repellent mixtures

The first set of experiments displayed a significant decrease in the EAG signal for almost all the molecules and mixtures implemented. Initially, the electrical signal decrease was attributed to the ample connection time of the electrodes in the mosquito during the experiment. However, after experimenting with only ammonia pulses for 20 min, the signal showed no statistically significant changes in this period (Table S1, Fig. S3). The above confirmed that the ammonia signal decreased due to the repellent exposition. As to the individual EAG assays, the molecules α-bisabolol and nerolidol reduced the post-treatment ammonia signal in a greater proportion than DEET. Nevertheless, we highlighted that a greater decrease in the post-treatment ammonia signal does not necessarily mean an increased in vivo repellent effect. For this, subsequent experiments must be carried out to verify the repellent effect. Although some natural-based repellents displayed greater ammonia signal decrease, in vivo repellent effect is a different variable since natural repellents showed up to 3-h repellency effect and DEET up to 8-h. This variability in repellence suggests that receptors/ion channels are inhibited during repellent exposure, then other processes may take part in the elimination or degradation of repellent molecules^25,26^.

**Figure 8:**
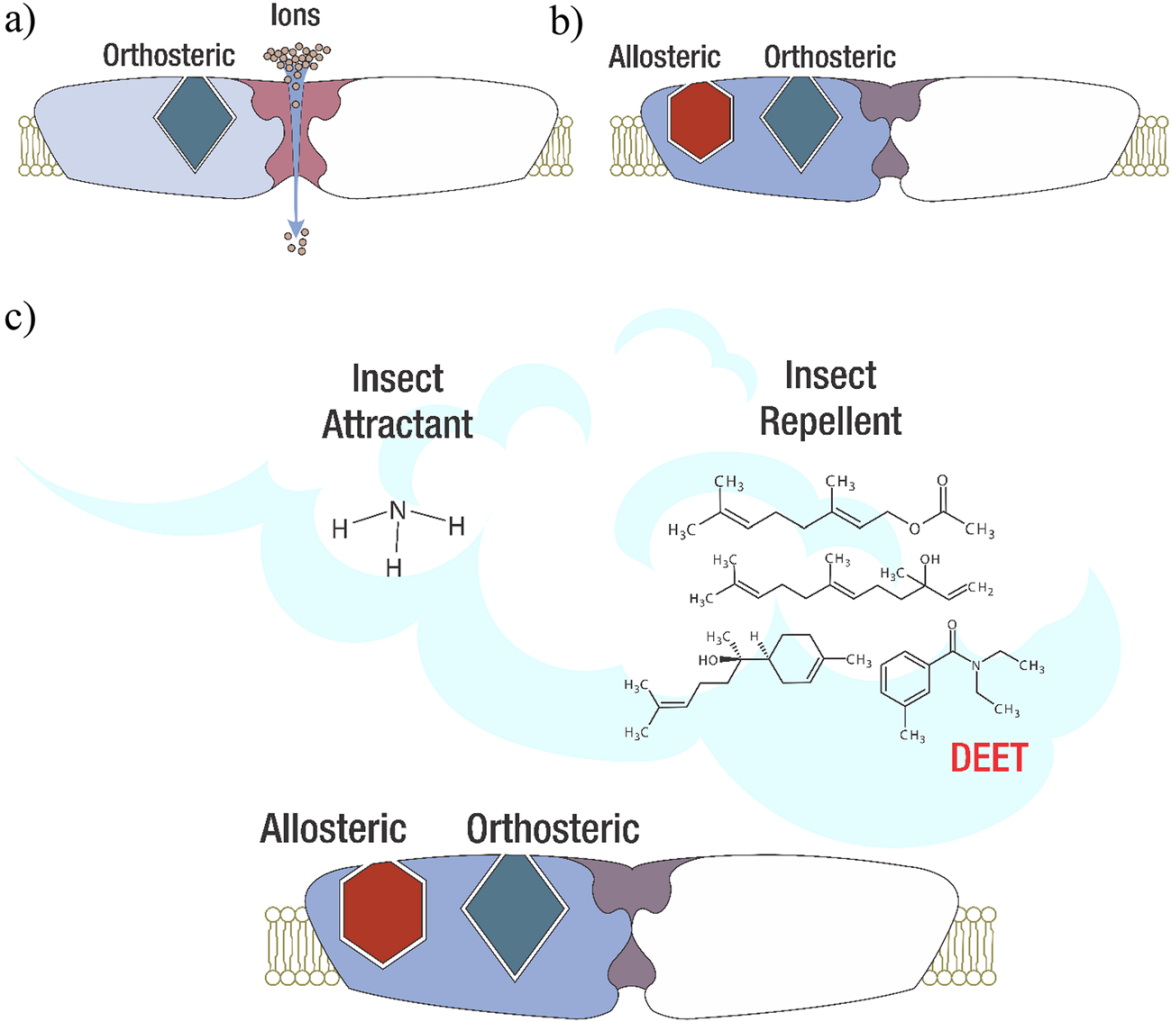
Possible repellent-ionic channel interaction. (**a**) The attractant molecule binds to an orthosteric site that allows ion flux through the cell membrane. (**b**) The same ionic channel possesses different binding sites called allosteric binding sites, in which other molecules bind. In the case of repellents, it is possible that the molecule binds to an allosteric site and inhibits the suitable ionic channel functioning in a small period. (**c**) The masking effect. One of the possible mechanisms for repellents is the repellent-attractant interaction in the liquid phase and vapor phase that prevent molecules from reaching the olfactory system rapidly. Subsequently, the molecule effect in the ionic channel produces the mentioned inhibition. (Adapted by Vidal M. CINTROP from Dickens and Bohbot, 2013).

A hypothesis about odorant perception and its association with ammonia signal decrease is related to protein–ligand interactions. In this sense, the interrelationships are due to ligand interactions with proteins responsible for the transformation of chemical signals into electrical signals such as ionotropic receptors (IRs), odorant receptors (ORs), gustatory receptors (GRs), and ion channels^27–31^. Research has shown that these receptor proteins contain one or several binding sites classified as allosteric or orthosteric. The allosteric site produces a positive or negative effect in the receptor activity with ligand interaction, and orthosteric in which endogenous ligand binds to produce its effects^32–34^. Thereby, ammonia can bind in different receptor-ion channel allosteric binding sites leading to a membrane potential change recorded by the EAG technique. However, with the ammonia post-treatment signal decrease, it is suggested that repellent molecules exhibit competition with ammonia molecules binding to the allosteric sites, which impedes the proper performance of the receptor or ion channel in a determined period (Fig. 8). This effect indicates that the signal decrease recorded in EAG is due to receptor-repellent interactions. However, this kind of competition is not a permanent receptor blockage but a negative modulation leading to a decrease in the number of ions crossing the neuron membrane. The smaller number of ions passing through the membrane, the less ion current and smaller EAG signal is recorded as evidenced in EAG recordings displayed in the results section.

Gene modification in the ion channels (IRs, ORs, or GRs) might confirm the outcomes shown in the current research. For instance, experiments reported^35,36^ carried out modifications in odorant and ionotropic receptors that interact with attractant molecules and other substances like DEET. These experiments showed that structural changes in the receptors also reduce the effectiveness of the molecular interaction with molecules. Therefore, leading to electrophysiological signal reduction and statistically different in comparison with non-modified receptors. In some cases, the modifications performed impede the receptor-molecule interaction with essential substances necessary for mammal host tracking in mosquitoes^37^.

Moreover, some modified receptors evidenced decreased interaction with DEET repellent, leading to a decreased repellent efficacy. Thereby, these experiments suggest that repellent and attractant molecules can bind to one or several receptors and binding sites. In consequence a modification in the receptors might change their sensibility^38,39^. Similarly, the obtained EAG results showed that electrical signals are mainly due to several ion channels or receptor stimulation and not just one single receptor stimulated. However, it is highlighted that the obtained electrical signal displayed low numeric voltage values (mV scale) in the case of repellent molecules. With these results, we infer that the receptors involved are different, and membrane depolarization was not evidenced when compared with the ammonia signal. Besides, DEET-receptor affinity is likely greater than natural-based molecules, allowing a sustained, long-lasting repellent effect^40–42^.

Other research reported^33,43^, focused on ion channel structure, describing the different allosteric binding sites present in the proteins that constitute the mosquito olfactory system. Thereby, several molecules are able to interact with receptors and ion channels producing agonist or inhibitory effects. This mechanism suggests that repellent compounds displayed binding sites bias producing inhibitory effects or reducing the receptor affinity with other compounds momentarily, which leads to a perception reduction. Greater affinity is likely associated with hydrogen bonds or Van der Waals intermolecular forces; as a result, the compound interacts for a more extended period with the receptor-ion channel. Therefore, this might explain the effectiveness of synthetic repellents like DEET since its repellent duration lasts up to 8 h, compared to the natural-based molecules which exhibit 1–3 h. This was evidenced with the post-treatment ammonia electroantennography recordings. Possibly, the repellent molecules interact with receptors decreasing ammonia affinity or perception: As a result, the proper performance of receptors is reduced as well as localization of the host for blood-feeding.

After evaluating every molecule and concentration, it was observed in almost all the cases that greater concentration was applied, a decrease in ammonia post-treatment signal was exhibited. This suggests that a remarkable effect is evidenced in the mosquito olfactory system with a considerable number of compounds applied. For instance, the geranyl acetate showed no statistically significant differences comparing the pre- and post-treatment ammonia signal, notwithstanding, using the same compound in mixtures displayed significant changes in the post-treatment ammonia perception recorded by EAG. In addition to the above, it could be suggested that by implementing the mixtures with 2–3 metabolites, a synergic effect is obtained and confirmed by the in vivo repellence assays. Similarly, DEET repellent applied at 25% v/v in commercial repellents could exhibit synergic effects using the metabolites used in this research, which might be replaced partially by DEET at 0.5–2% using natural-based repellents with synergic effect. Thereby, suggesting an enhanced effect with natural-based and synthetic repellents simultaneous usage. At the same time, mixing DEET with natural repellents allows reducing in a greater proportion the health threats for humans that implies using DEET. Nonetheless, it is highlighted that EAG experiments are not able to predict inhibition, or receptor activity blockage, and more experiments are needed. However, the repellency time of these molecules, previously tested, might show a clue for ion channels performing over a period.

### Electroantennographic recordings: ammonia and repellent simultaneous stimulation

Regarding the molecules implemented in this research, natural-based repellents showed a long-lasting effect between 1 and 3 h. Besides, mixtures 1 and 2 evidenced 3 h as maximum in vivo repellent effect^16,44^. However, comparing the electroantennography and in vivo repellent assays, the in vivo assays represent a real situation due to the volatile compounds present simultaneously in the assay. The mosquitos in the in vivo assays are exposed to attractant and repellent compounds at the same time. This variable was not considered in the first part of electroantennography experiments since ammonia (attractant) and repellent molecules were applied separately. In addition to the above, EAG assays using repellent and attractant simultaneously were carried out to resemble the EAG assays with in vivo experiments.

Concerning ammonia and repellent simultaneous EAG assays, a decrease in the ammonia signal comparing pre- and post-treatment recordings was also obtained. According to that, it is suggested that signal reduction is related to mixture synergic interactions between molecules and ion channels or receptors. Previous researches^36,39^ carried out suggest that the repellent effect is evidenced due to changes in physical-chemistry properties such as vapor pressure producing a masking effect. In other words, the liquid phase interactions decrease the attractant compounds' volatilization. This prevents attractant molecules from approaching the mosquito at the same ratio or in greater amounts (Fig. 10). Other research suggests that repellent and attractant molecules interact in the vapor phase, notwithstanding, it is unlikely since gas molecules evidence weaker interactions due to molecule–molecule distances^45^. With this statement, it is deduced that changes in attractant molecule properties like volatilization, as well as ion channel activity disturbance, are due to interactions caused by repellent substances. Thus, the masking effect is one of the factors that might explain the ammonia signal reduction after applying repellent molecules since the number of molecules reaching the mosquito olfactory system is not the same, in consequence, a decreased amount of ion channels are activated. For further research, it is recommended to carry out experiments related to chemical and tridimensional ion channel and receptor structure. This could explain ligand affinity and molecules able to bind to a specific receptor. Hence, the search for repellent compounds could be improved and combinations of these molecules might enhance repellent effectiveness at low concentrations.

Research reports focused on the olfactory system had described activation pathways related to metabotropic and ionotropic ion channels (ORs, IRs, GRs)^26,27,46^. The ionotropic pathway is associated with direct receptor activation once the volatiles are transported from sensilla lymph to neuron membranes^30,47^. This effect is swift, and the electrical response recorded is immediate. This was evidenced by recording the ammonia signals, in which a high millivolt amplitude value was recorded after ammonia stimulation. In the current research, the repellent molecules showed electrical signals with no more than a few millivolts of amplitude or even null, in the case of α-bisabolol. However, recording the ammonia signal after repellent treatment displayed a decrease in the signal values, likely due to metabotropic pathway participation. The mentioned pathway is slow and dependent on volatile concentration as a wide type of signaling proteins takes part in this process.

Thus, G proteins signaling proteins participate in this process and are responsible for secondary messenger releasing such as cyclic nucleotides (cAMP, cATP, cGMP, cGDP, cGTP, etc.), calcium ions Ca^2+^, inositol triphosphate IPP_3_, among other molecules^7,48,49^. Thereby, interactions between repellents and proteins that lead to signaling cascades are highly likely, producing a particular effect after a short period. In this case, an inhibition or disturbance in ion channel performing. A piece of evidence to the above is related to the first set of EAG assays since an ammonia signal was evidenced as decreased after applying repellent molecules in different concentrations. Because of this, a decrease in the ammonia signal was recorded a few minutes after applying the repellent compounds. Therefore, protein and signaling are likely to take part in this process during repellent exposure. The above might explain the low amplitude values recorded for repellent compounds, since these molecules are able to interact with receptors and signaling proteins, leading to a signaling cascade either in the metabotropic or ionotropic pathway^24^.

The results obtained concerning the repellent mixtures showed the same post-treatment ammonia pattern. Considering the changes in the numeric values in the signals recorded, this decrease in the ammonia signal could be implemented as a screening in search of repellent molecules in future research. Nonetheless, in vivo experiments must be carried out to verify repellent effectiveness against mosquitoes as well as the long-lasting effects on human skin. On the other hand, electrophysiological techniques had been used to observe electrical signals due to repellent compounds^40^. Despite this, no reports focused on signaling reduction after repellent treatment was found. Besides, contrasting with most of the electroantennographic research, most of the studies published to implement the split mosquito head or split antennae^50^. In this research, the mosquito remained alive during EAG experiments. This suggests improved veracity in the recorded signals since the neuronal activity remains unchanged since antennae or heads were not separated from the mosquito’s body. In contrast, after separating antennae or mosquito’s head, insect proteins begin to degrade, and neuronal electrical signals would not be the same.

Concerning the methodology implemented in electroantennography, experiments using repellent molecules against *Anopheles gambiae* reported^40^, described a similar procedure executed in the current research. In the mentioned study, natural-based and synthetic repellents were applied individually and simultaneously with different odorants that the mosquito uses to locate mammals. Similarly, it had been observed that decreased electrophysiological signals suggest a possible masking effect due to repellent perception^40^. In addition to the above, a possible receptor inhibition was also proposed. Despite natural-based and synthetic repellent masking effect evidence, no reports regarding involved proteins involved in odorant reception were shown. In this study, electroantennography assays were followed by protein separation and identification regarding the olfactory mosquito system. The proteomic studies are key to understanding the action mechanism and reception of odorant molecules, attractants, and repellents.

### Proteomics: protein expression and identification

The bidimensional gels obtained were reproducible and showed a considerable amount of protein spots related to proteins separated in the 3–10 pH range (Figs. S6, S7). This displayed a wide pH and molecular weight range. However, most of the proteins up/downregulated were found in the 11–30 kDa range approximately. In this molecular weight range, odorant-binding proteins, transport, signaling, and regulation proteins had been reported, this suggests that the separated proteins in this range are implied in the repellent reception, and transport processes^51–53^.

Subsequently, for both repellent kinds, through MASCOT, Swissprot, and Uniprot databases, proteins were identified as well as their function. The results displayed peptides similarly with a score confidence medium through high (39–71), linked to proteins from bacteria, *Drosophila melanogaster*, and *Anopheles gambiae*. These score values indicate that some mosquito proteins' fingerprint or protein primary structure had not been reported previously for *Ae. aegypti*. Nevertheless, protein classification and function of detected proteins might be involved in signaling processes in neurons, energy production, detoxification, and regulation processes.

### Differential expression analysis and reception mechanism: DEET vs control

Concerning signaling, regulation, transport, energy production, detoxification functions found in this study, implies that during repellent exposure, different metabolic pathways are involved. Firstly, the interactome diagram for DEET synthetic repellent was analyzed. For this treatment, proteins involved in the energy production such as glyceraldehyde 3-phosphate dehydrogenase, acetyl-CoA synthetase, ubiquinone synthetase, and ATP synthase subunits α and β showed downregulated expression. This indicates that glycolysis and oxidative phosphorylation processes involved in ATP production are altered in comparison with the control experiments. In addition to the above, the enzyme fructose 1,6-bisphosphatase was found as upregulated for this treatment. Therefore, it is confirmed that the glycolysis process is negatively regulated, activating the gluconeogenesis route^7,14,54,55^.

In the neuron, the energy demand is high since the high amounts of energy are necessary for the proper functioning of proteins involved during synapse, secondary messenger activation, and neurotransmitter transport^55^. In connection with energy production, the nitric oxide synthase was found as upregulated. The nitric oxide (NO) molecule is cataloged as a neurotransmitter and secondary messenger with different functions in the neuron. One of these is related to negative regulation of energy and ATP production in the cell^54,56^. This is mainly due to nitrosylation reactions, in which protein and NO react, changing the protein and enzyme activity^54^. This might explain the downregulation of glycolysis proteins such as glyceraldehyde 3-phosphate dehydrogenase, chaperonins, proteins that regulate homeostasis, and secondary messengers releasing like guanylate kinases and guanylate cyclase. In research carried out^57–59^, downregulation or changes in the mentioned proteins due to nitric oxide were previously reported (Table S8). Thereby, the activity and expression of several proteins are decreased as a consequence of nitric oxide-protein reaction (nitrosylation). On the other hand, proteins such as proline, threonine, and phenylalanine aminoacyl tRNA synthetases, ribosomal assembly and elongation factors, homolog protein LST8, all of them important during elongation in protein synthesis were found as upregulated. This may suggest that the cell is overexpressing these proteins in order to synthesize more new proteins that allow it to recover from the effect produced by nitric oxide after the change or inhibit the expression of proteins mentioned^54,60^.

Regarding nitric oxide production, reports had shown an increase in nitric oxide after a cell and mitochondrial metabolic stress response^54^. This might be involved in the expression of proteins like cytochrome c, and ADP/ATP translocase in the current study. The mentioned proteins perform the exchange of ADP/ATP between the mitochondria and the cytoplasm as well as are involved in signaling processes concerning apoptosis, programmed cell death. Moreover, it has been reported that cell stress caused by nitric oxide disturbs the cell cycle and nuclear protein functioning^56^. This was evidenced with nuclear proteins downregulated like exoribonucleases and nucleoporins, important during transport, RNA degradation, and nucleotide recycling, before and after the protein synthesis process^61,62^. Hence, the DEET exposure might produce apoptosis and neurotoxicity due to protein inhibition and upregulation of apoptosis factors like cytochrome c in endothelial, neurons, and hepatocyte cells^63,64^.

Another function in respect of nitric oxide is related to retrograde neurotransmitters^56,65^. This description is attributed to neurotransmitters synthesized in the postsynaptic neuron and transported towards presynaptic neurons, producing a cyclic effect in signaling transmission. As a result, the information flux towards postsynaptic neurons is delayed. Therefore, this indicates that DEET synthetic repellent's long-lasting effect is due to the previous process explained. Once the DEET molecule is inside the olfactory system, the neurons start to produce a high amount of nitric oxide. In consequence, other metabolic processes like energy production, signaling, and detoxification slower their activity. This was evidenced since proteins related to signaling and regulation were found as downregulated.

**Figure 9:**
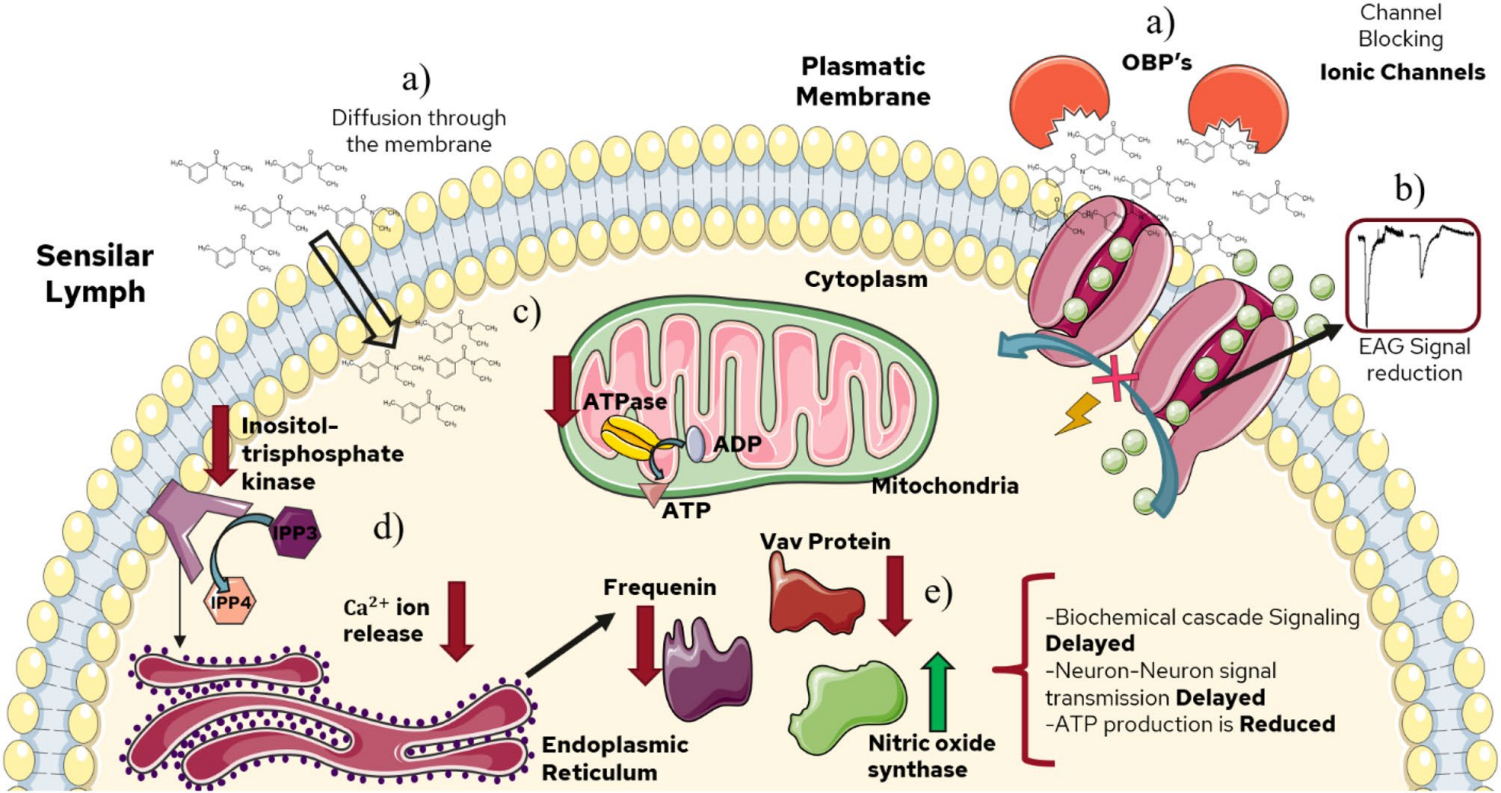
Possible action/reception mechanism for DEET repellent treatment. (**a**) The volatile molecules are transported to the ionic channels by odorant-binding proteins (OBP’s) or are diffused into the cell membrane. (**b**) The interaction DEET-ionic channel decreases the ionic channel activity, thus, reducing the electrical signal. (**c**) DEET repellent inhibits the ATPase protein expression, important to produce ATP in the mitochondria. (**d**) The entry of DEET into the olfactory system alters the protein expression needed to release Ca^2+^ ions, therefore, inhibiting the proper functioning of other related processes. (**e**) Other downregulated and over-expressed proteins trigger delays in signaling cascades and chemical signaling transmission.

In addition to the above, inositol trisphosphate kinase involved in the inositol phosphorylation producing inositol trisphosphate (IPP_3_) and phosphorylated derivatives was found as downregulated. The mentioned phosphorylated derivatives are involved in intracellular calcium ion releasing (Ca^2+^)^49^. In consequence, the disturbance in the activity or expression of this enzyme leads to homeostasis deceleration and delay, therefore, causing cellular stress due to either excess or lack of Ca^2+^ ions (Fig. 9). Similarly, frequenine-1, a calcium ion-dependent protein was found as downregulated. This protein is relevant for regulation during synapse processes^59,66^. One of its functions is performed as a Ca^2+^ sensor producing a signaling cascade that allows the proper functioning of kinases, important proteins involved in monophosphate nucleotide synthesis (cGMP, cGDP, cGTP, cAMP, GMP, GDP, GTP, AMP, etc.). The enzymes guanylate kinase, NAD kinase, domain protein LIM, lin-7 homolog B, and AMP-GMP cycle synthase (Table S2) were some of the kinase and transport proteins involved in the process described that were found as downregulated in this research. Thereby, proteins downregulated such as frequenine-1, guanylate kinase, and NAD kinase, change the releasing and production of secondary messengers involved in chemical-electrical signaling transduction in the metabotropic pathway^24,66^. Therefore, the downregulation of the mentioned proteins leads to a delay in odorant reception, and neuronal signaling transmission.

On the other hand, in the interactome diagram proteins with no interactions were also found. Several of these proteins, like Vav protein, leucine-rich G protein, odorant-binding protein 83a, and gustatory receptor 92 are involved in signaling processes^29,59,67,68^. For instance, the Vav protein plays a vital role in coupling tyrosine kinases that trigger GTPase activity and signaling cascades for signaling transduction in neurons^67^. Moreover, leucine-rich G protein is necessary for protein–protein interactions related to signal cascade activation during molecule reception in the olfactory system^69,70^. Similarly, the gustatory receptor 92 (GR92a) was downregulated, suggesting that volatile reception activity is likely to be reduced. As reported, most of the gustatory receptors are mainly involved in the carbon dioxide reception, a key molecule for blood-feeding search in mosquitos^34,71^. Thereby, in contrast with the obtained results in electroantennography and in vivo experiments, a possible reception or action mechanism for synthetic repellents is the ATP and secondary messengers production disturbance and inhibition of proteins involved in information transmission during neuronal synapses. In consequence, the neuron-neuron signal transmission occurs more slowly until DEET is eliminated or degraded by the organism.

### Differential expression analysis and reception mechanism: mixture 1 vs control

Concerning the mixture 1 interactions diagram, differences in some clusters comparing mixture vs DEET are displayed. One of these differences is related to the production and transport of vesicles involved in neurotransmitter transport. This group is conformed by proteins like dynactin proteins, spectrin, the GTP union factor, and the exocytosis complexes Exo70 and Sec3. Additionally, the dynactin, GTP union factor, and the ephrine-1 (Table S4) were found as upregulated. Whereas vesicle-membrane union proteins such as spectrin, the exocytosis (Exocyst) complexes, domain protein LIM, and lin-7 homolog B (Table S4) were found as downregulated. The dynactin proteins are found along the neuron axon. The upregulation of these proteins suggests that the neuron increases the transport activity before mixture 1 repellent exposure. Similarly, this effect is related to other upregulated proteins like LIM Mlp84b that allow the cytoskeletal integrity and regulate the cell mobility effectiveness with actin proteins assistance^55^.

In this way, though vesicle-membrane binding spectrin proteins were found to be downregulated, the neuron increases the transport effectiveness to counteract the inhibition and continue the transport and signaling transduction processes. Consequently, once the mosquito is exposed to mixture 1, vesicle and neurotransmitter transport are altered. Thus, the synapse process is affected and delayed. This could be corroborated since enzymes like guanylate kinase, NAD kinase, Vav protein, guanine-binding protein, homolog lin-7 protein, AMP-GMP cycle synthase, were also found as downregulated. All the above-mentioned proteins are implied in the secondary messengers released responsible for triggering GTPase activity and signaling cascades for signal transduction. Therefore, the transport and synapse processes induce slower chemical to electrical signaling transduction. This effect mentioned is similar to the results found through electroantennography since natural-based molecules decreased the electrical signal regarding the ammonia pulse, directly related to ion channels and synapse process.

In the case of mixture 1, proteins related to oxidative stress such as nitric oxide synthase, superoxide dismutase, and the chaperonin 60 kDa protein were found as downregulated, contrary to DEET treatment. Research reported previously found that superoxide dismutase and chaperonin proteins are co-expressed in order to avoid protein damage during oxidative stress^57^. The downregulation of this kind of protein suggests that the mixture 1 repellent triggers chemical reactions that inhibit the expression of the above-mentioned proteins. Therefore, the repellent odorant reception did not produce an oxidative stress response. However, detoxification proteins like Aldo–keto reductase, glutathione-S-transferase, glutaredoxin, and nicotinate phosphoribosyltransferase displayed upregulation^54,72,73^. These proteins easily react with molecules containing ketone, ester, aldehydes, electrophilic carbon groups, present in geranyl acetate, α-bisabolol, and nerolidol repellent molecules^74^ (Fig. S10).

Although oxidative stress was not exhibited, the cell eliminates the repellent mixture molecules using detoxification proteins, a more effective pathway due to the chemical bonds present in the natural-based repellents. This indicates that the low repellent effectiveness of natural-based repellents is highly related to elimination pathways such as detoxification proteins, an event not displayed for DEET synthetic repellent. Similarly, with DEET treatment, energy production proteins like ATP synthase β-subunit, malate dehydrogenase, and ubiquinone synthase showed downregulation. In this case, the energy production alteration reduces the activity of other ATP-dependent processes. This might be related to neurotransmitter transport proteins' down-regulation since a high demand for ATP is needed for proper performance.

This study highlights that *Ae. aegypti* differential proteomics exposing the mosquito to repellent compounds had not been reported. Some research published had extracted *Ae. aegypti* proteins, however, the proteins found were not the same since the reports focused on different objectives^74–76^. Moreover, considering the electroantennography it can be confirmed that synthetic repellents displayed a different action mechanism in comparison with natural-based compounds.

**Figure 10:**
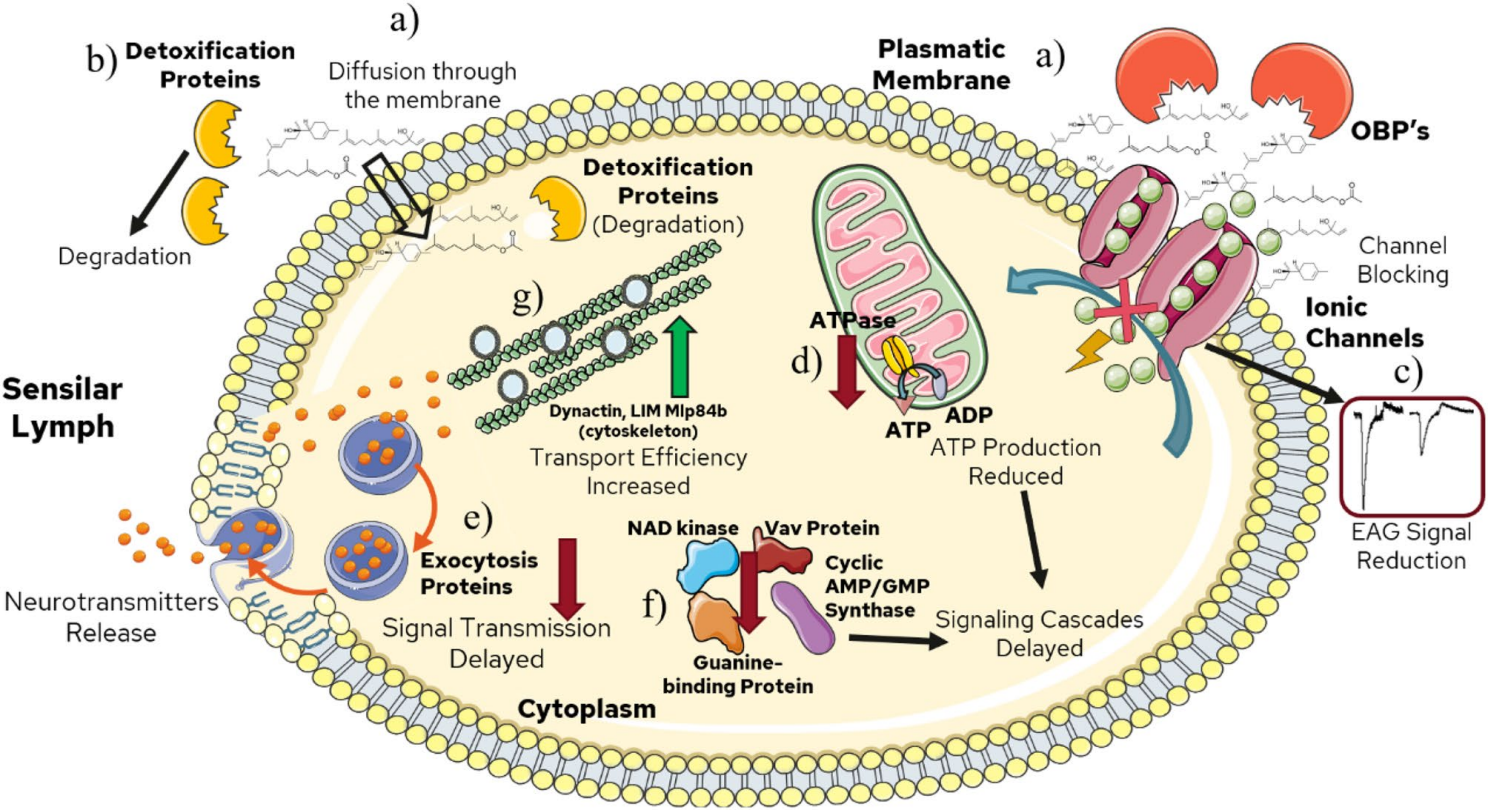
Possible action/reception mechanism for mixture 1 treatment. (a) The volatile molecules are transported to the ionic channels by odorant-binding proteins (OBP’s) or are diffused into the cell membrane. (b) Detoxification proteins degrade the terpenoid compounds. (c) The mixture-ionic channel interaction decreases the channel activity, therefore, reducing the electrical neuronal signal. (d) mixture 1 inhibits the ATPase protein expression, important to synthesize ATP in the mitochondria. (e) The proteins related to the exocytosis process are downregulated due to the treatment, delaying the synapse process. (f) Other inhibit proteins delay the signaling process in the cell. (g) The dynactin protein overexpression increases the vesicle transport efficacy, necessary for the neurotransmitter signaling transmission process.

Concerning the reception of repellent compounds, it is likely that either synthetic or natural-based based molecules exhibit almost the same reception mechanism through odorant-binding proteins. However, the mosquito response is different for both repellents. In the case of DEET, synapse efficacy and energy production (ATP) processes showed alterations, reducing the electroantennographic signal due to neuronal activity decrease. Besides, the DEET treatment showed no upregulation of detoxification proteins. Therefore, it is suggested that DEET displays enhanced and prolonged interaction with ion channels and receptors compared to natural-based repellents (Fig. 10).

In contrast, mixture 1 showed downregulation of proteins responsible for processes such as ATP synthesis (energy), signaling, vesicle, and neurotransmitter transport leading to negative alteration of synapse. Despite the mentioned effect, in response to natural-based repellent exposition, the cell overexpressed detoxification proteins, as a result, degradation of these molecules is carried out. In both treatments, disturbances in neuronal synapses were found, which might be related to the decrease in the EAG signal reported in this research. Finally, it highlights the application of electroantennography and proteomics to elucidate the masking effect and the electrical signal alterations perceived by the olfactory system described previously^40^. In addition, the proteomic contributions focused on the identification of mosquito olfactory system proteins, necessary in the molecular reception of repellent compounds. In conjunction, the current research represents an approach to possible action and reception mechanisms regarding synthetic and natural-based repellents against *Aedes aegypti* mosquitoes. Further research might perform the search and design of repellent molecules with longer long-lasting effects considering the protein effects and electrophysiological signals found in this study. Hence, harmful synthetic repellents like DEET and IR3535 might be replaced partially or totally by natural-based compounds with no secondary effects, leading to enhanced repellent efficacy and safety for human beings.

## Conclusions

The results found with electroantennography, and proteomic methodologies showed that the repellent compound-olfactory system interaction disrupts ionic channels. Besides, the presence of these repellent molecules in the mosquito olfactory system led to down-regulated protein expression of essential proteins necessary for synapse, energy production, disruption of transport processes, and vesicle formation. Natural-based repellents' molecular effectivity upregulates detoxification proteins needed for degradation; therefore, the molecules are easily removed from the olfactory mosquito system. On the other hand, DEET showed no upregulated expression of detoxification proteins, which suggests greater repellent efficacy as previously reported with in vivo assays.

The electrical signaling patterns obtained are suggested to work as a screening process for repellent molecules. The ammonia signal patterns change due to repellent exposition, this effect is directly related to ionic channels activity in the olfactory system. However, it is highlighted that in vivo assays are necessary to confirm repellent efficacy and long-lasting effect.

Protein–protein interaction diagrams demonstrated that DEET and natural-based repellents exhibit different reception/action mechanisms. In the case of DEET, synaptic processes, energy production, and neurotransmitter biosynthesis like nitric oxide were disrupted. On the other hand, vesicle transport, signaling processes important for synapse, and energy production were disrupted when the mosquitoes were exposed to mixture 1 repellent.

## Supplementary Information

Supplementary Information.

## Data Availability

The datasets generated and/or analyzed during the current study are available in figshare repository: 10.6084/m9.figshare.20514888.v1.
