# Supplementary material for: Differentiation of action mechanisms between natural and synthetic repellents through neuronal electroantennogram and proteomic in Aedes aegypti (Diptera: Culicidae)
Source: Sci Rep. 2022 Nov 27;12:20397. doi: 10.1038/s41598-022-24923-x (PMC9701785; doi:10.1038/s41598-022-24923-x)

**Supplementary material**

Differentiation of action mechanisms between natural and synthetic repellents through neuronal electroantennogram and proteomic in *Aedes aegypti* (Diptera: Culicidae)

Johan Sebastián Portilla Pulido^1, 3^, Diana Lizeth Urbina Duitama^1, 3^, María Carolina Velasquez Martinez^2^, Stelia Carolina Mendez-Sanchez^1^, Jonny Edward Duque^3*^.

1. Grupo de Investigación en Bioquímica y Microbiología (GIBIM), Facultad de ciencias, Escuela de Química, Universidad Industrial de Santander. E-mail: [scmendez@uis.edu.co](mailto:scmendez@uis.edu.co)
2. Grupo de Investigación en Neurociencias y Comportamiento UIS-UPB. Facultad de Salud, Escuela de Medicina, Departamento de Ciencias Básicas Universidad Industrial de Santander, Bucaramanga, Santander, Colombia. [macarvel@uis.edu.co](mailto:macarvel@uis.edu.co)
3. Centro de Investigaciones en Enfermedades Tropicales - CINTROP. Facultad de Salud, Escuela de Medicina, Departamento de Ciencias Básicas Universidad Industrial de Santander, Bucaramanga, Santander, Colombia. [jonedulu@uis.edu.co *correspondence](about:blank) author

**Supplementary Figure 1.**

Electroantennographic signal patterns and percentage changes in the post-treatment ammonia signal after applying the geranyl acetate in 0.5, 1.0, 10, 50, and 100 mg/mL. For the experiment, an initial and final pulse with ammonia was applied. This repellent showed no statistically significant differences comparing the EAG ammonia signals pre- and post-treatment. (ANOVA F(6,42)=16.19, p >0.05).


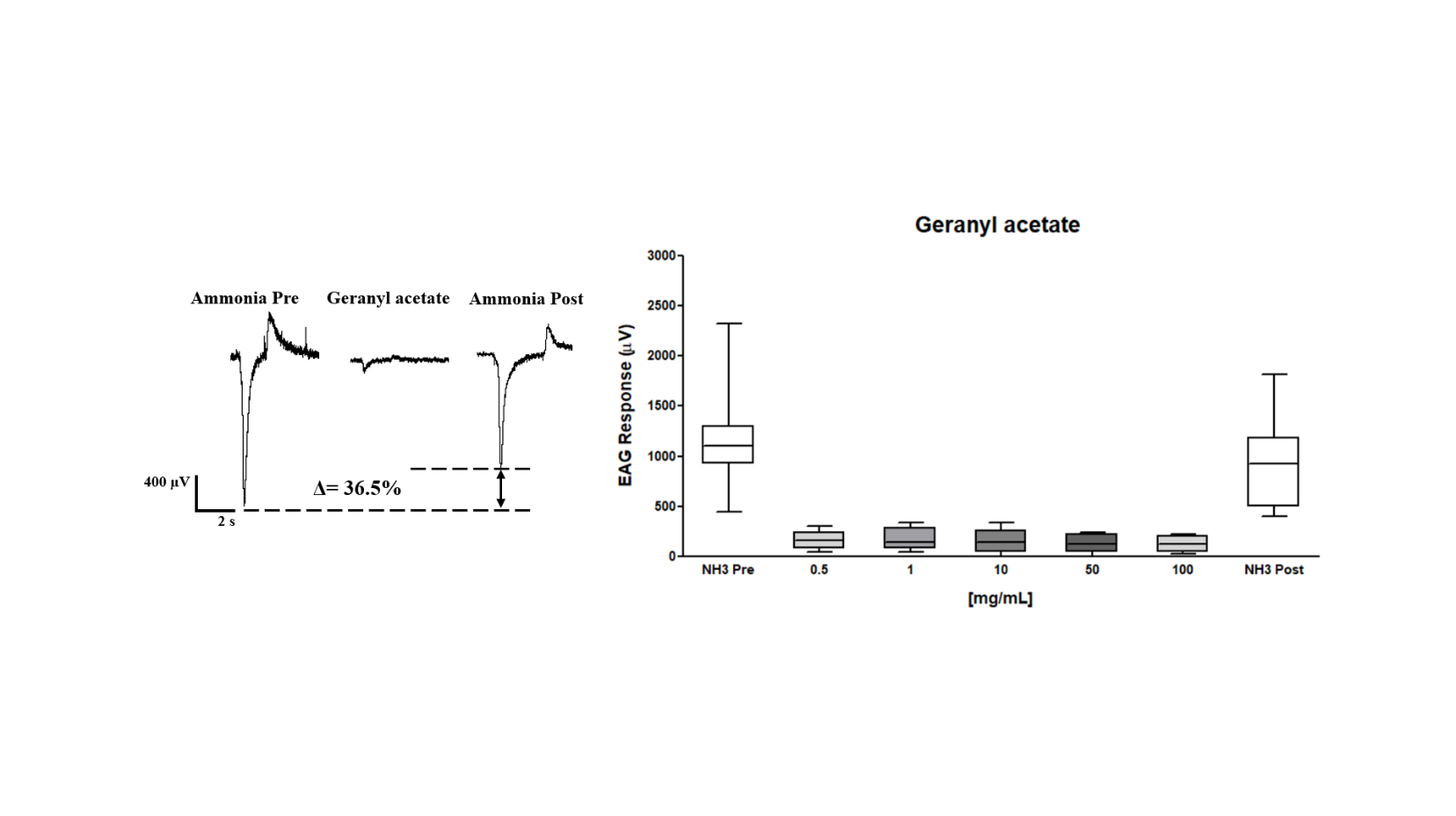


**Supplementary Figure 2.**

Electroantennographic signal patterns and percentage changes in the ammonia EAG signal applying different repellent mixtures. a) Mixture 1 (geranyl acetate, α-bisabolol, and nerolidol) [50 mg/mL], b) mixture 2 (geranyl acetate and nerolidol) [50 mg/mL], c) mixture 2 [100 mg/mL], d) mixture 3 [50 mg/mL], e) mixture 3 (nerolidol and α-bisabolol) [100 mg/mL], f) mixture 4 (geranyl acetate and  α-bisabolol) [50 mg/mL]. For each experiment an initial and final pulse with ammonia was applied. (*), (**), and (***) indicate statistically significant differences comparing the pre- and post-treatment ammonia signal. (*) p < 0.05, (**) p < 0.01, (***) p < 0.001, Tukey test. Statistic values: mixture 1 [50 mg/mL] (ANOVA F(2,18)=154.8, p <0.0001), mixture 2 [50 mg/mL] (One way ANOVA F(2,24)=130.1, p<0.0001), mixture 2 [100 mg/mL] (One way ANOVA F(2,18)=43.22, p <0.0001), mixture 3 [50 mg/mL] (One way ANOVA F(2,18)=39.18, p <0.0001), mixture 3 [100 mg/mL] (One way ANOVA F(2,18)=30.31, p <0,0001), and mixture 4 [50 mg/mL] (ANOVA F(2,21)=25.06, p <0.0001).








### ***Ammonia reduction signal verification***

Contrasting the EAG experiments carried out with different concentrations (0.5, 1.0, 10, 50, and 100 mg/mL) and with concentrations of 50 and 100 mg/mL, no statistically significant differences were evidenced comparing the changes in the EAG signals related to the ammonia using a single mosquito for all the concentrations (0.5, 1.0, 10, 50, and 100 mg/mL) in comparison with testing 2 concentrations (50 and 100 mg/mL) in a mosquito. This comparison was expected since the electrical signal regarding the ammonia (pre- and post-treatment) and the treatment (repellents) exhibits similar numerical values as well as percentual reduction of the ammonia post-treatment signal (Figures 3-5). Therefore, in the following experiments carried out, a single mosquito per concentration and substance was used with the same number of replicates as mentioned in the methodology (N=7). To verify that the ammonia signal reduction obtained was not related to the duration of the experiment but to the repellent molecules applied in the air flux, EAG recordings with only ammonia were carried out in different periods (0, 10, 15, and 20 minutes).

As it is evidenced in Supplementary Figure 3, periods 0, 10, 15, and 20 minutes, showed no statistically significant differences comparing the amplitude in each period mentioned. This confirmed that the EAG ammonia signal obtained in the experiments is due to the repellent treatment. The results were hypothesized since other factors such as additional electromagnetic signals, insect connection, and survival during the experiment were considered beforehand. The values for EAG signals regarding the ammonia in different periods are displayed in Supplementary Table 1.

**Supplementary Table 1.**

Electroantennography signals (microvolts ± standard deviation, µV ± SEM) regarding the ammonia electrical signal at different times: initial (0 minutes), 10, 15, and 20 minutes. The ammonia electrical changes showed no significant differences along the periods of exposition mentioned (0-20 min).

|  | EAG Response (µV ± SEM) | | | |
| --- | --- | --- | --- | --- |
| Molecule/Time | **Initial (0 min)** | **10 min** | **15 min** | **20 min** |
| NH_3_ | 3,563.0 ± 380.4 | 2,655.0 ± 464.1 | 2,195.0 ± 299.9 | 2,315.0 ± 306.5 |

**Supplementary Figure 3.**

Electroantennographic signaling patterns related to the ammonia electrical signals in different times 0, 10, 15, and 20 minutes. In the graphics, the signaling recordings are shown. The recordings showed no statistically significant changes along the periods of exposition (p > 0.05, Tukey test).

*
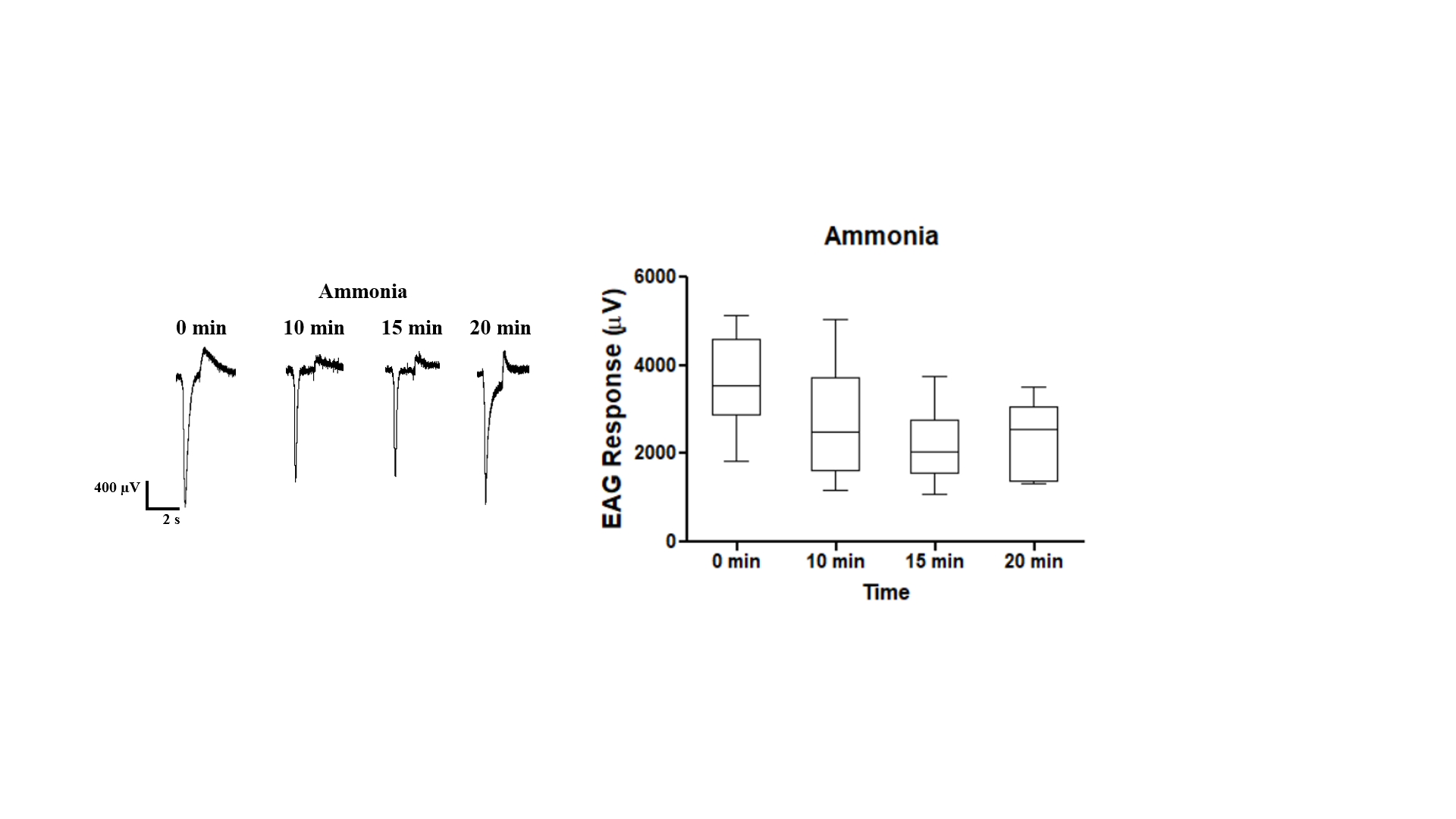
*

**Supplementary Figure 4.**

Electroantennographic signal patterns and percentual changes in the EAG ammonia post-treatment signal applying repellent and ammonia (attractant) simultaneously. a) Geranyl acetate 50 mg/mL, b) geranyl acetate 100 mg/mL, c) α-bisabolol 50 mg/mL, d) nerolidol 50 mg/mL, e) DEET 50 mg/mL, f) mixture 1 [50 mg/mL], g) mixture 2 [50 mg/mL], h) mixture 2 [100 mg/mL], i) mixture 3 [50 mg/mL], j) mixture 3 [100 mg/mL], k) mixture 4 [50 mg/mL], l) mixture 4 [100 mg/mL]. The ammonia stimulus pulse was followed by a repellent-ammonia pulse in order to compare the percentage changes in the electrical signal. (*) Indicates statistically significant differences between the initial ammonia signal and the ammonia-repellent electrical signal (t-student test). Statistical values: Geranyl acetate 100 mg/mL (One way ANOVA, t-test, t(8)=3.189; p= 0.0128), nerolidol 50 mg/mL (One way ANOVA, t test, t(6)=3.794 p=0.0090), nerolidol 100 mg/mL (One way ANOVA, t test, t(7)=5.129; p=0.0014), DEET 100 mg/mL (One way ANOVA, t test, t(7)=2.883; p=0.0236), mixture 2 [100 mg/mL] (One way ANOVA, t test, t(6)=3.935; p=0.0077), mixture 3 [50 mg/mL] (One way ANOVA, t test, t(6)=2.791; p=0.0315), mixture 3 [100 mg/mL] (One way ANOVA, t test, t(6)=3.912; p=0.0079), mixture 4 [50 mg/mL] (One way ANOVA, t test, t(6)=3.268; p=0.0171), mixture 4 [100 mg/mL] (One way ANOVA, t test, t(6)=3.295; p=0.0195). Mixture 1 (geranyl acetate, α-bisabolol, and nerolidol), mixture 2 (geranyl acetate and nerolidol), mixture 3 (nerolidol and α-bisabolol), mixture 4 (geranyl acetate and α-bisabolol).














***Complementary results: Proteomics***

*Aedes aegypti* mosquito’s head, a unidimensional SDS gel was carried out to confirm protein separation through SDS gels according to protein molecular weight. In the Supplementary Figure 5, the protein extraction is displayed using 120-140 mosquito heads. As a result, different strips in the 46-203 kDa weight range were found. This experiment calculated approximately 10 mg/mL for protein concentration. In addition to the above, these experiments were followed by bidimensional gel separation using the treatments mentioned in the methodology section, mixture 1 (geranyl acetate, α-bisabolol, and nerolidol) DEET at 100 mg/mL, using as a control non-treated mosquito.

The protein extraction for repellent treatments and control showed a protein concentration between 8 to 13 mg/mL through the Bradford method in all the replicates. The Supplementary Figure 6 the gel images with the analyzed spots for each replicate, non-treated mosquitos, mixture 1, and DEET. The replicates of each treatment and control are shown in the Supplementary Figure 7.

Protein spots with a molecular weight variety range were found Using a pH range between 3 to 10. Considering the treatment by triplicate, the reproducibility is evidenced for protein extraction and separation since almost the same protein spots were found for each replicate (Supplementary Figure 7). Moreover, after a quick review of control vs repellent treatment gels, it is noticed that protein differences are found as more spots in the repellent treatment gels were separated in comparison with the control gels. The most numerous protein spots were found in the 11-25 kDa molecular weight range.

Analyzing the bidimensional gels through PDQuest software, the master gels representing the triplicate union or summary were obtained (Supplementary Figure 8). In these master gels, the total amount of protein spots for each experiment is displayed. In control vs mixture 1 comparison, a total of 169 spots were found, in which 106 were differentially expressed (upregulated or downregulated). Similarly, comparing control vs DEET treatment, 149 protein spots were observed, and 76 differentially expressed (upregulated or downregulated).

**Supplementary Figure 5.**

SDS page electrophoresis gel of female *Ae. aegypti* heads sample. In this electrophoresis gel, different molecular weight strips are shown. Molecular weight range: 46-203 kDa.


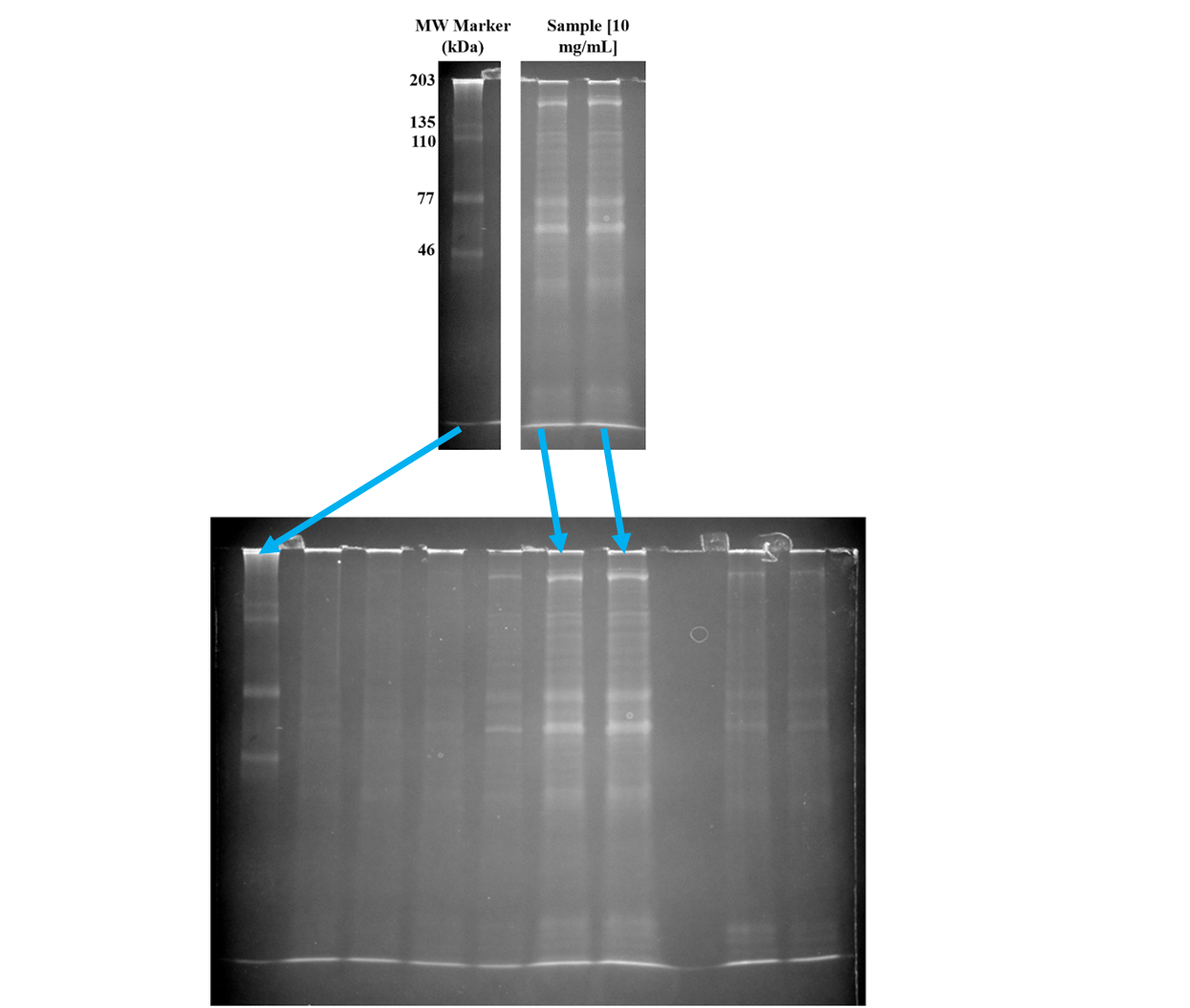


**Supplementary Figure 6.**

Bidimensional electrophoresis gels regarding the proteins of female *Ae. aegypti* heads obtained. a) Control, non-treated mosquitoes, the numbers shown correspond to identified proteins in the control vs mixture 1 treatment. b) Original image of Control. c) Mixture 1 (geranyl acetate, α-bisabolol, and nerolidol, 100 mg/mL each) treatment. d) Original image of Mixture 1. e) Control, non-treated mosquitoes, the numbers shown correspond to identified proteins regarding the DEET repellent treatment. f) DEET repellent treated mosquitoes at 100 mg/mL. In the gels, the differential spots are listed and shown in red, each spot number can be searched in the Supplementary Tables 2 and 3 for the mixture 1 or DEET treatment, respectively.



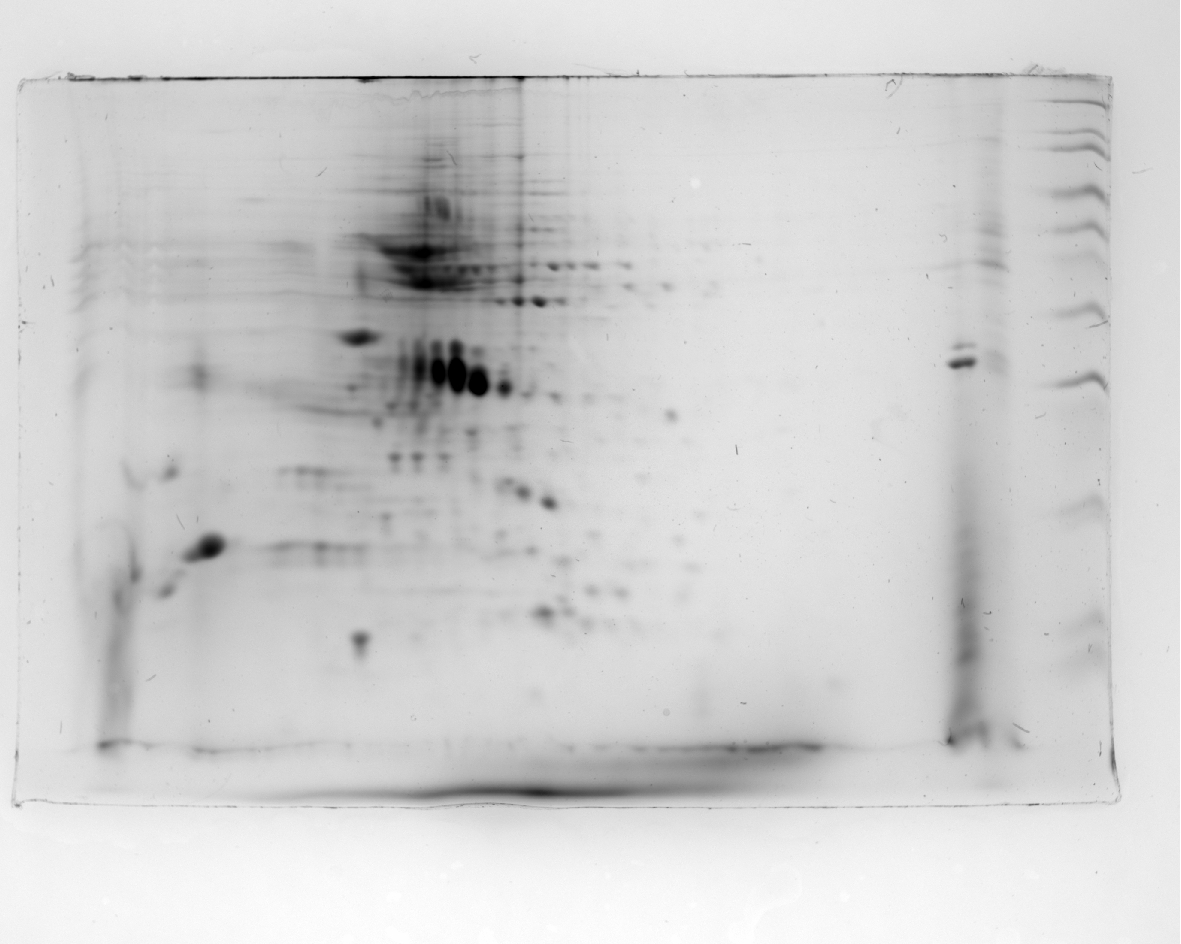



d)


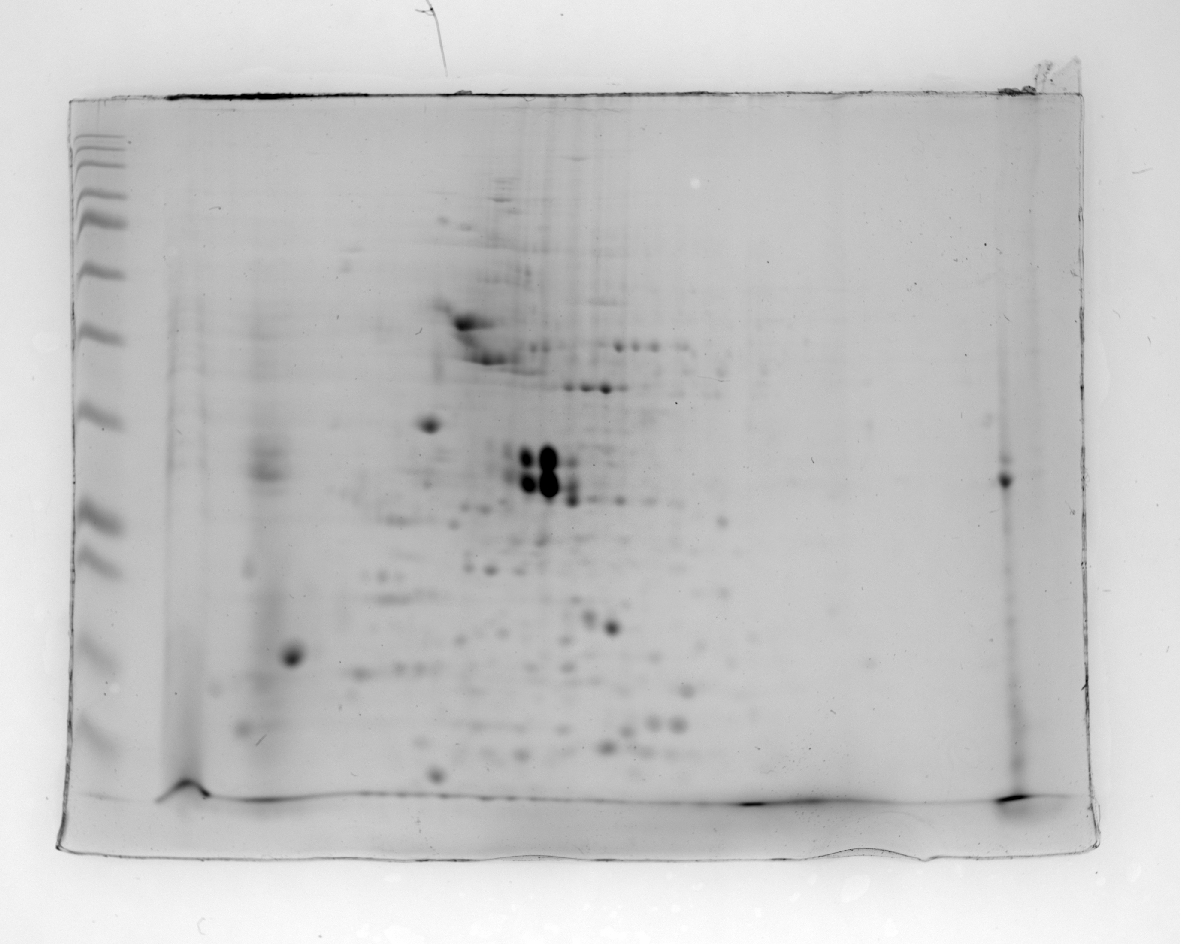



f)


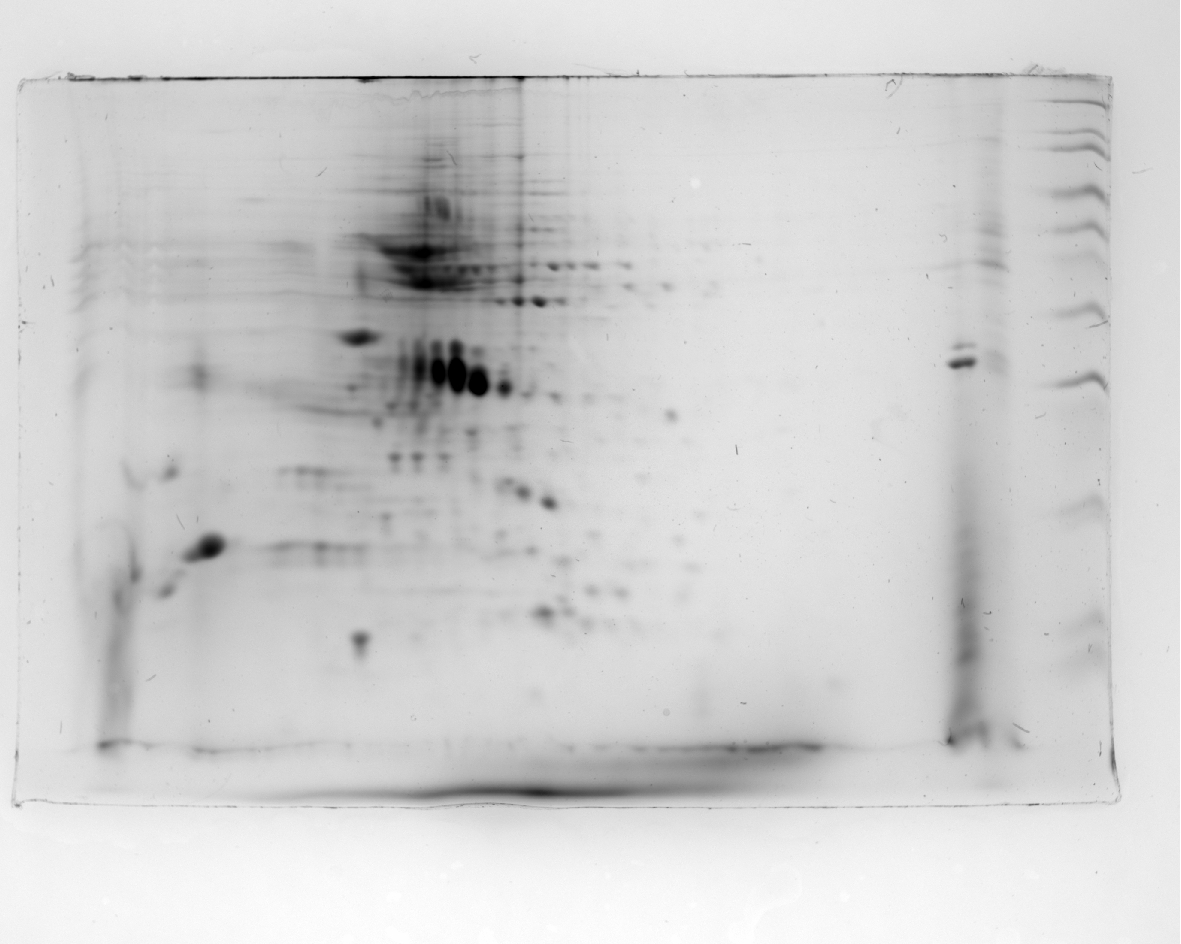


**Supplementary Figure 7.**

Bidimensional electrophoresis gels replicate. a to c) Triplicate corresponding to the control, non-treated mosquitoes, with their respective original images. d to f) Triplicate regarding the mixture 1 (geranyl acetate, α-bisabolol, and nerolidol) treatment at 100 mg/mL. g to i) Triplicate corresponding to the DEET repellent treatment at 100 mg/mL.

a)


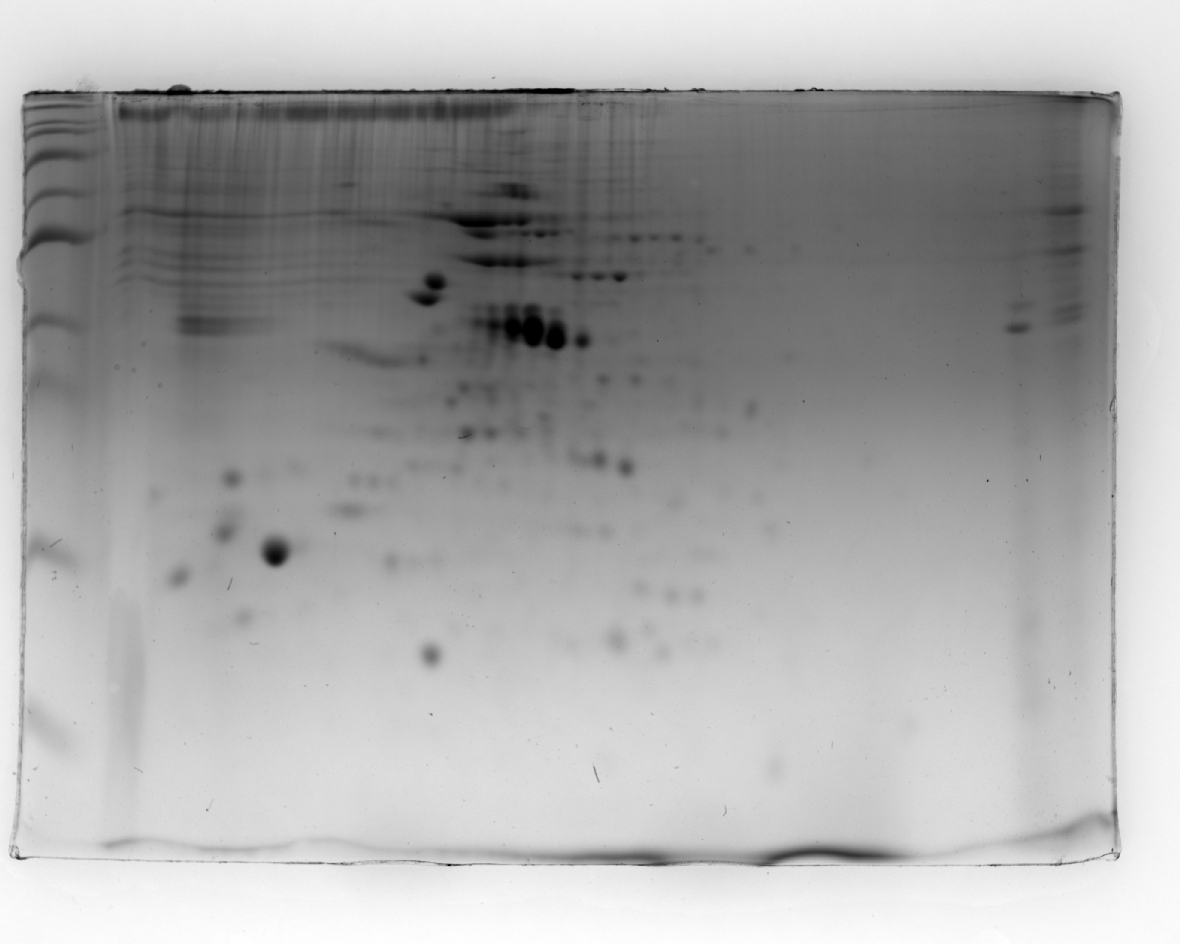


b)


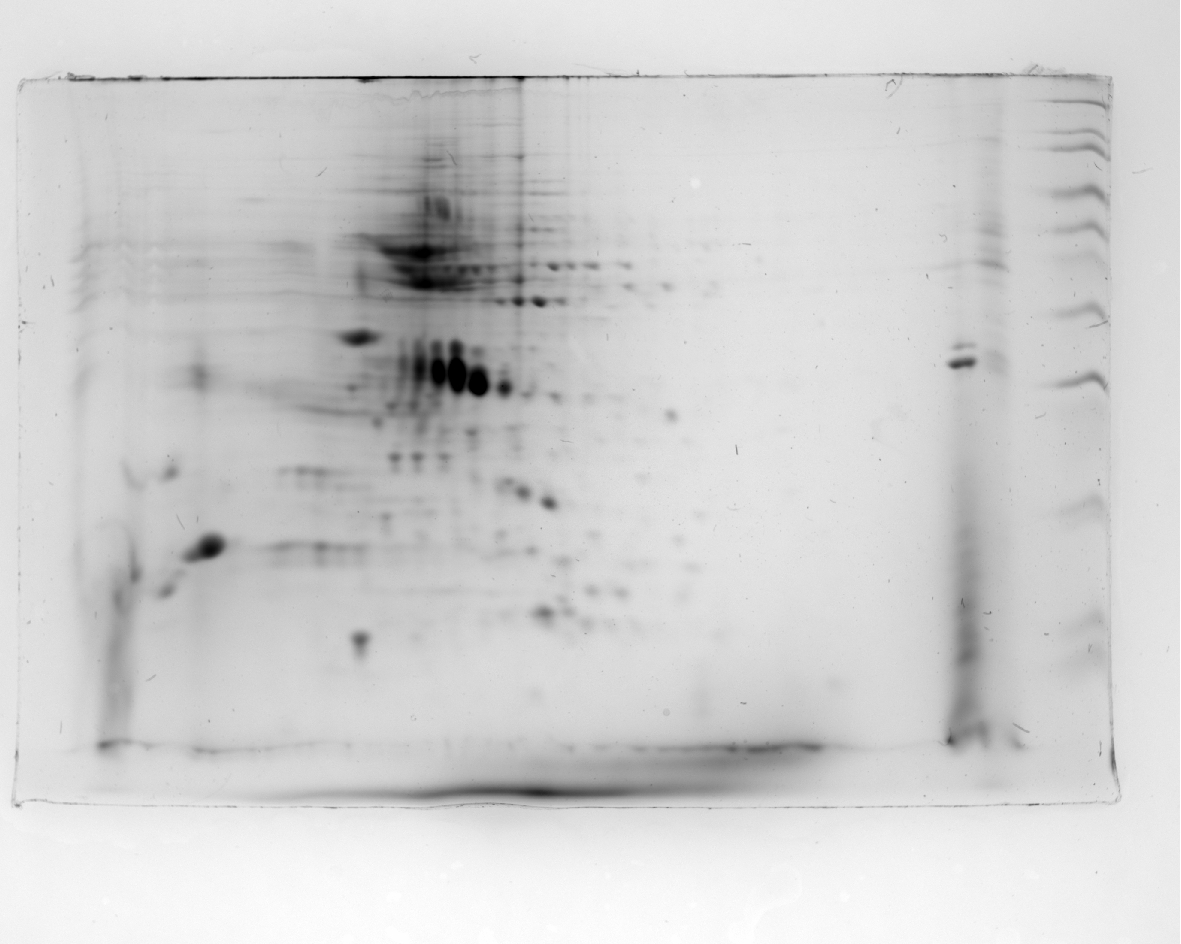


c)


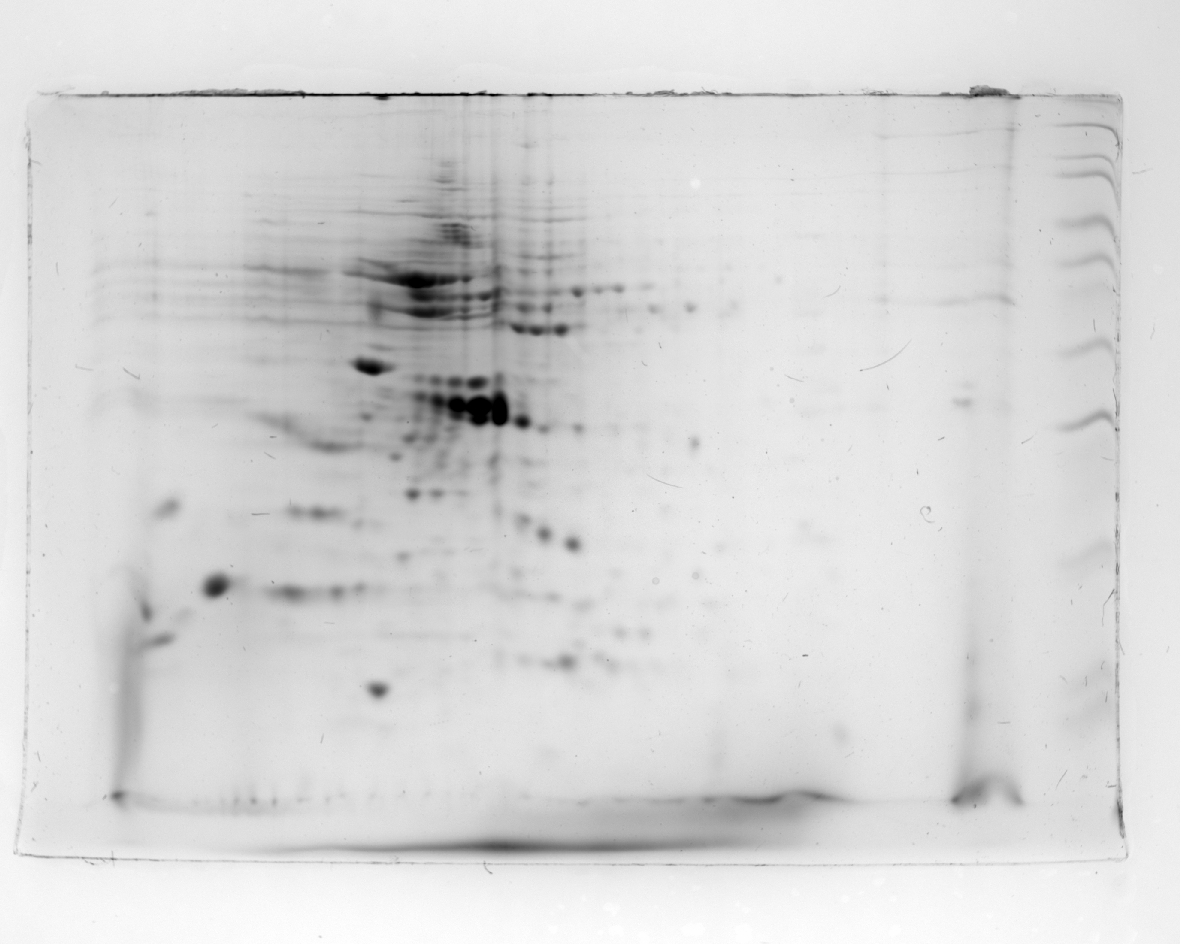


d)


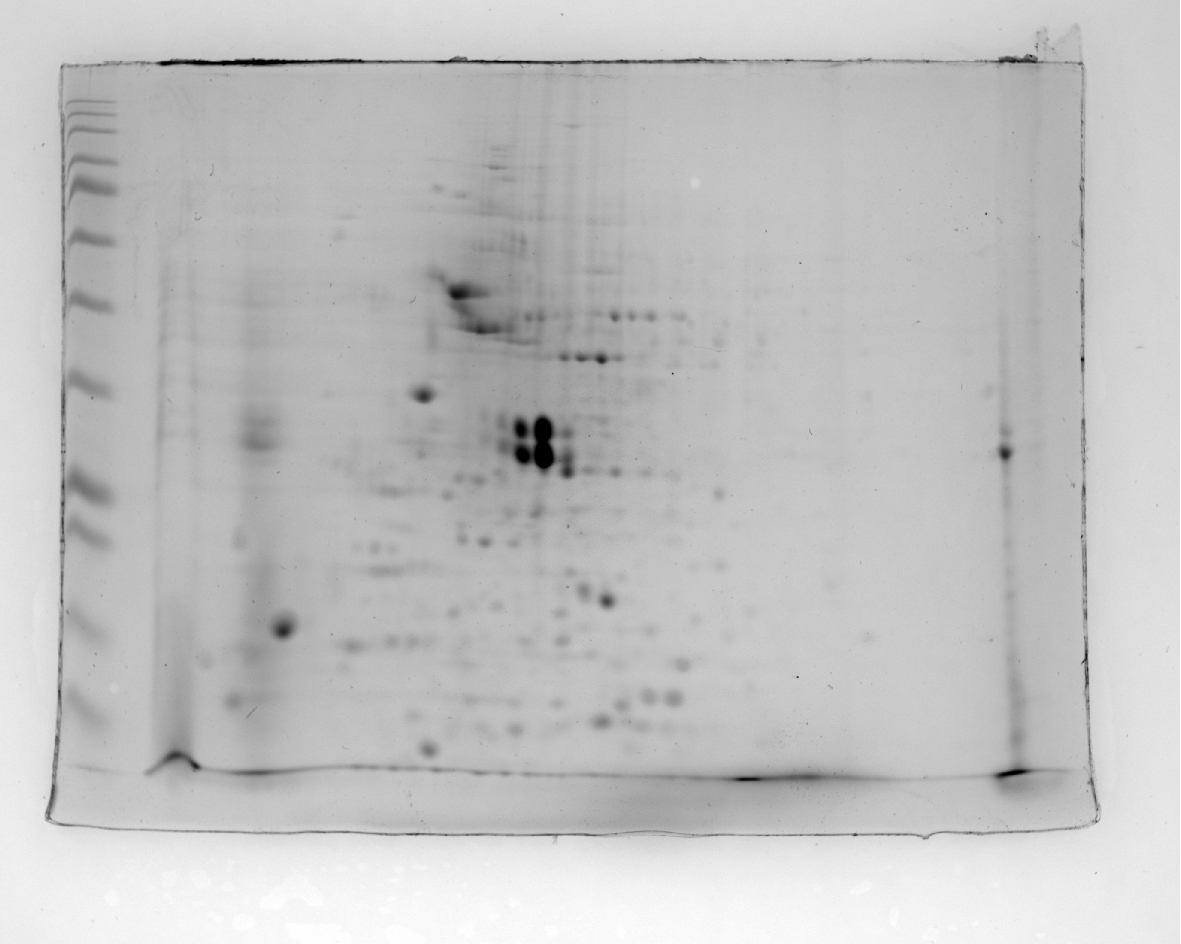


e)


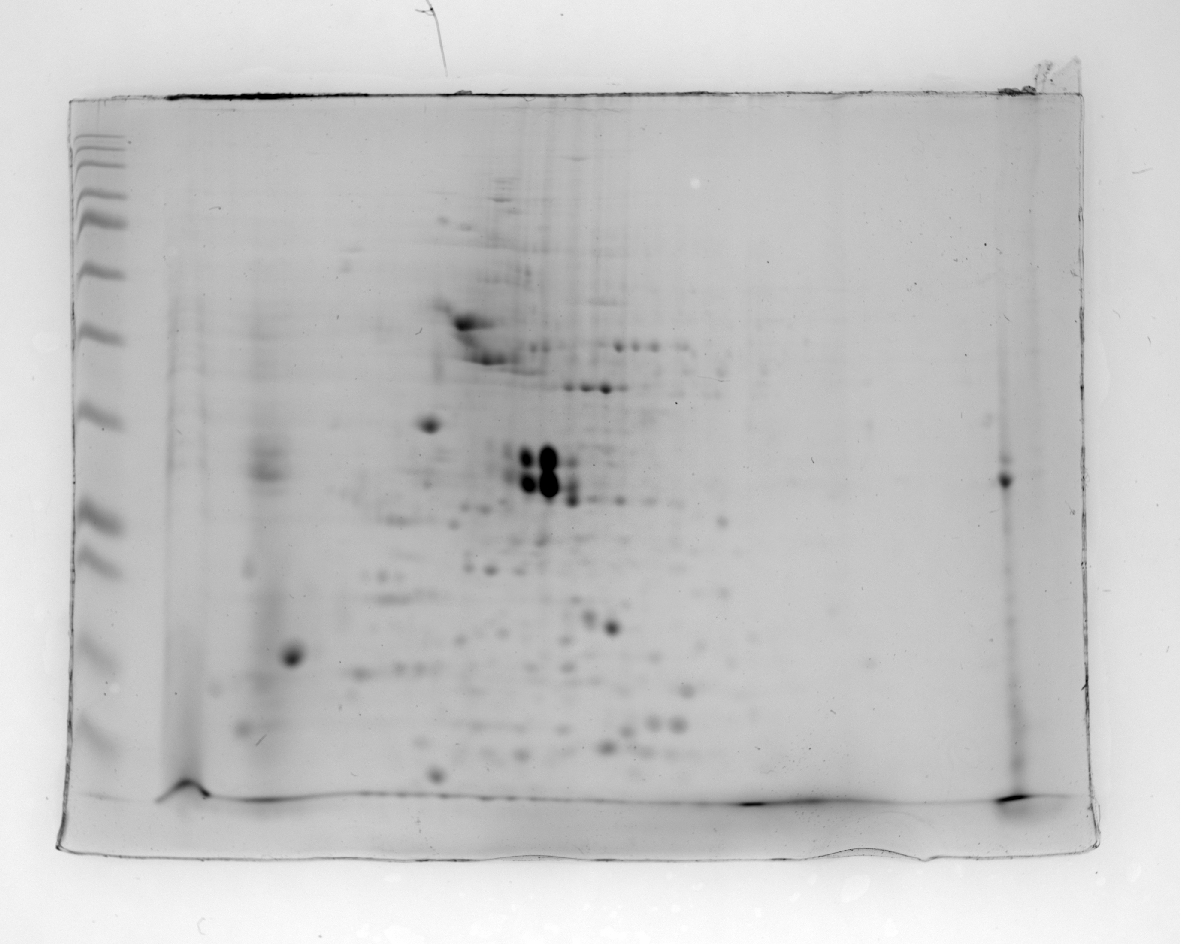


f)


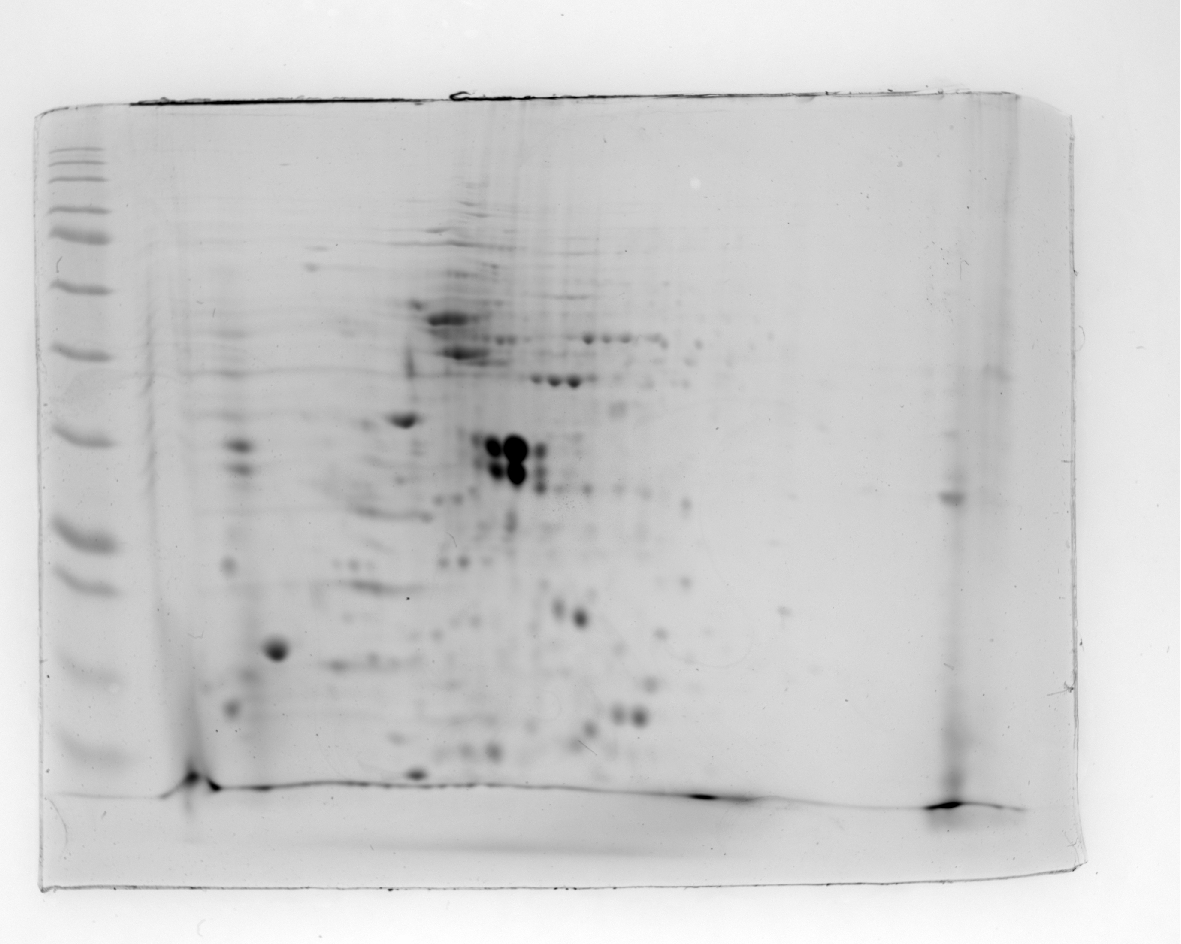


g)


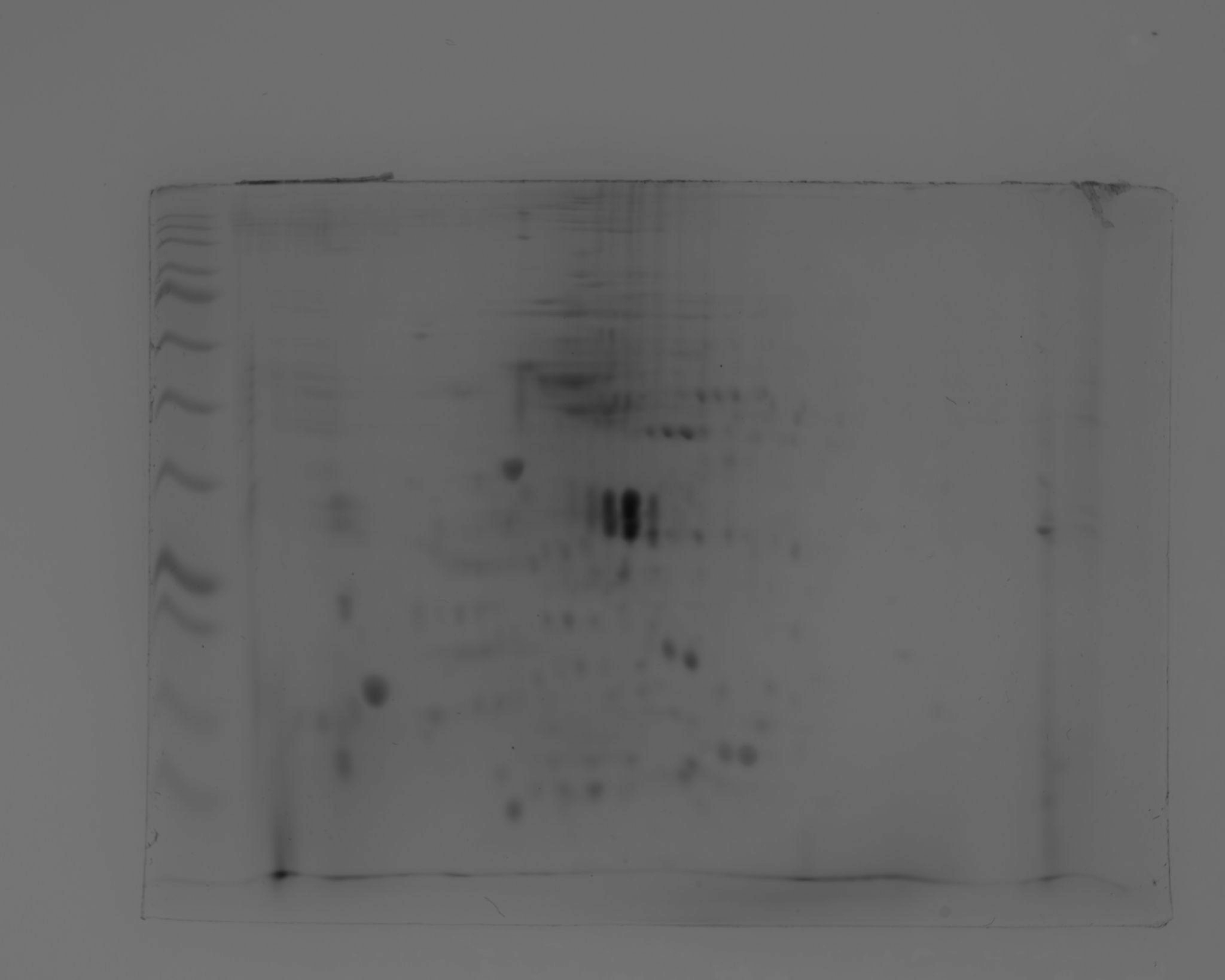


h)


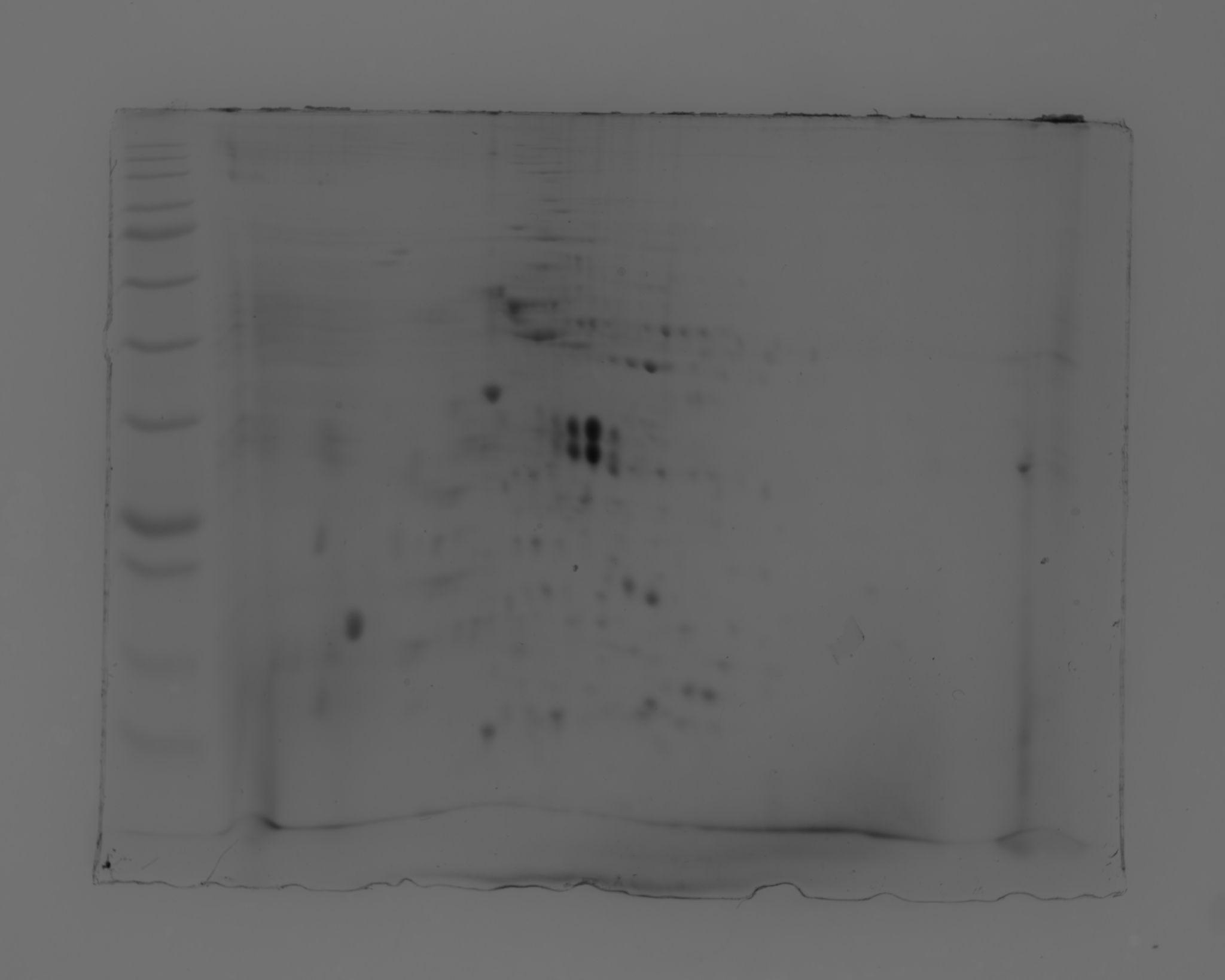


i)


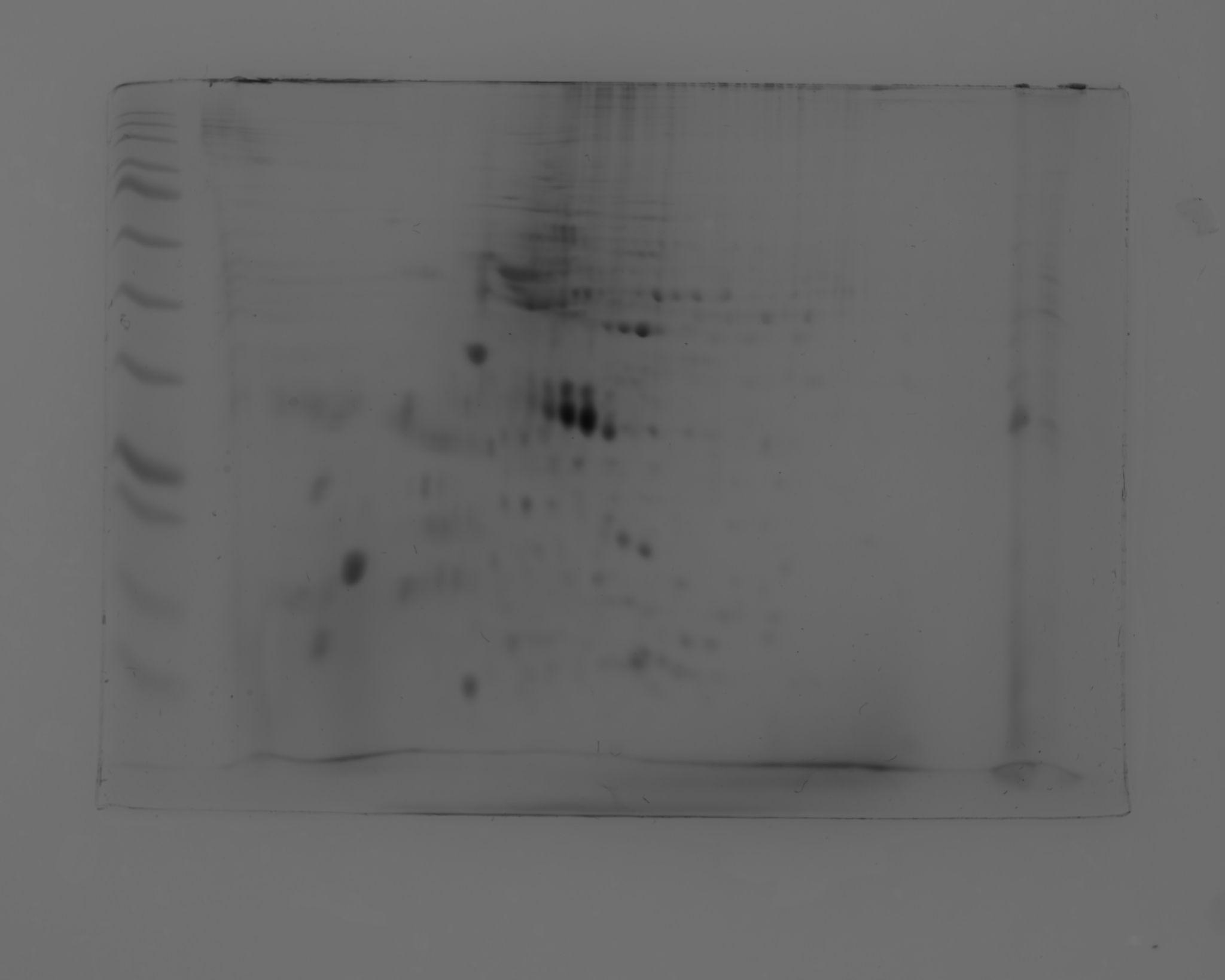


**Supplementary Figure 8**

PDQuest differential proteins analysis. In this figure master gels are shown, this indicates the union of the triplicate of each treatment (mixture 1 or DEET) with the control (non-treated mosquitoes) to identify the differential spots (shown as green crosses). a) Mixture 1 (geranyl acetate, α-bisabolol, and nerolidol) each molecule at 100 mg/mL, b) DEET treatment at 100 mg/mL.




**Supplementary Figure 9.**

Differential proteins Venn diagram related to mixture 1 (geranyl acetate, α-bisabolol, and nerolidol) and DEET treatments at 100 mg/mL. a) A total number of differential protein spots obtained. b) a Total number of identified proteins through MALDI-TOF MS. The proteins listed can be searched in the Supplementary Table 1.



**Supplementary Table 2.**

Differential proteins were identified through MALDI TOF MS regarding the mixture 1 (geranyl acetate, α-bisabolol, and nerolidol) treatment. For each protein, the score, expect, % sequence coverage, mass matched and the foldchange values are shown ($\geq$ 2.0 if the protein showed overexpression in comparison with the control after exposing the mosquito to the mixture 1 treatment; $\leq$ 0.5 if the protein showed downregulated expression under the same experimental conditions). Identification software MASCOT Science, Swissprot database.

| **No.** | **Protein** | **Score** | **Expect** | **% Sequence coverage** | **Mass value matched** | **Foldchange** |
| --- | --- | --- | --- | --- | --- | --- |
| **1** | Ribitol-5-phosphate cytidylyltransferase | 51 | 4,7 | 66 | 13/64 | 2.0 |
| **2** | LIM domain-binding protein 2 | 48 | 9.6 | 38 | 14/64 | 0.5 |
| **3** | Aldo-keto reductase | 55 | 1,9 | 24 | 8/23 | 2.0 |
| **4** | Apolipoprotein A-II | 56 | 1,4 | 72 | 12/63 | 2.0 |
| **5** | Putative pterin-4-alpha-carbinolamine dehydratase | 48 | 8.9 | 81 | 9/98 | 2.0 |
| **6** | Dihydroxyacetone phosphate acyltransferase | 56 | 1.5 | 50 | 17/37 | 2.0 |
| **7** | 6-phospho-5-dehydro-2-deoxy-D-gluconate aldolase | 45 | 19 | 20 | 6/26 | 0.5 |
| **8** | Ribonuclease HII | 50 | 6.3 | 53 | 17/65 | 2.0 |
| **9** | Flavin prenyl transferase | 46 | 14 | 37 | 11/86 | 2.0 |
| **10** | Guanylate kinase | 50 | 6.3 | 42 | 12/50 | 0.5 |
| **11** | Bifunctional protein FolD | 51 | 4.9 | 52 | 20/98 | 2.0 |
| **12** | Nucleoporin GLE1 | 52 | 3.5 | 18 | 15/30 | 0.5 |
| **13** | 60 kDa chaperonin | 64 | 0.22 | 43 | 17/50 | 0.5 |
| **14** | Acetyl glutamate kinase | 48 | 8.5 | 35 | 7/36 | 2.0 |
| **15** | No identified | - |  |  |  | 2.0 |
| **16** | Exocyst complex component EXO84 | 53 | 3.1 | 32 | 20/31 | 0.5 |
| **17** | Guanine nucleotide-binding protein alpha-3 subunit | 66 | 0.13 | 52 | 19/58 | 0.5 |
| **18** | Malate dehydrogenase | 40 | 53 | 44 | 7/38 | 0.5 |
| **19** | Ribosome maturation factor RimP | 49 | 7.8 | 74 | 11/98 | 0.5 |
| **20** | Ubiquinone biosynthesis protein COQ4 homolog | 44 | 20 | 45 | 18/93 | 2.0 |
| **21** | Protein translocase subunit | 55 | 1.7 | 19 | 15/51 | 0.5 |
| **22** | 3-oxoacyl-[acyl-carrier-protein] synthase 3 | 39 | 73 | 19 | 12/94 | 0.5 |
| **23** | Histidinol-phosphate aminotransferase | 50 | 6 | 34 | 13/57 | 0.5 |
| **24** | Squalene monooxygenase | 63 | 0.28 | 24 | 13/25 | 0.5 |
| **25** | Glyceraldehyde-3-phosphate dehydrogenase | 51 | 4.6 | 42 | 14/45 | 2.0 |
| **26** | GMP reductase | 66 | 0.14 | 54 | 24/98 | 2.0 |
| **27** | Sclerostin domain-containing protein 1 | 40 | 63 | 34 | 11/99 | 0.5 |
| **28** | NAD kinase | 44 | 22 | 33 | 8/48 | 0.5 |
| **29** | Polyketide synthase 2 | 46 | 15 | 25 | 35/62 | 0.5 |
| **30** | Calmodulin-regulated spectrin-associated protein 1-B | 54 | 2.2 | 14 | 18/30 | 0.5 |
| **31** | GTP cyclohydrolase-2 | 54 | 2.4 | 65 | 18/98 | 2.0 |
| **32** | Superoxide dismutase [Fe] | 46 | 14 | 40 | 7/37 | 0.5 |
| **33** | 4-diphosphocytidyl-2-C-methyl-D-erythritol kinase | 66 | 0.13 | 44 | 16/30 | 0.5 |
| **34** | High-affinity nitrate transporter 3.1 | 52 | 3.6 | 46 | 10/52 | 0.5 |
| **35** | Ribosomal RNA large subunit methyltransferase | 52 | 3.6 | 30 | 7/30 | 0.5 |
| **36** | ATP synthase subunit beta | 60 | 0.55 | 40 | 13/56 | 0.5 |
| **37** | Probable alpha-L-glutamate ligase | 48 | 10 | 27 | 8/23 | 0.5 |
| **38** | Protein lin-7 homolog B | 38 | 83 | 39 | 6/46 | 0.5 |
| **39** | Putative glutaredoxin-C14 | 46 | 14 | 90 | 10/98 | 2.0 |
| **40** | DNA ligase | 51 | 4.7 | 34 | 16/51 | 2.0 |
| **41** | Protein RecA | 51 | 4.4 | 20 | 8/12 | 0.5 |
| **42** | Protein vav | 52 | 3.2 | 18 | 17/22 | 0.5 |
| **43** | Regulator of rDNA transcription 14 | 50 | 5.8 | 41 | 9/41 | 0.5 |
| **44** | 50S ribosomal protein L16 | 50 | 5.9 | 53 | 6/60 | 0.5 |
| **45** | General odorant-binding protein 83a | 46 | 16 | 55 | 12/57 | 0.5 |
| **46** | Cyclic GMP-AMP synthase | 42 | 38 | 22 | 14/27 | 0.5 |
| **47** | UPF0365 protein BMD_4543 | 50 | 5.5 | N/A | N/A | 2.0 |
| **48** | Elongation factor Ts, mitochondrial | 56 | 1.6 | 53 | 20/98 | 2.0 |
| **49** | Chorismate synthase | 54 | 2.1 | 47 | 16/98 | 2.0 |
| **50** | 50S ribosomal protein L6 | 66 | 0.14 | 65 | 16/98 | 2.0 |
| **51** | Transcriptional repressor NrdR | 63 | 0.29 | 45 | 7/36 | 0.5 |
| **52** | No identified | - |  |  |  | 2.0 |
| **53** | 2-C-methyl-D-erythritol 2,4-cyclodiphosphate synthase | 50 | 5.4 | 55 | 7/31 | 0.5 |
| **54** | General odorant-binding protein 83a | 47 | 12 | 73 | 14/98 | 0.5 |
| **55** | 30S ribosomal protein S12 | 65 | 0.19 | 72 | 17/98 | 2.0 |
| **56** | No identified | - |  |  |  | 0.5 |
| **57** | Ephrin-B1 | 57 | 1.1 | 41 | 13/43 | 2.0 |
| **58** | Myosin-8 | 52 | 3.2 | 20 | 29/43 | 2.0 |
| **59** | Phospho-pantetheine adenylyl transferase | 50 | 5.1 | 83 | 15/98 | 2.0 |
| **60** | Protein lin-7 homolog B | 65 | 0.18 | 39 | 9/22 | 0.5 |
| **61** | Nicotinate phosphoribosyl transferase | 54 | 2.2 | 55 | 27/98 | 2.0 |
| **62** | 4-hydroxy-tetrahydrodipicolinate reductase | 51 | 4.7 | N/A | N/A | 2.0 |
| **63** | GTP pyrophosphokinase | 46 | 16 | 44 | 7/46 | 0.5 |
| **64** | Variable large protein 19 (Fragment) | 43 | 37 | 45 | 12/98 | 2.0 |
| **65** | Cyclic pyranopterin monophosphate synthase | 48 | 9.4 | 36 | 15/91 | 0.5 |
| **66** | No identified | - |  |  |  | 0.5 |
| **67** | Putative gustatory receptor 92a | 46 | 13 | 27 | 7/20 | 0.5 |
| **68** | General odorant-binding protein 83a | 46 | 16 | 55 | 12/57 | 0.5 |
| **69** | Glutathione S-transferase A | 58 | 0.8 | 45 | 9/34 | 2.0 |
| **70** | No identified | - |  |  |  | 0.5 |
| **71** | Nitric oxide synthase | 47 | 10 | 14 | 19/27 | 0.5 |
| **72** | N-acetylmuramic acid 6-phosphate etherase | 56 | 1.4 | 51 | 17/90 | 0.5 |
| **73** | Probable chemoreceptor glutamine de-amidase | 38 | 91 | 51 | 8/97 | 2.0 |
| **74** | Peroxiredoxin-2B | 53 | 2.8 | 63 | 15/98 | 2.0 |
| **75** | AIG2-like protein C | 50 | 5.9 | 46 | 10/98 | 2.0 |
| **76** | General odorant-binding protein 83a | 52 | 3.8 | 50 | 17/98 | 0.5 |
| **77** | Dynactin subunit 2 | 55 | 1.6 | 43 | 25/98 | 2.0 |
| **78** | Muscle LIM protein Mlp84B | 44 | 20 | 33 | 17/98 | 2.0 |
| **79** | Large-conductance mechanosensitive channel | 46 | 15 | 78 | 6/45 | 2.0 |
| **80** | No identified | - |  |  |  | 2.0 |
| **81** | No identified | - |  |  |  | 2.0 |
| **82** | No identified | - |  |  |  | 2.0 |
| **83** | Homologous-pairing protein 2 homolog | 50 | 6 | 63 | 26/98 | 2.0 |
| **84** | Probable GTP-binding protein EngB | 54 | 2 | 67 | 18/98 | 2.0 |
| **85** | Periplasmic nitrate reductase | 42 | 34 | 27 | 16/98 | 2.0 |
| **86** | Aspartate-tRNA ligase | 42 | 33 | N/A | N/A | 2.0 |
| **87** | Dihydroneopterin aldolase | 51 | 4.6 | 74 | 11/98 | 2.0 |
| **88** | Phosphoserine aminotransferase | 55 | 2 | 41 | 18/98 | 2.0 |
| **89** | 50S ribosomal protein L27 | 63 | 0.28 | 79 | 15/98 | 2.0 |
| **90** | No identified | - |  |  |  | 2.0 |
| **91** | Plastid 30S ribosomal protein S12 | 60 | 0.5 | 50 | 13/97 | 2.0 |
| **92** | Peroxisomal membrane protein | 53 | 2.6 | 75 | 19/98 | 2.0 |
| **93** | No identified | - |  |  |  | 2.0 |
| **94** | No identified | - |  |  |  | 2.0 |
| **95** | No identified | - |  |  |  | 2.0 |
| **96** | No identified | - |  |  |  | 2.0 |
| **97** | No identified | - |  |  |  | 2.0 |
| **98** | No identified | - |  |  |  | 2.0 |
| **99** | No identified | - |  |  |  | 2.0 |
| **100** | No identified | - |  |  |  | 2.0 |
| **101** | No identified | - |  |  |  | 2.0 |
| **102** | No identified | - |  |  |  | 2.0 |
| **103** | No identified | - |  |  |  | 2.0 |
| **104** | No identified | - |  |  |  | 2.0 |
| **105** | No identified | - |  |  |  | 2.0 |
| **106** | No identified | - |  |  |  | 2.0 |

**Supplementary Table 3.**

Differential proteins identified through MALDI-TOF MS regarding the DEET treatment. For each protein, the score, expect, % sequence coverage, mass matched and the foldchange values are shown (2.0 if the protein showed overexpression in comparison with the control after exposing the mosquito to the DEET treatment; 0.5 if the protein showed downregulated expression under the same experimental conditions). Besides, the animal species of the identified protein is displayed in the table. Identification software MASCOT Science, Swissprot database.

| **No.** | **Protein** | **Score** | **Expect** | **% Sequence coverage** | **Mass value matched** | **Foldchange** |
| --- | --- | --- | --- | --- | --- | --- |
| **1** | Acetyl-coenzyme A synthetase OS | 50 | 5.1 | 28 | 24/48 | 0.5 |
| **2** | DNA-directed RNA polymerase subunit beta | 48 | 8.3 | 23 | 25/56 | 0.5 |
| **3** | SUN domain-containing protein 2 | 62 | 0.37 | 47 | 15/43 | 0.5 |
| **4** | Glyceraldehyde-3-phosphate dehydrogenase | 51 | 4.6 | 42 | 14/45 | 0.5 |
| **5** | Bifunctional glucose-6-phosphate 1-dehydrogenase/6-phosphogluconolactonase | 50 | 5.9 | 16 | 15/25 | 0.5 |
| **6** | No identified | - |  |  |  | 0.5 |
| **7** | Leucine-rich repeat-containing protein 9 | 53 | 3.1 | 18 | 22/25 | 0.5 |
| **8** | Protein lin-7 homolog B | 38 | 83 | 39 | 6/46 | 0.5 |
| **9** | Ribosome maturation factor RimP | 48 | 8.7 | 82 | 10/43 | 0.5 |
| **10** | 3-oxoacyl-[acyl-carrier-protein] synthase 3 | 39 | 73 | 19 | 12/94 | 0.5 |
| **11** | Dihydroxy acetone phosphate acyltransferase | 48 | N/A | N/A | N/A | 0.5 |
| **12** | Inositol-1,4,5-trisphosphate 3-kinase activity | 50 | N/A | N/A | N/A | 0.5 |
| **13** | Cyclic GMP-AMP synthase | 43 | 29 | 31 | 15/30 | 0.5 |
| **14** | Squalene monooxygenase SE2 | 63 | 0.28 | 24 | 13/25 | 0.5 |
| **15** | Sclerostin domain-containing protein 1 | 40 | 63 | 34 | 11/99 | 0.5 |
| **16** | 16 kDa calcium-binding protein | 64 | 0.21 | 48 | 9/21 | 0.5 |
| **17** | Aspartate-tRNA (Asp/Asn) ligase | 58 | 0.91 | 33 | 14/31 | 0.5 |
| **18** | Frequenin-1 | 42 | 39 | 38 | 8/29 | 0.5 |
| **19** | 50S ribosomal protein L10 | 53 | 2.6 | 65 | 10/45 | 0.5 |
| **20** | Ubiquinone biosynthesis protein COQ4 homolog, mitochondrial | 50 | 20 | 45 | 18/93 | 0.5 |
| **21** | LIM domain-binding protein 2 | 48 | 9.6 | 38 | 14/64 | 0.5 |
| **22** | 4-diphosphocytidyl-2-C-methyl-D-erythritol kinase | 66 | 0.13 | 44 | 16/30 | 0.5 |
| **23** | Polyamine amino propyl transferase | 60 | 0.62 | N/A | N/A | 0.5 |
| **24** | 4-hydroxy-tetrahydrodipicolinate reductase | 51 | 4.7 | N/A | N/A | 0.5 |
| **25** | Histone H2A | 46 | 16 | 41 | 8/28 | 0.5 |
| **26** | Nucleoporin GLE1 | 52 | 3.5 | 18 | 15/30 | 0.5 |
| **27** | SEC12-like protein 2 | 45 | 17 | 18 | 8/22 | 0.5 |
| **28** | ATP synthase subunit beta | 60 | 0.55 | 40 | 13/56 | 0.5 |
| **29** | 50S ribosomal protein L13e | 53 | 2.8 | 78 | 9/45 | ≤ 0.5 |
| **30** | MYG1 exonuclease | 45 | 16 | 26 | 8/22 | 0.5 |
| **31** | Transcriptional repressor NrdR | 63 | 0.29 | 45 | 7/36 | 0.5 |
| **32** | GTP cyclohydrolase-2 | 54 | 2.4 | 65 | 18/98 | 0.5 |
| **33** | Putative gustatory receptor 92a | 46 | 13 | 27 | 7/20 | 0.5 |
| **34** | Nucleoporin GLE1 | 49 | 7.6 | 19 | 16/30 | 0.5 |
| **35** | Protein vav | 52 | 3.2 | 18 | 17/22 | 0.5 |
| **36** | ATP synthase subunit alpha | 50 | 6.2 | 18 | 11/21 | 0.5 |
| **37** | NAD kinase | 44 | 22 | 33 | 8/48 | 0.5 |
| **38** | Ribonuclease 3 | 51 | 4.4 | 40 | 8/23 | 0.5 |
| **39** | Protein lin-7 homolog B | 65 | 0.18 | 39 | 9/22 | 0.5 |
| **40** | Ubiquinone biosynthesis protein COQ4 homolog, mitochondrial | 44 | 20 | 45 | 18/93 | 0.5 |
| **41** | Glutathione S-transferase A | 58 | 0.8 | 45 | 9/34 | 0.5 |
| **42** | Variable large protein 19 (Fragment) | 43 | 31 | 56 | 16/97 | 0.5 |
| **43** | Probable transcription factor KAN4 | 55 | 1.7 | 21 | 7/20 | 2.0 |
| **44** | Cytochrome c | 54 | 2 | 50 | 6/37 | 2.0 |
| **45** | Polyamine amino propyl transferase | 60 | 0.62 | N/A | N/A | 2.0 |
| **46** | Threonine--tRNA ligase OS | 48 | 8.7 | 20 | 12/25 | 2.0 |
| **47** | Protein translocase subunit SecA | 52 | 3.8 | 31 | 24/34 | 2.0 |
| **48** | Lysozyme C | 46 | 5.8 | 56 | 11/43 | 2.0 |
| **49** | No identified | - |  |  |  | 2.0 |
| **50** | No identified | - |  |  |  | 2.0 |
| **51** | No identified | - |  |  |  | 2.0 |
| **52** | Protein LST8 homolog | 41 | 42 | 24 | 9/33 | 2.0 |
| **53** | 50S ribosomal protein L5 | 45 | 20 | 41 | 8/27 | 2.0 |
| **54** | Ribosome-recycling factor | 47 | 11 | 51 | 13/28 | 2.0 |
| **55** | tRNA modification GTPase MnmE | 46 | 13 | 24 | 10/15 | 2.0 |
| **56** | Elongation factor Ts | 50 | 5.8 | 49 | 13/24 | 2.0 |
| **57** | Histone-lysine N-methyltransferase | 51 | 4.2 | 20 | 16/32 | 2.0 |
| **58** | Arginine biosynthesis bifunctional protein | 48 | 9.6 | 29 | 14/23 | 2.0 |
| **59** | Calmodulin-regulated spectrin-associated protein 2 | 47 | 12 | 17 | 28/41 | 2.0 |
| **60** | Phosphatidylserine decarboxylase | 44 | 23 | 24 | 7/34 | 2.0 |
| **61** | CST complex subunit STN1 | 48 | 8.7 | 22 | 10/26 | 2.0 |
| **62** | Proline--tRNA ligase | 47 | 11 | 22 | 14/31 | 2.0 |
| **63** | Phenylalanine--tRNA ligase alpha subunit | 46 | 16 | 28 | 10/30 | 2.0 |
| **64** | Nuclear hormone receptor family member nhr-61 | 52 | 3.2 | 31 | 11/26 | 2.0 |
| **65** | 50S ribosomal protein L19 | 51 | 4.9 | 46 | 7/25 | 2.0 |
| **66** | GTPase Der | 47 | 11 | 22 | 13/23 | 2.0 |
| **67** | Nitric oxide synthase | 47 | 12 | 13 | 19/25 | 2.0 |
| **68** | UDP-3-O-acyl-N-acetylglucosamine deacetylase | 49 | 11 | 40 | 12/27 | 2.0 |
| **69** | Fructose-1,6-bisphosphatase class 1 | 51 | 4.5 | 28 | 10/25 | 2.0 |
| **70** | ATP-dependent lipid A-core flippase | 55 | 1.7 | 20 | 11/39 | 2.0 |
| **71** | Histone H1E | 58 | 1 | 46 | 10/30 | 2.0 |
| **72** | No identified | - |  |  |  | 2.0 |
| **73** | No identified | - |  |  |  | 2.0 |
| **74** | Arginine biosynthesis bifunctional protein | 46 | 16 | 28 | 15/23 | 2.0 |
| **75** | Golgi apparatus membrane protein | 41 | 48 | 16 | 6/35 | 2.0 |
| **76** | ADP/ATP translocase 2 | 53 | 2.6 | 33 | 15/28 | 2.0 |

**Supplementary Table 4.**

Identified protein’s function related to the mixture 1 (geranyl acetate, α-bisabolol, nerolidol) at 100 mg/mL. The protein function was searched in the Uniprot database https://www.uniprot.org/.

| **No.** | **Protein** | **Function** |
| --- | --- | --- |
| **1** | Ribitol-5-phosphate cytidylyltransferase | Transferase. Involved in bacterial cell wall synthesis. |
| **2** | LIM domain-binding protein 2 | Implicated in the nervous system and somatic cells development. This protein is regulated by kinases. |
| **3** | Aldo-keto reductase | Detoxification protein. This protein works with p450 and glutathione-S-transferase in other processes. |
| **4** | Apolipoprotein A-II | This protein interacts with lipids to stabilize cell structures. Related to signaling processes in which hormones, neurotransmitters, or secondary messengers are involved. |
| **5** | Putative pterin-4-alpha-carbinolamine dehydratase | Belongs to proteins that synthesize dihydrobiopterin and tetrahydrobiopterin cofactors. These cofactors are related to serotonin, melatonin, dopamine, norepinephrine neurotransmitter synthesis. |
| **6** | Dihydroxyacetone phosphate acyltransferase | Changes a phosphate group into an acetyl phosphate using acetyl-CoA. |
| **7** | 6-phospho-5-dehydro-2-deoxy-D-gluconate aldolase | Involved in acetyl-CoA biosynthesis from polyalcohol molecules in plans. It might be involved in the glycolysis process as other reaction products are 3-oxopropanoate and dihydro-acetone phosphate. |
| **8** | Ribonuclease HII | This protein helps RNA and DNA-RNA degradation. |
| **9** | Flavin prenyl-transferase | One of the proteins that belong to the ubiquinone biosynthesis (electron transport chain). |
| **10** | Guanylate kinase | Transferase and kinase, phosphorylate a guanosine monophosphate molecule (GMP) into guanosine diphosphate (GPD). This molecule works as a secondary messenger. |
| **11** | Bifunctional protein FolD | NADP-specific binding protein.  This protein belongs to tetrahydrofolate synthesis, a cofactor important for amino acid synthesis or methyl-transfer enzyme reactions. |
| **12** | Nucleoporin GLE1 | This protein helps in the mRNA transportation from the cell nucleus to the cytoplasm through nuclear pores. |
| **13** | 60 kDa chaperonin | This protein prevents improper protein unfolding/folding in the cytoplasm. |
| **14** | Acetyl glutamate kinase | Phosphotransferase. It belongs to urea cycle proteins and helps to synthesize some amino acids. |
| **16** | Exocyst complex component EXO84 | Involved in exocytosis processes, and endosome formation. |
| **17** | Guanine nucleotide-binding protein alpha-3 subunit | G protein. Involved in signaling processes. Alpha subunit: transform GTP into GDP. Besides, it is related to ionic channels regulated by K^+^ ions. |
| **18** | Malate dehydrogenase | Krebs cycle protein. This protein Belongs to the electron chain transport in the mitochondria to produce energy in the cell. |
| **19** | Ribosome maturation factor RimP | Important protein for 30S ribosome unit maturation. |
| **20** | Ubiquinone biosynthesis protein COQ4 homolog | This protein is implicated in the ubiquinone biosynthesis needed for electron chain transport. |
| **21** | Protein translocase subunit | Involved in the protein exportation through cell membranes. |
| **22** | 3-oxoacyl-[acyl-carrier-protein] synthase 3 | Lipid biosynthesis protein. |
| **23** | Histidine-ol-phosphate aminotransferase | This protein is implicated in histidine and other amino acids biosynthesis. |
| **24** | Squalene monooxygenase | Catalyze the stereospecific oxidation of squalene to epoxy-squalene. This step is the limiting step in steroid synthesis. |
| **25** | Glyceraldehyde-3-phosphate dehydrogenase | Glycolysis enzyme. Catalyze the glyceraldehyde 3-phosphate into glycerol 3-phosphate. |
| **26** | GMP reductase | Catalyze the irreversible reduction from GMP (guanosine monophosphate) into IMP (inosine monophosphate), an important reaction to maintain the adenine-guanine intracellular equilibrium. |
| **27** | Sclerostin domain-containing protein 1 | This protein is implicated in extracellular signaling processes, membrane receptors, homeostasis, cellular growth, apoptosis. |
| **28** | NAD kinase | Important protein to maintain the NAD-NADP balance in the cell. |
| **29** | Polyketide synthase 2 | Transferase enzyme. This protein catalyzes the acyl groups needed to synthesize metabolites in plants. |
| **30** | Calmodulin-regulated spectrin-associated protein 1-B | Cytoskeletal protein, nervous system cells mainly. It binds to microtubules due to the interaction with calmodulin/Ca^2+^ ions. |
| **31** | GTP cyclohydrolase-2 | It is involved in the purine metabolism in bacteria and fungi mostly. |
| **32** | Superoxide dismutase [Fe] | Detoxification enzyme. It degrades superoxide anions produced by cellular stress. |
| **33** | 4-diphosphocytidyl-2-C-methyl-D-erythritol kinase | It plays a part in the isopentenyl diphosphate biosynthesis route, a molecule needed for terpene, terpenoid, and lipid synthesis. |
| **34** | High-affinity nitrate transporter 3.1 | Transmembrane protein that allows nitrate ions to transport through the cell membrane. |
| **35** | Ribosomal RNA large subunit methyltransferase | RNA-binding protein. Transfers methyl groups to cytosine. |
| **36** | ATP synthase subunit beta | This protein belongs to the electron chain transport to produce energy (ATP) in the mitochondria. |
| **37** | Probable alpha-L-glutamate ligase | This enzyme leads to glutamate amino acid binding (Glu-Glu). Involved in glutathione synthesis. |
| **38** | Protein lin-7 homolog B | It maintains the proper order and performance of ionic channels in polarized/polarizable membranes. It is involved in synaptic protein localization and positioning during vesicle transport. |
| **39** | Putative glutaredoxin-C14 | In the presence of NADPH, break the bond that binds two glutathione molecules (disulfide bond breaking). It is considered a detoxification protein. |
| **40** | DNA ligase | It forms covalent bonding between the 5’ and 3’ extremes in polynucleotide chains. |
| **41** | Protein RecA | It is an essential protein to repair DNA. It protects DNA fragments from nuclease action. |
| **42** | Protein vav | It belongs to a signaling protein family. Its function is related to G proteins, GTP coupling, and GDP releasing in signaling processes in the cell. |
| **43** | Regulator of rDNA transcription 14 | The function of this protein is partially unknown. It is thought that it might be involved in ribosome maturation and formation. |
| **44** | 50S ribosomal protein L16 | Essential for tridimensional ribosome structure. |
| **45** | General odorant-binding protein 83a | Odorant transport across the olfactory system for insects. |
| **46** | Cyclic GMP-AMP synthase | Catalyzes the reaction guanosine monophosphate GMP to cyclic adenosine monophosphate cAMP, secondary messengers for signaling processes. |
| **47** | UPF0365 protein BMD_4543 | An integral membrane component. |
| **48** | Elongation factor Ts, mitochondrial | This protein binds with a complex that leads to change GDP into GTP in the mitochondria. The main function is to transport tRNAs in the ribosomes during the elongation process. |
| **49** | Chorismate (chorismic acid) synthase | Catalyzes the reaction that produces chorismic acid, an important molecule involved in phenylalanine, tyrosine, and tryptophan amino acid synthesis. Mainly important in bacteria but present in eukaryotic cells. |
| **50** | 50S ribosomal protein L6 | A structural component in ribosomes. |
| **51** | Transcriptional repressor NrdR | Involved in ribonucleotide reductase regulation during protein transcription. |
| **53** | 2-C-methyl-D-erythritol 2,4-cyclodiphosphate synthase | Important protein that helps to synthesize isoprenoid compounds. |
| **55** | 30S ribosomal protein S12 | Ribosome structural protein. It maintains the integrity of the ribosome structure during transcription processes. |
| **57** | Ephrin-B1 | This transmembrane protein is important for protein-receptor binding (Eph tyrosine kinase receptors). This protein is involved in protein-protein interaction in the neurons during neuron cell development. |
| **58** | Myosin-8 | Microfilament binding protein. Mostly present in muscle cells. |
| **59** | Phospho-pantetheine adenylyl-transferase | One of the proteins involved in the acetyl-CoA synthesis in bacteria. |
| **61** | Nicotinate phosphoribosyl transferase | Catalyzes the first step for NAD synthesis from nicotinic acid. The NAD production avoids cellular stress generated by oxidant agents. |
| **62** | 4-hydroxy-tetrahydrodipicolinate reductase | A protein involved in the lysine biosynthesis. |
| **63** | GTP pyrophosphokinase | It helps to produce AMP from ATP and GTP. The AMP is a secondary messenger involved in signaling routes. |
| **64** | Variable large protein 19 (Fragment) | Extracellular protein that is expressed in response to immune system activation. |
| **65** | Cyclic pyranopterin monophosphate synthase | One of the proteins implicated in the molybdopterin biosynthesis, an important cofactor to some oxidases. |
| **67** | Putative gustatory receptor 92a | A gustatory receptor that is responsible for discerning attractiveness behavior in insects. It is expressed in the olfactory system. |
| **69** | Glutathione S-transferase A | Important detoxification protein. It catalyzes the reaction between a reduced glutathione molecule and a xenobiotic substrate. |
| **71** | Nitric oxide synthase | It is implicated in nitric oxide production (NO). This molecule can interact as a neurotransmitter that activates K^+^ channels, which leads to membrane hyperpolarization. It also stimulates kinase protein activation. |
| **72** | N-acetylmuramic acid 6-phosphate etherase | It belongs to the *N-*acetyl muramic degradation route. It also helps in the carbohydrates and amino acid metabolism in bacteria. |
| **73** | Probable Chemoreceptor glutamine deamidase | A Chemoreceptor important in the chemotaxis process in bacteria mainly. This process helps the cell perceive lower or greater concentrations of a certain molecule. |
| **74** | Peroxiredoxin-2B | Detoxification protein that helps to reduce hydrogen peroxide and alkyl-hydroperoxides during cellular stress. |
| **75** | AIG2-like protein C | This protein is implied in the oxo-proline synthesis important in homeostasis. It might produce the cytochrome c release leading to apoptosis. |
| **77** | Dynactin subunit 2 | It modulates the dynein binding with the microtubules and cellular organelles. It is thought to be important in brain growth and development in mammals. |
| **78** | Muscle LIM protein Mlp84B | Important for microtubule formation. It promotes the cytoskeletal organization during tissue growth and development. |
| **79** | Large-conductance mechanosensitive channel | Mechanosensitive ion channel. It takes part in osmotic pressure regulation inside the cell. |
| **83** | Homologous-pairing protein 2 homologs | This protein is important during meiosis cell division. It helps the chromosome coupling process during cellular division. |
| **84** | Probable GTP-binding protein EngB | GTP and GDP binding protein that allows proper signaling processes. |
| **85** | Periplasmic nitrate reductase | It transforms nitrate into nitrite in the cell. Besides, it is involved in nitrogen oxides synthesis important for amino acids or nucleotide synthesis. |
| **86** | Aspartate-tRNA ligase | Catalyzes the aspartate-tRNA binding. ATP is required for this reaction. |
| **87** | Dihydro-neopterin aldolase | It participates in tetrahydrofolate biosynthesis, B9 vitamin, and folic acid precursor. |
| **88** | Phosphoserine aminotransferase | Catalyzes glutamate synthesis. Moreover, it participates in serine amino acid synthesis. |
| **89** | 50S ribosomal protein L27 | A ribosome structural compound. |
| **91** | Plastid 30S ribosomal protein S12 | A structural compound important during the transcription process. It is located in either 30S or 50S ribosomal subunits. |
| **92** | Peroxisomal membrane protein | Required protein for peroxisome assembly. It can work as a receptor for protein importation into the peroxisome. |

**Supplementary Table 5.**

Identified protein’s function related to the DEET synthetic repellent at 100 mg/mL. The protein function was searched in the Uniprot database https://www.uniprot.org/.

| **No.** | **Protein** | **Function** |
| --- | --- | --- |
| **1** | Acetyl-coenzyme A synthetase | Catalyzes the acetate and CoA reaction into acetyl-CoA, an important molecule for energy production during the citric acid cycle. |
| **2** | DNA-directed RNA polymerase subunit beta | It catalyzes the RNA fragments polymerization. |
| **3** | SUN domain-containing protein 2 | Cytoskeletal protein complex involved in the interaction and connection between the nucleus and cytoskeletal. |
| **4** | Glyceraldehyde-3-phosphate dehydrogenase | Glycolysis enzyme. Catalyzes the reaction that turns glyceraldehyde-3-phosphate into glycerol-3-phosphate. |
| **5** | Bifunctional glucose-6-phosphate 1-dehydrogenase/6-phosphogluconolactone | This protein catalyzes the first two steps in the pentose-phosphates oxidative route. It contributes to the reduction power during lipids and nucleotides synthesis. |
| **7** | Leucine-rich repeat-containing protein 9 | A Protein-rich in leucine amino acid. This protein is implicated in the adhesion process of other proteins during synapsis. It might be involved in vesicle formation during synapsis. |
| **8** | Protein lin-7 homolog B | It maintains the proper order and performance of ionic channels in polarized/polarizable membranes. It is involved in synaptic protein localization and positioning during vesicle transport. |
| **9** | Ribosome maturation factor RimP | An important protein for 30S ribosome unit maturation. |
| **10** | 3-oxoacyl-[acyl-carrier-protein] synthase 3 | Lipid biosynthesis protein. |
| **11** | Dihydroxyacetone phosphate acyltransferase | Changes a phosphate group into an acetyl phosphate using acetyl-CoA. |
| **12** | Inositol-1,4,5-trisphosphate 3-kinase activity | This protein regulates signaling processes related to Ca^2+^ ions. It is related to G proteins that trigger calcium ion release to the cytoplasm. This response leads to neurotransmitter releasing and transcriptional regulation. |
| **13** | Cyclic GMP-AMP synthase | Catalyzes the reaction guanosine monophosphate GMP to cyclic adenosine monophosphate cAMP, secondary messengers for signaling processes. |
| **14** | Squalene monooxygenase SE2 | Catalyze the stereospecific oxidation of squalene to epoxy-squalene. This step is the limiting step in steroid synthesis. |
| **15** | Sclerostin domain-containing protein 1 | This protein is implicated in extracellular signaling processes, membrane receptors, homeostasis, cellular growth, apoptosis. |
| **16** | 16 kDa calcium-binding protein | Low molecular weight protein that binds to calcium ions Ca^2+^. |
| **17** | Aspartate-tRNA (Asp/Asn) ligase | It binds the aspartate amino acid to its corresponding tRNA. |
| **18** | Frequenin-1 | This protein cooperates during the signaling transmission in processes like neuronal synapses. It depends on calcium ions for proper functioning. |
| **19** | 50S ribosomal protein L10 | Ribosome structural protein. Interacts with GTP-binding proteins. |
| **21** | LIM domain-binding protein 2 | Implicate in the nervous system and somatic cells development. This protein is regulated by kinases. |
| **22** | 4-diphosphocytidyl-2-C-methyl-D-erythritol kinase | It plays a part in the isopentenyl diphosphate biosynthesis route, a molecule needed for terpene, terpenoid, and lipid synthesis. |
| **23** | Polyamine aminopropyl transferase | It is involved in polyamine synthesis and polymerization, in bacteria mostly. In other cells, it helps to produce spermidine production, a molecule that maintains membrane potential, intracellular pH, and nitric oxide (NO) production. |
| **24** | 4-hydroxy-tetrahydrodipicolinate reductase | A protein involved in the lysine biosynthesis. |
| **25** | Histone H2A | Histone protein that is involved in the chromatin formation in eukaryotic cells. |
| **26** | Nucleoporin GLE1 | This protein helps in the mRNA transportation from the cell nucleus to the cytoplasm through nuclear pores. |
| **27** | SEC12-like protein 2 | A required protein for vesicle formation and development in the endoplasmic reticulum. |
| **28** | ATP synthase subunit beta | This protein belongs to the electron chain transport to produce energy (ATP) in the mitochondria. |
| **29** | 50S ribosomal protein L13e | Essential for tridimensional ribosome structure. |
| **30** | MYG1 exonuclease | It is involved in the RNA (3’-5’) *in situ* transcripts breaking in the nucleus or mitochondria. It is also important in the transcription process and DNA repair. |
| **31** | Transcriptional repressor NrdR | Related to the regulation of ribonucleotide reductases in the transcription processes. |
| **32** | GTP cyclohydrolase-2 | It is involved in the purine metabolism in bacteria and fungi mostly. |
| **33** | Putative gustatory receptor 92a | A gustatory receptor responsible for discerning attractiveness behavior in insects. It is expressed in the olfactory system. |
| **35** | Protein vav | Signaling protein. It functions as a G protein and helps to the GTP-binding and GDP releasing in signaling processes. |
| **36** | ATP synthase subunit alpha | It belongs to the electron chain transport that produces ATP (energy) from ADP. The alpha and beta subunits participate in the catalytic site involved in the movement of other ATP synthase subunits, producing ATP hydrolysis. |
| **37** | NAD kinase | Important protein to maintain the NAD-NADP balance in the cell. |
| **38** | Ribonuclease 3 | It helps RNA and RNA-DNA derivative degradation. |
| **40** | Ubiquinone biosynthesis protein COQ4 homolog, mitochondrial | This protein is implicated in the ubiquinone biosynthesis needed for electron chain transport. |
| **41** | Glutathione S-transferase A | Important detoxification protein. It catalyzes the reaction between a reduced glutathione molecule and a xenobiotic substrate. |
| **42** | Variable large protein 19 (Fragment) | Extracellular protein that is expressed in response to immune system activation. |
| **43** | Probable transcription factor KAN4 | A possible transcription factor that regulates tissue, skin, shell formation in an organism. |
| **44** | Cytochrome c | An electron transport protein. It is important during cellular stress exposure triggering the caspase activation leading to apoptosis. |
| **46** | Threonine-tRNA ligase | Catalyzes the reaction that binds threonine with its respective tRNA. |
| **47** | Protein translocase subunit SecA | A protein that is required for protein transport across the cell membrane. |
| **48** | Lysozyme C | Protein that belongs to the innate immune system with antimicrobe enzymatic activity. It hydrolyzes the *N-*acetyl muramic acid and *N-*acetyl glucosamine present in the bacteria cell wall. |
| **52** | Protein LST8 homolog | It is a TORC1/TORC2 complex subunit that regulates cell growth. Besides, it helps protein synthesis, nutrient degradation, and regulate growth factors. |
| **53** | 50S ribosomal protein L5 | Ribosome structural protein. It helps to bind smaller ribosome units in the ribosome complex. |
| **54** | Ribosome-recycling factor | In the mitochondria, it contributes to ribosome recycling after the transcription process. |
| **55** | tRNA modification GTPase MnmE | Induces tRNA modification with a carboxy amino methyl addition. Moreover, it helps to transform GTP into GDP transferring the carboxy aminomethyl group in the tRNAs. |
| **56** | Elongation factor Ts | It helps the elongation process during transcription. It catalyzes the GDP release by GTP breaking. It is needed for protein elongation and aminoacyl tRNA addition. |
| **57** | histone-lysine *N*-methyltransferase | This protein is involved in regulatory processes in the replication and transcription, mostly in plants. It allows signal modulation during cell development. |
| **58** | Arginine biosynthesis bifunctional protein | This protein catalyzes reactions involved in arginine biosynthesis. Acetyl glutamate synthesis from glutamate and acetyl-CoA. Ornithine synthesis from acetyl-ornithine and glutamate. |
| **59** | Calmodulin-regulated spectrin-associated protein 2 | Important for microtubule organization and movement. The protein located in the neurons works as a growth regulator for axon and dendrite. |
| **60** | Phosphatidylserine decarboxylase | It takes part in phospholipid metabolism. It helps to synthesize phosphatidylethanolamine from phosphatidylserine, molecules important in membrane structure. |
| **61** | CST complex subunit STN1 | One of the CST complex components. It is implicated in telomere maintenance during DNA replication. |
| **62** | Proline-tRNA ligase | Catalyzes the proline binding to its respective tRNA. During this process, an ATP molecule is consumed. |
| **63** | Phenylalanine-tRNA ligase alpha subunit | It catalyzes the phenylalanine binding to its respective tRNA. During this process, an ATP molecule is consumed. |
| **64** | Nuclear hormone receptor family member nhr-61 | Nuclear receptor, it is unknown which ligands can bind with the receptor. |
| **65** | 50S ribosomal protein L19 | A ribosomal structural protein important for ribosome assembly. |
| **66** | GTPase Der | GTPase activity. It is important for ribosome assembly. |
| **67** | Nitric oxide synthase | It is implicated in nitric oxide production (NO). This molecule can interact as a neurotransmitter that activates K^+^ channels, which leads to membrane hyperpolarization. It also stimulates kinase protein activation. |
| **68** | UDP-3-O-acyl-*N*-acetylglucosamine deacetylase | Catalyzes the UDP-acyl-N-acetyl glucosamine hydrolysis leading to UDP-myristoyl glucosamine, an important molecule for membrane lipid synthesis in bacteria. |
| **69** | Fructose-1,6-bisphosphatase class 1 | An important enzyme for gluconeogenesis regulation. It catalyzes the elimination of a phosphate group from fructose 1,6-bisphosphate forming fructose 6-phosphate. |
| **70** | ATP-dependent lipid A-core flippase | Implicated in the lipopolysaccharide biosynthesis. It is located in the cell membrane and helps to move lipids in between the cell membrane. |
| **71** | Histone H1E | It is involved in the DNA chromatin fibers formation. |
| **75** | Golgi apparatus membrane protein | A transmembrane protein that is involved in the protein and vesicle exportation from the Golgi apparatus. |
| **76** | ADP/ATP translocase 2 | A mitochondria membrane protein that catalyzes the ADP-ATP exchange. The ADP comes from the cytoplasm and the ATP from mitochondria. |

**Supplementary Table 6.**

Protein-protein interactions list related to the mixture 1 (geranyl acetate, α-bisabolol, and nerolidol) at concentration 100 mg/mL interactome. STRING Software for protein-protein interactions.

| **Cluster** | **Protein** | **Name in the interactome** |
| --- | --- | --- |
| Transport, vesicle formation and synapsis-related proteins | Exocyst complex component1 | Exocys1 |
|  | Exocyst complex component7 | Exocys7 |
|  | Exocyst complex component5 | Exocys5 |
|  | Exocyst complex component8 | Exocys8 |
|  | Exocyst complex component15 | Exocys15 |
|  | Dynactin subunit 2 | Dynactin2 |
|  | Dynactin subunit 5 | Dynactin5 |
|  | Calmodulin-regulated spectrin-associated protein 1-B | SpectrinCa |
|  | Probable GTP-binding protein | GTPbind |
|  | Protein Vav | Vav |
|  | Putative gustatory receptor 92a | GR92a |
|  | General odorant-binding protein 83a | OBP83a |
| Proteins necessary to ATP synthesis, detoxification proteins, and neurotransmitter synthesis | Guanylate kinase | Gkinase |
|  | Nitric oxide synthase | NOSynthase |
|  | Superoxide dismutase [Fe] | SOxiD |
|  | Malate dehydrogenase | MalateDH |
|  | ATP synthase subunit beta | ATPasaBeta |
|  | Dihydroxyacetone phosphate acyltransferase | DhapAcT |
|  | 60 kDa chaperonin | Chaperonin |
|  |  |  |
|  | Ubiquinone biosynthesis protein COQ4 homolog | UbiquinoneS |
|  | Glyceraldehyde-3-phosphate dehydrogenase | G3PDH |
|  | Phospho-pyruvate hydratase | Enolase |
| Nuclear proteins. Protein and amino acid synthesis. Detoxification proteins | Nucleoporin GLE1 | Nucleoporin |
|  | Nucleopore | Nucleopore |
|  | Nuclearpore88 | Nuclearpore88 |
|  | Muscle LIM protein Mlp84B | LIMprot |
|  | DNA Ligase III | DNALigase |
|  | Ribonuclease HII | RibonucleaseH |
|  | DNA Binding Protein | DNABinding |
|  | NAD Binding Protein | NADBinding |
|  | Glutamate Proline tRNA ligase | GluProtRNAL |
|  | Phosphoserine aminotransferase | PSerAmTrans |
|  | Aspartate-tRNA ligase | AsptRNALigase |
|  | Glutathione S-transferase A | GHSTransf |
|  | Nicotinate phosphoribosyl transferase | NicoPRTransf |
| Ribosomal proteins, signaling proteins and lipid synthesis | 3-oxoacyl-[acyl-carrier-protein] synthase 3 | 3oxoAcprot |
|  | NAD kinase | NADkinase |
|  | Phospho-pantetheine adenylyltransferase | PAdenylTransf |
|  | Ribosome maturation factor RimP | RiboAssembly |
|  | Ribosome Biogenesis factor | RiboBio |
|  | Probable Ribosome Biogenesis factor 24 | RiboBio24 |
|  | Ribosome production protein (Brix domain-containing protein 1 homolog) | RiboProduct |

**Supplementary Table 7.**

*Protein-protein interactions list related to the DEET synthetic repellent at concentration 100 mg/mL interactome. STRING Software for protein-protein interactions.*

| **Cluster** | **Protein** | **Name in the interactome** |
| --- | --- | --- |
| Signaling proteins and synapsis-related proteins | Frequenin-1 | Frequenin |
|  | Putative gustatory receptor 92a | GR92a |
|  | NAD kinase | NADkinase |
|  | Protein vav | Vav |
|  | Leucine-rich repeat-containing protein 9 | Leu-Gprot |
| Proteins necessary for ATP synthesis | ATP synthase subunit alpha | ATPasaAlfa |
|  | ATP synthase subunit beta | ATPasabeta |
|  | ATP synthase subunit b | ATPasaSubB |
|  | ATP synthase subunit f | ATPasaSubf |
|  | Protein Stunted | Stunted |
|  | Ubiquinone biosynthesis protein COQ4 | UbiquinoneS |
|  | Ubiquinone biosynthesis protein | UbiquinoneS2 |
|  | Cytochrome c | CytochromeC |
|  | ADP/ATP translocase 2 | ADP/ATP Carrier |
|  | Glyceraldehyde-3-phosphate dehydrogenase | G3PDH |
| Detoxification, nitric oxide synthesis | Nitric oxide synthase | NOSynthase |
|  | Phosphatidylserine decarboxylase | PSerineDC |
|  | Glutathione S-transferase A | GSHTr |
|  | Dihydroxyacetone phosphate acyltransferase | DhapAcT |
|  | Lysozyme C | Lysozyme |
| Gluconeogenesis proteins and lipid synthesis proteins | Fructose-1,6-bisphosphatase class 1 | Fruc1,6-BPase |
|  | Acetyl coenzyme A synthetase OS | AcCoASyn |
|  | 3-oxoacyl-[acyl-carrier-protein] synthase 3 | 3oxoAcprot |
| Signaling proteins, nuclear and ribosomal proteins. Protein synthesis processes | Inositol-1,4,5-trisphosphate 3-kinase activity | kinaseIPP_3_ |
|  | Rapamycin companion complex of Tor | Rapamycin |
|  | Nuclear pore complex 214 | Nuclear pore |
|  | Protein LST8 homolog | LST8 |
|  | Nucleoporin GLE1 | Nucleoporin |
|  | Histone H2A | His2A |
|  | Ribosome biogenesis regulatory protein | RiboBio |
|  | Ribosomal RNA processing 40 | Ribonuclease40 |
|  | Exoribonuclease Mtr3 | ERibonucleaseMtr3 |
|  | Exoribonuclease Rrp45 | ERibonuclease45 |
|  | Exoribonuclease Rrp4 | ERibonuclease4 |
|  | Ribonuclease 3 | ERibonuclease |
|  | Ribosome Assembly factor Mtr4 | Ribosome factor |
|  | Phenylalanine-tRNA ligase alpha subunit | Phe-tRNA ligase |
|  | Elongation factor Ts | Elongation fTS |
|  | Threonine-tRNA ligase | Thr-tRNA ligase |
|  | Proline-tRNA ligase | Pro-tRNA ligase |

**Supplementary Table 8.**

*Transcripts obtained of NCBI of some proteins with differential expression in Aedes aegypti according our discussion.*

| **Protein** | **ARN** | **Gen** | **XP** | **Link** |
| --- | --- | --- | --- | --- |
| Guanylate kinase | rna28548 | gene15883 | XP_001661287.1 | https://www.ncbi.nlm.nih.gov/gene/5574237 |
| Guanine nucleotide-binding protein | rna10321 | gene5744 | XP_001660884.1 | https://www.ncbi.nlm.nih.gov/gene/5573467 |
| Malate dehydrogenase | rna21070 | gene11710 | XP_001659012.2 | https://www.ncbi.nlm.nih.gov/gene/5570233 |
| Nitric oxide synthase | rna11255 | gene6263 | XP_001654595.2 | https://www.ncbi.nlm.nih.gov/gene/5573420 |
| General odorant-binding protein 83a | rna23722 | gene13308 | XP_001651429.2 | https://www.ncbi.nlm.nih.gov/gene/5567054 |

**Supplementary Figure 10.**

Promising detoxification processes implemented by the mosquito using the Aldo-keto reductase and glutathione-S-transferase enzymes. The mentioned proteins showed overexpression in mixture 1 (geranyl acetate, α-bisabolol, and nerolidol) treatment at 100 mg/mL each molecule. Possible molecular degradation for a) geranyl acetate, b) α-bisabolol, c) nerolidol. (SG: Glutathione).


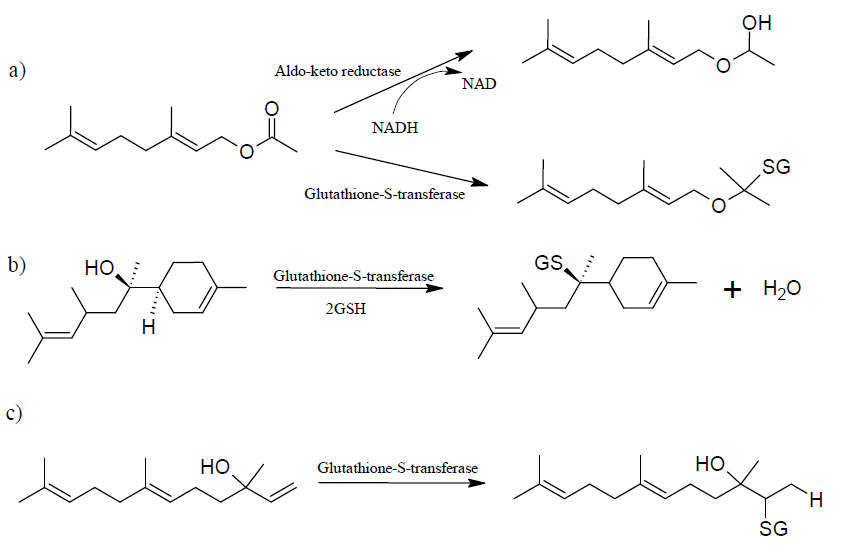

Supplement: Supplementary file 1 — Supplementary Information. [file 41598_2022_24923_MOESM1_ESM.docx]
